# Sulphur Analogues of Homoisoflavonoids as Potential Treatments for Neovascular Eye Diseases

**DOI:** 10.1002/cmdc.202500824

**Published:** 2026-01-31

**Authors:** Jacob D. Hiles, Ola Deri, Kamakshi Sishtla, Joseph C. Bear, Jeremy K. Cockcroft, Elizabeth I. Opara, Ali A. Al‐Kinani, Raid G. Alany, Timothy W. Corson, Sianne L. Schwikkard

**Affiliations:** ^1^ School of Life Sciences Pharmacy and Chemistry Kingston University Kingston‐upon‐Thames UK; ^2^ Eugene and Marilyn Glick Eye Institute Department of Ophthalmology Indiana University School of Medicine Indianapolis Indiana USA; ^3^ Department of Pharmacology and Toxicology Indiana University School of Medicine Indianapolis Indiana USA; ^4^ Leslie Dan Faculty of Pharmacy University of Toronto Toronto Ontairo Canada; ^5^ Department of Chemistry Christopher Ingold Laboratories University College London London UK; ^6^ School of Human Sciences London Metropolitan University London UK

**Keywords:** angiogenesis, anti‐inflammatory, antiproliferation, cytotoxicity, homoisoflavonoid analogues

## Abstract

Treatment of neovascular eye diseases like age‐related macular degeneration require a compound that is not toxic to ocular cells, that can reduce inflammation and inhibit angiogenesis. Homoisoflavonoids, naturally occurring compounds isolated primarily from the Hyacinthaceae sub‐family of plants, have shown promise as anti‐inflammatories and inhibitors of angiogenesis. A series of sulphur analogues, (3‐benzylidene thiochroman‐4‐ones), were synthesised via a three‐step procedure. These compounds were evaluated for selectivity towards endothelial cells over non‐endothelial cells and their ability to inhibit COX‐II over COX‐I.Their potential anti‐angiogenic activity was assessed using the Matrigel tube formation assay. (*3Z*)*‐3‐[(3‐bromophenyl)methylidene]‐6‐methoxy‐2,3‐dihydro‐4H‐1‐benzothiopyran‐4‐one* (**10**) was most active with respect to anti‐proliferation against human retinal endothelial cells (HREC cells) (GI_50_ 3.07 μM). All demonstrated selectivity with all GI_50_ values against retinal pigment epithelial cell line ARPE‐19 >100 µM, with the exception of (*3Z*)*‐6‐bromo‐3‐[(4‐nitrophenyl)methylidene]‐2,3‐dihydro‐4H‐1‐benzothiopyran‐4‐one* (**12**), which was not toxic to either. (*3Z*)*‐3‐[(3‐bromophenyl)methylidene]‐6‐methoxy‐2,3‐dihydro‐4H‐1‐benzothiopyran‐4‐one* (**10**) showed significant reduction in angiogenesis properties in a Matrigel‐based tube formation assay (38.79%, 5 µM, 56.41%, 10 µM). (*3Z*)*‐6‐bromo‐3‐[(3‐bromophenyl)methylidene]‐2,3‐dihydro‐4H‐1‐benzothiopyran‐4‐one* (**7**) was found to be the most active against COX‐II with inhibition of 22.7 % (4.35 nM). The 3‐benzylidene thiochroman‐4‐ones show promise as antiangiogenic agents, but limited selectivity to COX‐II over COX‐I.

## Graphical abstract

Neovascular eye diseases are characterised by the abnormal growth of often fragile blood vessels in the eye. Treatment focuses on reducing angiogenesis as well as reducing oxidative stress induced inflammation, a key underlying cause. Synthetic sulphur analogues of naturally occurring homoisoflavonoids, synthesised in three steps, have shown promise as antiangiogenic and anti‐inflammatory agents in vitro.

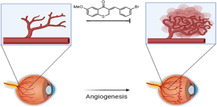

## 1 Introduction

Angiogenesis, the formation of new blood vessels, plays an important role in numerous biological processes, offering both substantial benefits and potential risks [1]. Neovascular diseases of the eye, such as macular degeneration, are characterised by the excessive formation of blood vessels that tend to be leaky and fragile [2]. The principal method of treatment is the use of biologics such as bevacizumab, ranibizumab, and aflibercept that target the vascular endothelial growth factor (VEGF) [3]. These treatments are expensive, need to be administered via intravitreal injections and approximately 30% of people suffering from wet age‐related macular degeneration(AMD) are resistant to these treatments [4]. In addition, inflammation plays a crucial role in these conditions. The effect of long‐term oxidative damage and resulting inflammation has been implicated in the development of wet AMD. In the short term, inflammation plays a protective role, but long‐term inflammation is related to a number of age‐related diseases, including wet AMD [5]. The development of a small molecule that can target both angiogenesis as well as inflammation would significantly improve treatment options for diseases such as wet AMD.

**SCHEME 1:**
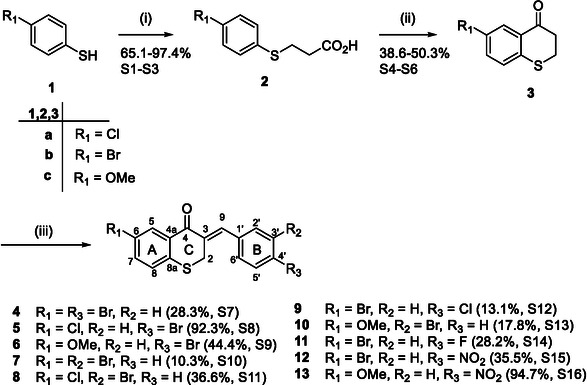
Reagents and conditions: (i) 3‐chloropropionic acid, NaOH, 100°C, 3 h; (ii) PPA, 60°C, 3 h; and (iii) substituted benzaldehyde, H_2_SO_4_, AcOH, rt, 24 h.

Homoisoflavonoids are a naturally occurring, C‐16 sub class of flavonoids that are primarily extracted and isolated from 6 plant families: Asparagaceae, Fabaceae, Polygonaceae, Portulacaceae, Orchidaceae, and Gentianaceae [6]. These compounds have generated interest due to their range of biological activities, including cytotoxicity, anti‐inflammatory, and anti‐angiogenic activities [6]. In particular the application of anti‐angiogenic activity to the potential treatment of wet AMD has stimulated some significant research activity and some promising results [6]. Since cremastranone, a naturally occurring homoisoflavonoid isolated from the orchid *Cremastra appendiculata* as well as from *Muscari* and *Merwilla* spp (Hyacinthaceae), showed good inhibition of angiogenesis both in vitro and in vivo, homoisoflavonoids from natural and synthetic sources have been targeted as potential treatments for neovascular eye diseases [7, 8, 9, 10]. Homoisoflavonoids isolated from *Rhodocodon* spp, with the unusual S‐configuration at C‐3 (Scheme 1) showed inhibition of cell migration in a scratch wound assay and reduction of tubule formation in a Matrigel assay [11]. Synthetic homoisoflavonoids have been synthesised with a wide range of functional groups, with some studies indicating that a double bond between C‐3 and C‐9 (Scheme 1) can enhance activity [12]. The introduction of bulky groups in ring B (Scheme 1), particularly noted in the homoisoflavonoid SH‐11 037 improved activity as shown both in vitro and in initial in vivo studies [13].

Thiochroman‐4‐one derivatives of the naturally occurring homoisoflavonoids (chroman‐4‐ones) are likewise of interest due to their potential as anti‐microbial agents as well as their cytotoxicity and antileishmanial activities [14, 15, 16]. This work is an extension of our research into the bioactivity of natural homoisoflavonoids (oxygen analogues) and investigates the effect of the substitution of the oxygen atom in the C ring of the homoisoflavonoid with a sulphur atom on antiangiogenic and anti‐inflammatory properties. A variety of substituents in addition to sulphur were investigated, including the introduction of halogens and electron‐withdrawing groups such as nitro‐groups. The double bond between C‐3 and C‐9 was maintained.

## 2 Results and Discussion

### 2.1 Chemistry

3‐Benzylidenethiochroman‐4‐ones have been synthesised by several methods, the most popular being the use of either an acid or base catalysed Claisen–Schmidt condensation. Al Nakib et al. [17] found that the use of an acid catalyst, namely HCl gas and ethanol, worked well for the sulphur analogues, while a base catalyst, sodium hydroxide in ethanol, worked better for the oxygen analogues. Mondal et al. used a microwave‐assisted Claisen–Schmidt condensation with anhydrous potassium carbonate as the base [18]. This was successfully done in the solid state with reaction times of 1.5 min for the oxygen analogues. The sulphur analogues, after producing the desired exocyclic *α* *β*‐unsaturated ketone, underwent isomerisation to form the corresponding 3‐benzylthiochromones. The range of 3‐benzylidenethiochroman‐4‐ones investigated in this study was synthesised (Scheme 1) from the corresponding thiophenol and 3‐chloropropionic acid, with sodium hydroxide (40% aqueous solution) as the base, to produce the phenyl sulfanylpropionic acids. These were cyclised with polyphosphoric acid (PPA) to form the thiochromanones. The final step was the Claisen–Schmidt condensation with a range of substituted benzaldehydes. This reaction was found to be slow and low yielding with a base catalyst (yields generally below 10% after chromatography), but it worked efficiently when an acid catalyst was used (concentrated sulphuric acid and glacial acetic acid). The final products were easily isolated by precipitating with methanol.

The introduction of a halogen atom is a well‐researched strategy in medicinal chemistry, with bromine receiving less attention than chlorine and fluorine. Halogen atoms can improve drug‐receptor interactions through the formation of halogen bonds with the protein backbone of target proteins, in particular via Lewis acid–base interactions with the carbonyl group on significant amino acids. Halogen atoms, particularly the larger halogens like bromine, increase lipophilicity and, as such, the ability to cross cell membranes [19]. With bromine being successfully incorporated into a number of commercially available drugs, such as antihistamines (brompheniramine, dexbrompheniramine, and bromodiphenhydramine), anti‐cancer agents (Pipobroman and Vandetanib), and cardiac drugs (bretylium), as well as ophthalmic drugs (Brimonidine), bromine was chosen as a key substituent in our present study [20].

**FIGURE 1:**
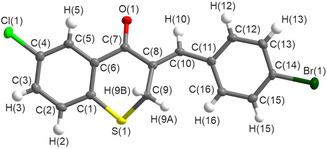
Labelled structure of C_16_H_10_BrClOS at 150 K as determined by single crystal X‐ray diffraction.

Ten different 3‐benzylidenethiochroman‐4‐ones were synthesised and characterised via standard spectroscopic techniques. Compound **5** was crystalline, and the structure was confirmed via single‐crystal X‐ray diffraction analysis, shown in Figure 1. Scheme 1 shows the reaction scheme, products, % yields, and location of relevant data in the supplementary information (S7–S16).

The crystal structure of C_16_H_10_BrClOS (compound **5**) at 150 K crystallised in the monoclinic *P*2_1_/*n* space group; however, it is noticeable both from Figure S17 and the unit cell parameters that while *α* and *γ* are fixed at 90°, *β* also is very close to 90° (90.338(2)°). The molecules of C_16_H_10_BrClOS themselves are very flat, with a puckered ring leading to a torsion angle of 132.0(2)° between atoms S(1)—C(9)—C(8)—C(10). This flatness allows the molecules to pack almost directly on top of one another, as seen when viewing down **a** (Figure S17).

### 2.2 Pharmacological Studies

Naturally occurring homoisoflavonoids have shown promise as anti‐angiogenic agents and initial in vivo studies have shown some success in significantly reducing choroidal neovascularisation (CNV) lesion volume in the laser‐induced CNV mouse model when compared with an anti‐VEGF antibody (anti‐VEGF_164_ antibody) [21]. Eye diseases such as wet AMD are characterised by excessive neovascularisation and, as such this is a good indicator of potential success as a treatment. A successful candidate molecule would ideally show low toxicity within the therapeutic range over a number of ocular cell lines, as well as showing potential as an antiangiogenic agent, and in addition, would reduce inflammation. The compounds synthesised in this study were initially assessed for selective antiproliferative activity on human retinal endothelial cells (HRECs) compared to human retinal pigment epithelial cells (ARPE‐19 cells). In vitro antiangiogenic activity was determined using human umbilical vein endothelial cells (HUVECs) in a Matrigel assay. The most promising compounds were first tested for toxicity towards HUVECs in order to determine the most appropriate concentrations to use for the Matrigel assay. Finally, the compounds were tested to ascertain their anti‐inflammatory activity via selectivity for COX‐II over COX‐I.

#### 2.2.1 Anti‐Proliferative Activity via alamarBlue Assay

The anti‐proliferative activity of the synthesised homoisoflavonoids was determined via the alamarBlue assay against two cell types, HRECs and ARPE‐19 cells. HREC play a key role in the formation of blood vessels in the retina and as such, the inhibition of these cells is a good initial indication of potential anti‐angiogenic activity. ARPE‐19 are involved in photoreceptor function in the eye and as such, testing the compounds against these cells gives a good indication as to whether the compounds are generally cytotoxic or selectively inhibitory towards HREC's. All homoisoflavonoids in the present study (**4–** **13**), with the exception of compound **12**, demonstrated a strong selectivity for HRECs over ARPE‐19 cells, with all GI_50_ values against ARPE‐19 cells >100 μM. Homoisoflavonoid **10** exhibited the greatest anti‐proliferative activity against HRECs with a GI_50_ value of 3.07 μM (Table 1). The active 3‐benzylidene thiochroman‐4‐ones synthesised all demonstrated good selectivity, with compounds **6**, **8**, **10**, and **11** showing particularly good selectivity and activity against the endothelial cells (ECs). Results were comparable with similar synthetic oxygen analogues with a double bond between C‐3 and C‐9 (GI_50_ 0.21–3.60 μM) [12]. As compound **12** showed no activity, it was not tested further. Figures S19–S28 provide the GI_50_ calculations and plots for compounds **4** to **13**.

**TABLE 1:** Anti‐proliferative GI_50_ values for synthesised compounds against HRECs and ARPE‐19 cell line (Mean ± SD). Compounds tested at 0.1 nM, 1 nM, 10 nM, 100 nM, 1 µM, 10  µM, and 100 μM concentrations. The results are from 3 independent experiments.

Compound	HREC GI_50_, μM	ARPE‐19 GI_50_, μM
**4**	17.8 ± 12.4	>100
**5**	11.2 ± 3.12	>100
**6**	4.06 ± 0.53	>100
**7**	10.0 ± 2.70	>100
**8**	4.11 ± 0.52	>100
**9**	15.7 ± 2.22	>100
**10**	3.07 ± 0.38	>100
**11**	3.65 ± 0.17	>100
**12**	>100	>100
**13**	33.7 ± 19.4	>100

#### 2.2.2 Anti‐Angiogenic Activity

##### 2.2.2.1 Determination of Therapeutic Range

**FIGURE 2:**
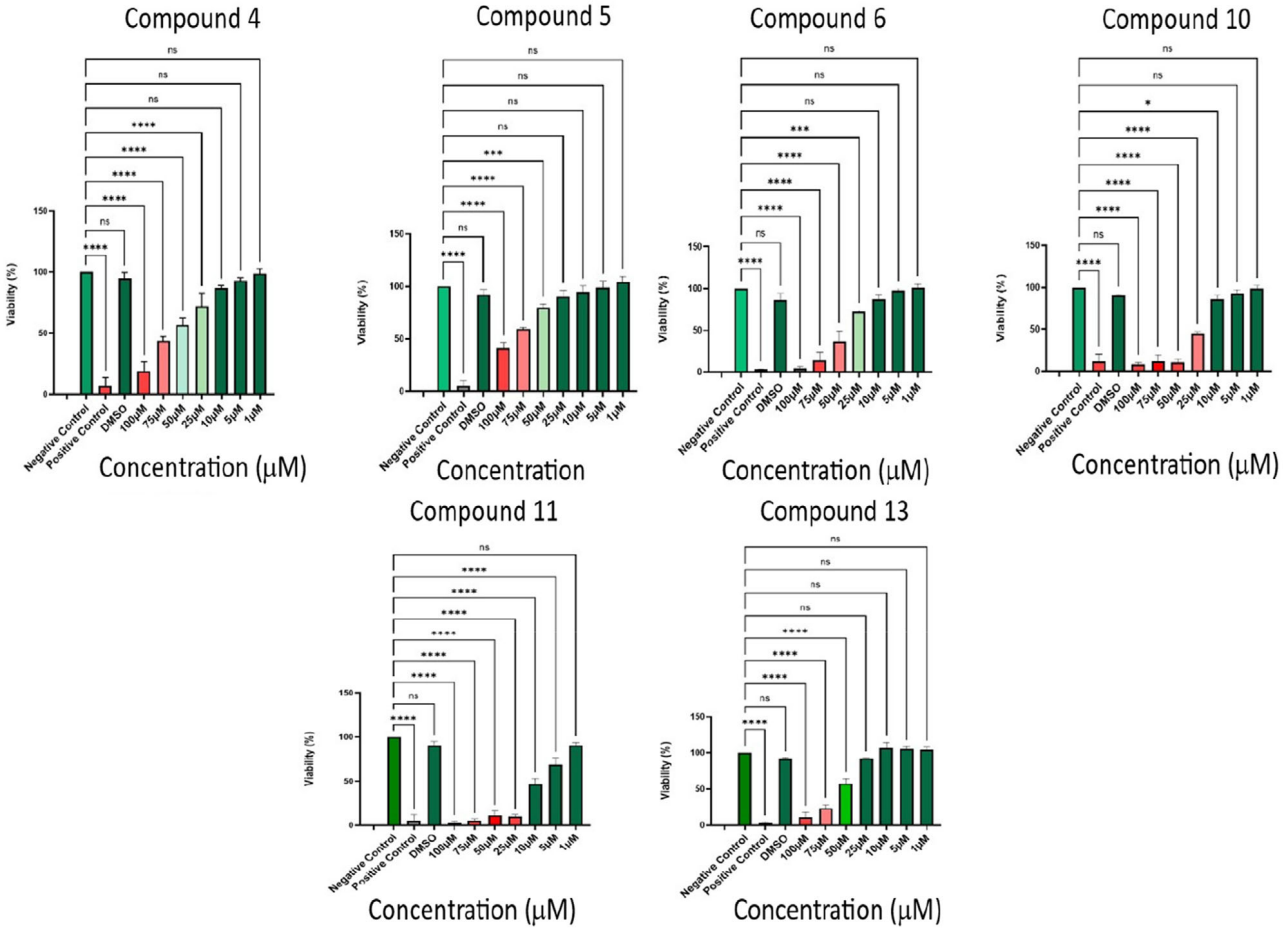
Viability results in % (mean, standard deviation) when tested on HUVECs at seven different concentrations. Results presented as mean ± SD of three independent experiments (one‐way ANOVA against negative control). *p ≤ 0.01, ***p ≤ 0.0004, ****p ≤ 0.0001.

Six compounds, ranging from the most to the least selective based on the anti‐proliferative studies (**4**, **5**, **6**, **10**, **11**, and **13**), were evaluated at seven concentrations (1, 5, 10, 25, 50, 75, and 100 μM) for their cytotoxicity against HUVECs using the neutral red assay (Table 2, Figure 2) [22]. HUVECs are a commonly used cell type due to their wide availability, relatively strong proliferative capacity and well‐defined angiogenic characteristics [23]. It was established that almost all tested compounds showed a significant toxicity on HUVECs when used in concentrations of 50 µM and higher. However, none of the compounds showed any cytotoxicity at the three lowest tested concentrations (1, 5, and 10 µM). Since an acceptable viability was identified with concentrations of 5 and 10 µM, these two concentrations were used to test potential anti‐angiogenic activity using a Matrigel tube formation assay with HUVECs.

**TABLE 2:** Cytotoxicity of homoisoflavonoids against HUVECs. IC_50_ values given in μM (Mean ± SD). Compounds tested at 1, 5, 10, 25, 50, 75, and 100 µM, results show the average of 4 repeats of 3 independent experiments. H_2_O_2_ used as positive control and at a concentration of 0.1 mM produced 90 ± 6.54% inhibition.

Compound	HUVEC IC_50_, μM
**4**	60.81 ± 1.78
**5**	90.33± 6.36
**6**	46.86 ± 4.72
**10**	38.22± 3.48
**11**	15.97± 3.98
**13**	57.15± 4.23

##### 2.2.2.2 Determination of Inhibition of Tube Formation

The same six compounds (**4**, **5**, **6**, **10**, **11**, and **13**) were tested for their inhibitory effects on tubule formation using HUVECs in a Matrigel‐based tube assay.

Matrigel tube formation assay was first published in 1988 as a screening model for evaluating the angiogenic inhibitory or promoting properties of a tested compound [24]. Matrigel is a gelatinous protein mixture derived from Engelbreth–Holm–Swarm (EHS) mouse sarcoma cells. Matrigel is used to investigate the angiogenic activity of a compound as it closely resembles the extracellular matrix (ECM) and facilitates the adhesion, growth, proliferation, and differentiation of ECs, making it a valuable tool for measuring in vitro angiogenesis. The angiogenic activity can be assessed by measuring the formation of tubular structures from vascular ECs.

**FIGURE 3:**
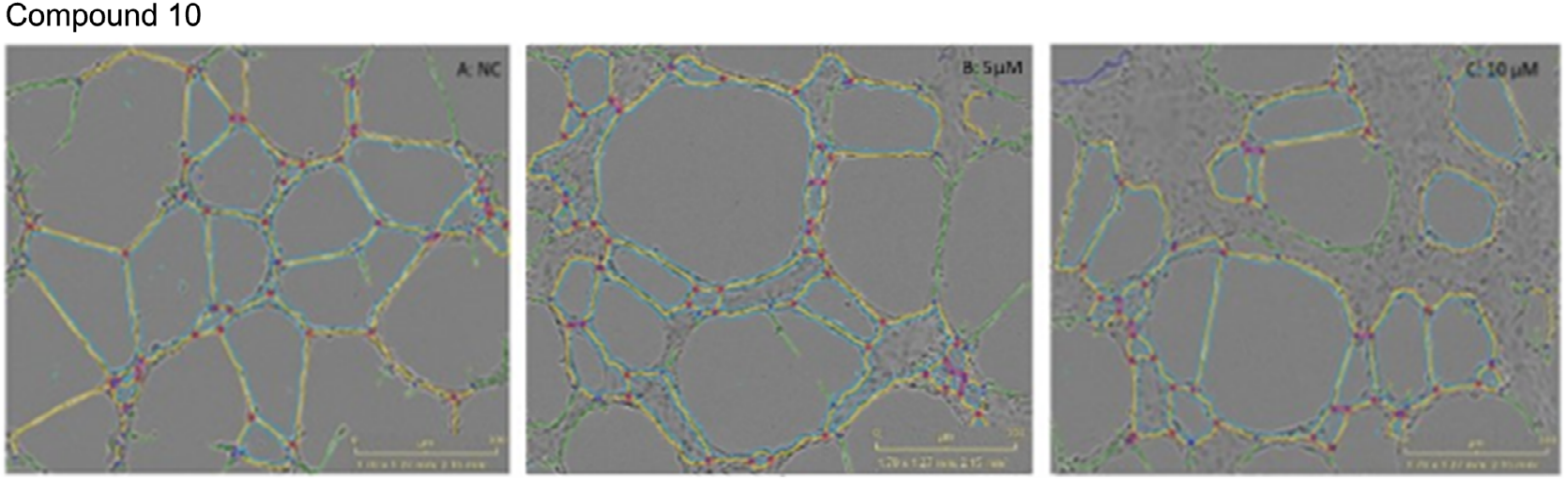
Comparative network development in tube formation assay where; (A): represents the negative control, (B): compound 10 at 5 µM, and (C): compound 10 at 10 µM. Images were analysed using Incucyte software, data was statistically analysed with Graph Pad Prism.

**FIGURE 4:**
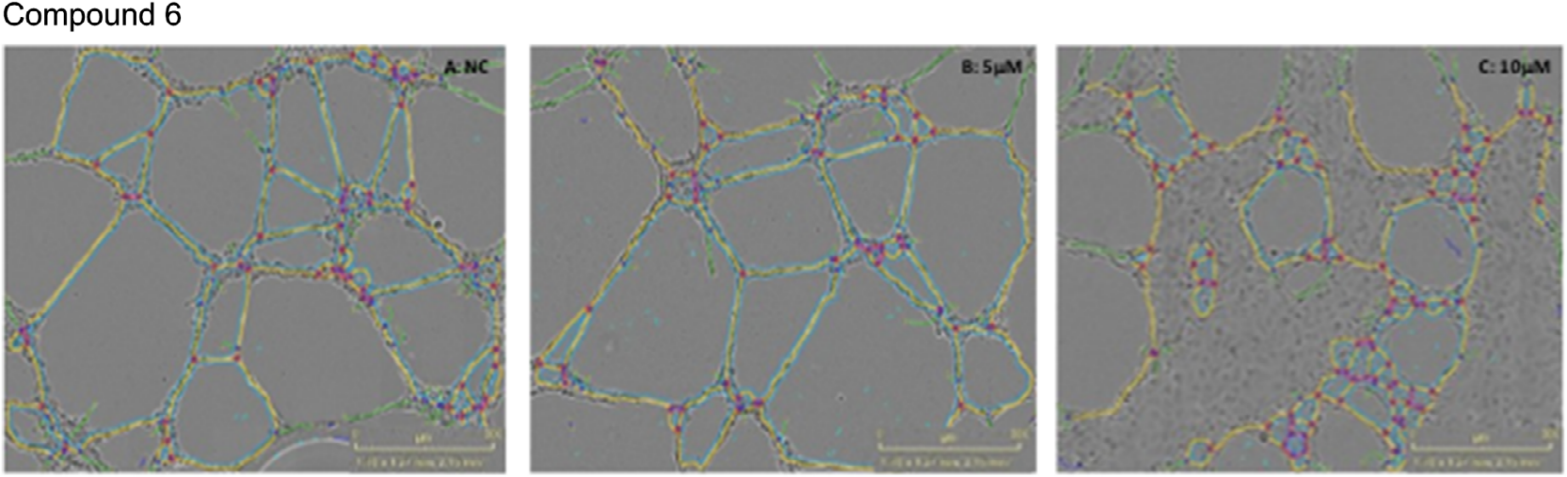
Comparative network development in tube formation assay where; (A): represents the negative control, (B): compound 6 at 5 µM, and (C): compound 6 at 10 µM. Images were analysed using Incucyte software, data was statistically analysed with Graph Pad Prism.

In our assay, pictures were taken at 0 and 6 h using an Incucyte, then analysed with the image analysis software ‘Image J’. The obtained quantitative data were evaluated, focusing on two parameters against the negative control (cells in complete media), the total tube length and the number of meshes. Total tube length indicates the cumulative length of all tubes formed; therefore, any reduction in this number indicates an impaired angiogenic process. Meshes or loops are defined as the closed polygonal areas formed by interconnected tubes. Fewer number of meshes suggests compromised capillary‐like network complexity, thus reduced angiogenic activity [25]. Five of the six compounds (**4**, **6**, **10**, **11**, and **13**) demonstrated a significant reduction in the number of meshes observed (Figure 3, images from the incucyte can be found in S29), with compound **10** resulting in the most significant reduction at 5 µM (38.8%) and at 10 µM (56.4%) compared to the control (Figure 3). Compounds **6** and **13** both exhibited a similar reduction in the number of meshes (27.6% and 24.3% respectively), albeit at the concentration of 10 µM. The results for compound **6**, which are comparable to compound **13**, are shown in Figure 4. Compound **11** showed some activity, represented in dropping the number of meshes formed by 22.2% but only at the higher concentration of 10 µM. Compound **5** did not demonstrate any significant reduction in the number of meshes at either concentration. Since compounds **6**, **10**, and **13** all displayed some decrease in forming meshes and taking into consideration that these compounds share the same R_1_ substituent in their structure (methoxy group), the presence of the methoxy moiety at the R_1_ position on the A ring may induce an enhanced antiangiogenic activity to homoisoflavonoids.

**FIGURE 5:**
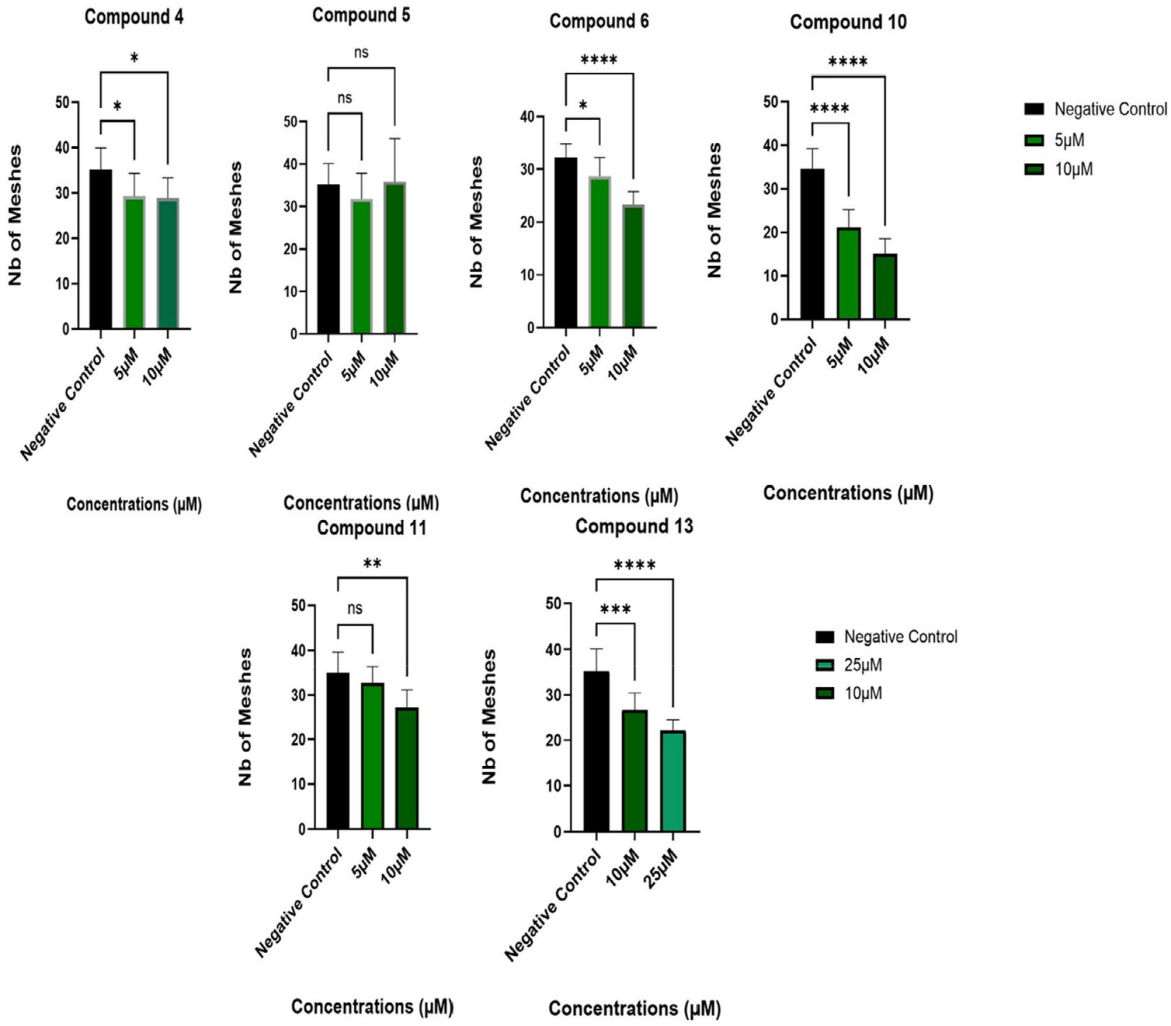
Comparative network parameter (the number of meshes) in the tube formation assay, images were analysed using Image J, data as shown were statistically analysed with Graph Pad Prism against negative control (NC): (cells/complete media). Results presented as mean ± SD of three independent experiments (one‐way ANOVA against negative control). **p ≤ 0.05.

**FIGURE 6:**
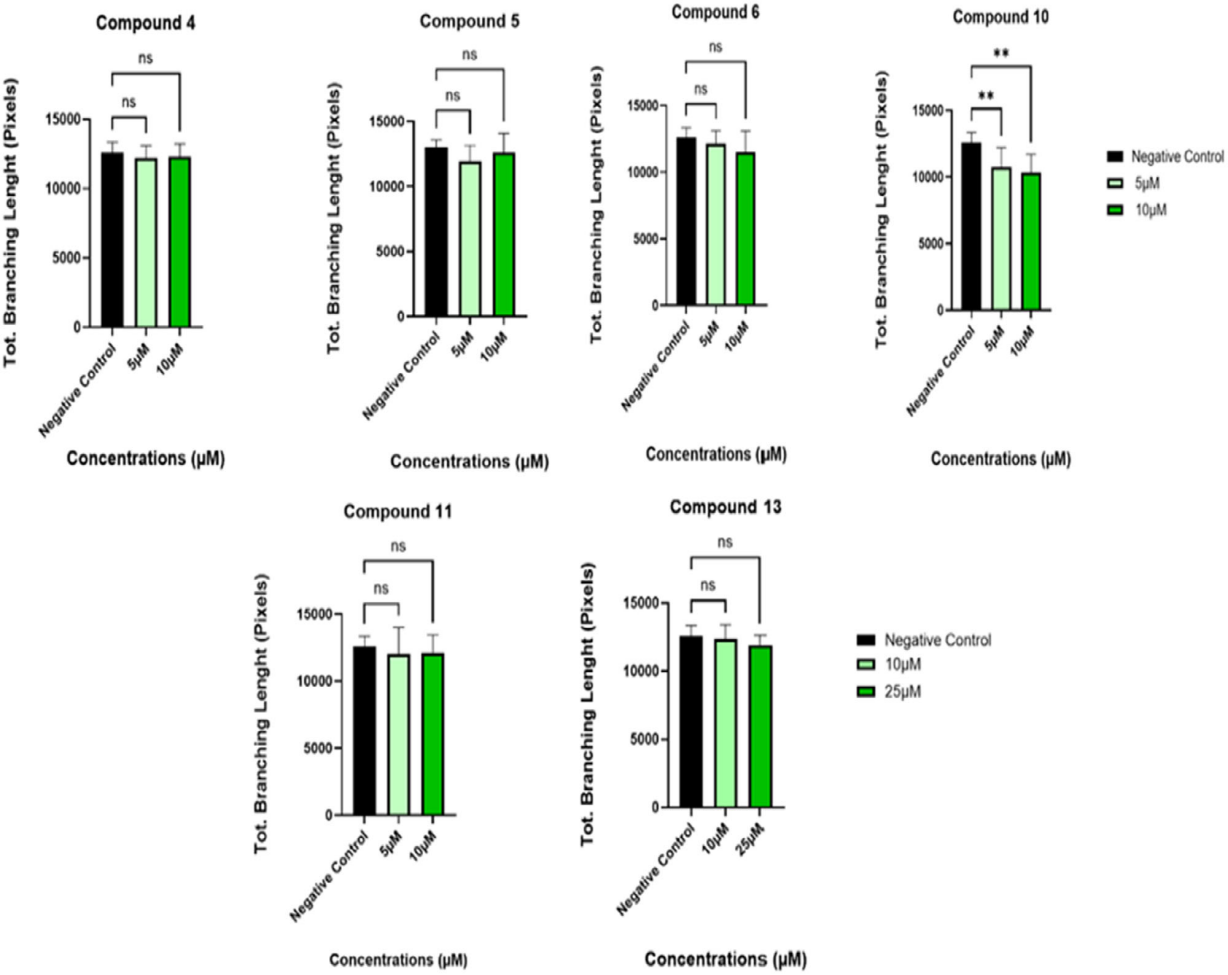
Comparative network parameter (Tot. Branching Length) in the tube formation assay, images were analysed using Image J, data as shown were statistically analysed with Graph Pad Prism. Results presented as mean ± SD of three independent experiments. **p ≤ 0.05.

Although compounds **4**, **6**, **10**, **11**, and **13** all demonstrated a significant reduction in the number of meshes observed (Figure 5), only compound **10** presented a significant reduction in the total branching length at both concentrations tested (5 and 10 µM) (Figure 6). All images for the inhibition of tube formation can be found in the supplementary information (Figure S29). The images shown above (Figures 3 and 4) for compounds **10** and **6** are representative of the results obtained.

#### 2.2.3 COX Inhibitory Activity

Selective activity against cyclooxygenase‐II (COX‐II) is an attractive research target. Prostaglandins (PGs) are biosynthesised from arachidonic acid with the cyclooxygenase enzymes (COX) forming part of the bifunctional PG H synthase enzyme. COX is responsible for the addition of two oxygen molecules as arachidonic acid is converted to PG H_2_ (PGH_2_) via PG G_2_ (PGG_2_). Many anti‐inflammatories do not discriminate between the two isoforms (COX‐I and COX‐II) and as such, the inhibition of the constitutively important COX‐I isoform can lead to side effects such as gastrointestinal discomfort [26]. In addition, COX‐II is implicated in angiogenesis and is overexpressed in many forms of cancer, in particular colon cancer and as such, an ability to selectively inhibit COX‐II has therapeutic potential [27]. There is an established relationship between PGs and angiogenesis, with PGs being pro‐angiogenesis, mediated by interaction with VEGF [28]. Selective inhibition of COX‐II has been shown to reduce retinal angiogenesis [29, 30]. Previous work has shown homoisoflavonoids from the *Rhodocodon* species to selectively inhibit COX‐II with activity similar to aspirin [26]. The homoisoflavonoids isolated from *Massonia pustulata* and *Ledebouria ovatifolia* likewise demonstrated selective COX‐II inhibitory activity. [28] It was of interest to us to determine whether the substitution of the oxygen atom in the B‐ring with a sulphur atom would have any effect on the activity or selectivity of this inhibition. Six homoisoflavonoids (**4**, **5**, **6**, **7**, **8**, and **10**) were screened for their COX inhibitory activity against both COX‐I and COX‐II isoforms (Table 3). The homoisoflavonoids were tested at two concentrations, at 4.35 and at 2.18 nM as determined by the kit used (Cayman Chemicals). Homoisoflavonoid **5** exhibited the greatest COX‐I inhibitory activity at 4.35 nM with 34.3% inhibition. Homoisoflavonoid **7** exhibited the greatest COX‐II inhibition with 22.7% inhibition at 4.35 nM; however, homoisoflavonoid **7** lacked selectivity as at 4.35 nM, COX‐I was inhibited by 31.3%. Homoisoflavonoids **8** and **10** displayed COX‐I inhibition with inhibitions of 27.5% and 24.3% at 4.35 nM. The sulphur analogues of homoisoflavonoids showed promise as anti‐inflammatory agents, but did not show a marked selectivity towards the inhibition of COX‐II over COX‐I.

**TABLE 3:** COX inhibition of sulphur‐containing homoisoflavonoids tested at two concentrations of 4.35 and 2.18 nM as determined by the Cayman Chemicals COX inhibition kit. Compounds **4**, **5**, **7**, and **10** exhibited statistically significant differences between the two concentrations for COX‐I with *p* values < 0.05. Compounds **4**, **6**, **7**, and **10** exhibited statistically significant differences between the two concentrations for COX‐II with *p* values < 0.05.

	COX‐I Inhibition %		COX‐II Inhibition %	
Compound	4.35 nM	2.18 nM	4.35 nM	2.18 nM
**4**	26.2 ± 3.96	9.42 ± 2.21	5.60 ± 1.15	0
**5**	34.3 ± 3.68	3.48 ± 2.63	3.89 ± 1.51	0
**6**	28.7 ± 4.95	7.83 ± 4.90	13.5 ± 2.55	0
**7**	31.3 ± 2.86	5.65 ± 2.26	22.7 ± 1.78	6.61 ± 1.54
**8**	27.5 ± 4.27	14.8 ± 4.86	0	0
**10**	24.3 ± 2.35	9.67 ± 1.12	0	0

## 3 Conclusions

In conclusion, the synthesis of ten sulphur homoisoflavonoid analogues was accomplished. Nine of the active homoisoflavonoid analogues synthesised in the present study demonstrated strong selectivity for HRECs over ARPE‐19 cells. The results were comparable with similar synthetic oxygen analogues with a double bond between C‐3 and C‐9 (GI_50_ 0.21–3.60 μM) [12]. This suggests the sulphur analogues may be useful for ocular anti‐angiogenic applications due to the role that the HRECs play in the formation of retinal blood vessels and the role that this plays in the development of various angiogenesis‐associated eye diseases, including proliferative diabetic retinopathy, and wet AMD [31]. These compounds demonstrated that they had the ability to inhibit angiogenesis on a Matrigel based assay at concentrations not toxic to HUVECs. The selectivity for ECs over non‐ECs, together with the COX inhibitory activity and ability to inhibit HUVEC tube formation at non‐toxic concentrations, make these benzylidene thiochromanones excellent candidates for further development as potential treatments for wet AMD and related neovascular eye diseases.

## 4 Experimental

### 4.1 General

The NMR spectra were recorded on the Bruker Avance III 400 two channel FT‐NMR spectrometer (AV400). Spectra were analysed using Topspin 3.6.1. Low‐resolution mass spectra of compounds were obtained using Agilent technologies network mass selective detector 5987. High resolution mass spectra were obtained from the national mass spectrometer facility, Swansea, using the Xevo G2‐XS QTof Quadrupole Time‐of‐Flight Mass Spectrometer. Infrared spectra were recorded on Thermo Scientific Nicolet is5 with ATR attachment FTIR spectrometer. Melting points were recorded using the SANYO Gallenkamp melting point apparatus. X‐ray crystallography: a single crystal of compound **5** was grown from toluene at room temperature. The single crystal data measurements were made using a twin‐source SuperNova diffractometer with a microfocus Cu X‐ray beam (50 kV, 0.8 mA) and an Atlas (135 mm CCD) detector. The temperature of the sample was controlled using an Oxford Instruments Cryojet5. The data obtained were processed using the CrysAlisPro software package (version 1.171.40.60a) and Rigaku Oxford Diffraction. Full details are available in the Supporting Information. All chemicals were purchased from Sigma–Aldrich (Poole, Dorset) and used without further purification.

### 4.2 General Procedure for the Synthesis of Substituted Phenylsulphanyl Propanoic Acids

Substituted thiophenol (0.01 mol) and *β*‐chloropropanoic acid (1.30 g, 0.012 mol) were dissolved in a NaOH solution (50 mL, 0.24 mol). The system was refluxed at 100°C for 3 h under stirred conditions. The reaction was monitored by TLC (1:1 hexane:EtOAc). Once complete**,** the reaction was cooled to room temperature and the solution was adjusted by dilute HCl to pH = 1, causing a white solid to precipitate. The solids formed were isolated via vacuum filtration and dried to yield the desired product. Full characterisation data can be found in Figures S1–S3.

### 4.3 General Procedure for the Synthesis of Substituted Thiochromanones

PPA (20 g, 0.20 mol) was added to a flask containing substituted phenylsulphanyl propanoic acids (4.62 mmol). The reaction mixture was heated at 60°C for 3 h under mechanical stirring. The reaction was then quenched with ice and left to stir for a further 10 min. The mixture was extracted with ethyl acetate (4 × 30 mL). The combined organic layers were washed once with distilled water, followed by a NaOH solution (5%) wash and repeatedly washed with distilled water until a pH = 7 was acquired. The organic layers were washed with brine, then dried over MgSO_4_. The reaction mixture was concentrated under reduced pressure, yielding a light orange solid. Full characterisation data can be found in Figures S4–S6.

### 4.4 General Procedure for the Synthesis of the Sulphur Homoisoflavonoid Analogues

To a solution of the substituted thiochromanone (2.4 mmol) and substituted benzaldehyde (2.2 mmol) in glacial acetic acid (2 mL), concentrated H_2_SO_4_ (0.6 mL) was added. The reaction was stirred at room temperature for 24 h. Progress was monitored by TLC (1:1 EtOAc:hexane). Once complete, methanol was added, and solids precipitated. The solids were filtered and dried yielding a fine powder. Spectroscopic data, including HRMS can be found in the supplementary data (S7–S16 and S30).

### 4.5 Crystallographic Data

Deposition Number(s) https://www.ccdc.cam.ac.uk/services/structures?id=doi:10.1002/cmdc.202500824%22%3E2421737 contain(s) the supplementary crystallographic data for this article. These data are provided free of charge by the joint Cambridge Crystallographic Data Centre and Fachinformationszentrum Karlsruhe http://www.ccdc.cam.ac.uk/structures. All crystallographic data tables can be found in the supplementary information, Table S1a–S1e.

### 4.6 Anti‐Proliferative Activity via the alamarBlue Assay

HRECs (Cell Systems) and ARPE‐19 cells (ATCC) were plated at 2,500 cells/well in their respective growth media (EGM‐2 mv [Lonza] or Ham's‐F10 (Thermo) + 10% FBS + 1% penicillin‐streptomycin) (100 μL). Cells were incubated in the centre of 48‐wells of 96‐well clear bottom black plates overnight, with the surrounding wells containing deionised, sterilised water (100 μL). This was followed by treatment of cells with 1 μL of different concentrations of each test compound. Compounds were tested in triplicate over the range of 1 mM to 1 nM (1% v/v final DMSO concentration). Treated cells were incubated for a further 44 h. At the end of this incubation period, alamarBlue reagent (11.1 μL) was added and after 4 h of incubation, fluorescence readings were taken with excitation and emission wavelengths of 560 and 590 nm, respectively. Data were analysed and dose response curves generated using GraphPad Prism software (v. 10.0).

### 4.7 Cyclooxygenase Inhibition

The inhibition of cyclooxygenase was determined via the COX (ovine/human) Inhibitor Screening Assay Kit Item № 560 131 (Cayman Chemical Company, Ann Arbour, MI, USA). COX inhibition was assayed using the manufacturer's guidelines via the direct measurement of PGF_2_ *α* by the reduction of COX‐derived PGH_2_ by SnCl_2_. To a series of supplied reaction buffer solutions (0.1 M Tris‐HCl, pH = 8, containing 5 mM EDTA and 2 mM phenol) with haem, either COX‐I or COX‐II enzyme and two concentrations (4.35 and 2.18 nM) of inhibitor samples (homoisoflavonoids synthesised in this study) were added. The solutions were subsequently incubated at 37°C for 5 min. After 5 min, the substrate arachidonic acid was added. After a further 2 min, saturated stannous chloride solution was added to stop the reaction. The COX inhibition assay is based upon the competition between PGs and a PG tracer (PG‐acetylcholinesterase conjugate) for a limited amount of PG antiserum. Due to the concentration of the PG tracer being constant whilst the concentration of the PG varies, the amount of PG tracer able to bind to the PG antiserum is inversely proportional to the concentration of PG in the well. The plates were washed to remove any unbound reagents in the wells. Subsequent addition of Ellman's reagent (substrate for the excess acetylcholine esterase) allowed for a distinctive yellow colour to appear. This yellow colour is generated due to the enzymatic reaction product. To determine the intensity of the yellow colour, an ELISA plate reader (BioTek EPOCH 2 microplate reader) was utilised at a wavelength of 405 nm. As the colour is proportional to the amount of PG tracer bound to the well, which is inversely proportional to the amount of PGs present in the well, this allows for the determination of percent inhibition via the use of multiple control incubations. The COX inhibition test was run in duplicate and the results were analysed via the two sample t‐test.

### 4.8 Cytotoxicity Activity via the Neutral Red Assay against HUVECs

Under sterile conditions, compounds were serially diluted from stock solutions to give the desired concentration of each compound prepared (1, 5, 10, 25, 50, 75, and 100 µM).

HUVECs (ATCC) were cultured in a 96‐well plate. Ten thousand cells were seeded per well and incubated until reaching 80% confluency. Culture medium was aspirated without disrupting the monolayer, and cells were washed with pre‐warmed culture medium to remove any traces of floating or dead cells. A total volume of 100 µL of treatment solutions was added to each of the wells, one positive control (H_2_O_2_) at 0.1 mM and two negative controls (cells/media, cells/media/DMSO) were included. Four repeats of three independent experiments *N* = 3 were carried out. After adding controls and treatment solutions, cells were incubated for 24 h at 37°C, 5% CO_2_.

Neutral red stock solution (4 mg/mL) was diluted with media to give a 40 µg/mL solution. This was followed by an overnight incubation at the same temperature as the cells, allowing any dye crystals to precipitate and settle at the bottom of the tube [20].

After 24 h, cultures were examined under a phase‐contrast inverted microscope, noting any changes in the morphology of the cells. Treatment solutions were removed and cells were washed with pre‐warmed media followed by dispensing 100 µL of neutral red dye solution (40 µg/mL) (100 µL) per well. Plates were incubated for 3 h. After the incubation period, neutral red medium was removed and plates were washed with PBS (100 µL per well) followed by the addition of the de‐stain solution (50% ethanol 96%, 49% deionised H_2_O, 1% glacial acetic acid) (100 µL) per well. Plates were shaken on a plate shaker for 10–15 min, allowing the neutral red dye to be extracted from viable cells and form a homogeneous solution.

Absorbance (OD) of the extracts was measured in each well using a spectrophotometer at a wavelength of 540 nm. Blanks of wells with no cells were used as a reference. Data were calculated from 3 independent experiments, *N* = 3.

Cell viability was calculated using the equation:

$$\text{Cells Viability}\%=\frac{\mathrm{OD}\left(\mathrm{DMSO}\right)-\mathrm{OD}\left(\text{sample}\right)}{\mathrm{OD}(\mathrm{DMSO})}\times 100\%$$

Finally, data were analysed using GraphPad Prism and by running one‐way ANOVA with multiple comparisons against the negative control. A difference in mean values between compared groups was considered to be significant when *p *≤ 0.05.

### 4.9 Tubule Formation Assay (Matrigel)

HUVECs were prepared and cultured in a T‐75 flask until forming a monolayer ≈80% confluent. Sterile 96‐well plates and 200 µL tips were pre‐cooled. Trypsin‐EDTA 25% and complete media of FK‐12 (FBS 10%, ECGS) were prepared and warmed in a water bath at 37°C 1 h before the experiment.

The wells were top‐coated with a layer of Matrigel (50 µL). Prepared plates were placed in the incubator at 37°C, 5% CO_2_ for 60 min, allowing the Matrigel to harden and form a base layer.

A density of 15,000 cells/ well in a total volume of 100 µL/well was used. Prepared treatment volumes were added slowly in the centre of the Matrigel‐coated wells, then placed in the Incucyte at 37°C, 5% CO_2_. Images were taken at 1 h intervals for 10 h. Images were quantified using Image J software using the ‘angiogenesis analyser’ extension tool at 6 h, and two parameters were used to quantify the results (Total mesh number and total branching length).

Statistical analysis was performed using GraphPad Prism, data were expressed as mean ± SEM. One‐way ANOVA for multiple comparisons with one independent variable was implemented. A difference in mean values between compared groups was considered to be significant when *p *≤ 0.05.

## Supporting Information

Additional supporting information can be found online in the Supporting Information section. **Supporting Fig. S1:** ^1^H‐NMR and ^13^C‐NMR spectra of compound **2a**. **Supporting Fig. S2:** ^1^H‐NMR and ^13^C‐NMR spectra of compound **2b. Supporting Fig. S3:** ^1^H‐NMR and ^13^C‐NMR spectra of compound **2c. Supporting Fig. S4:** ^1^H‐NMR and ^13^C‐NMR spectra of compound **3a. Supporting Fig. S5:** ^1^H‐NMR and ^13^C‐NMR spectra of compound **3b. Supporting Fig. S6:** ^1^H‐NMR and ^13^C‐NMR spectra of compound **3c. Supporting Fig. S7:** ^1^H‐NMR and ^13^C‐NMR spectra of compound **4. Supporting Fig. S8:** ^1^H‐NMR and ^13^C‐NMR spectra of compound **5. Supporting Fig. S9:** ^1^H‐NMR and ^13^C‐NMR spectra of compound **6. Supporting Fig. S10:** ^1^H‐NMR and ^13^C‐NMR spectra of compound **7. Supporting Fig. S11:** ^1^H‐NMR and ^13^C‐NMR spectra of compound **8. Supporting Fig. S12:** ^1^H‐NMR and ^13^C‐NMR spectra of compound **9. Supporting Fig. S13:** ^1^H‐NMR and ^13^C‐NMR spectra of compound **10. Supporting Fig. S14:** ^1^H‐NMR and ^13^C‐NMR spectra of compound **11. Supporting Fig. S15:** ^1^H‐NMR and ^13^C‐NMR spectra of compound **12. Supporting Fig. S16:** ^1^H‐NMR and ^13^C‐NMR spectra of compound **13. Supporting Fig. S17:** Unit cell showing the structure of compound **5** (C_16_H_10_BrClOS) (C = grey, Cl =  green, H = white, S = yellow and Br = dark green) at 150 K, as viewed down **a. Supporting Fig. S18:** Photographs of crystals of compound **5** (C_16_H_10_BrClOS). a) is a photograph of the as‐synthesised crystals and b) a single crystal mounted on the nylon loop *in situ* on the single crystal diffractometer. **Supporting Fig. S19:** Biological Data for Compound **4** (a) Cytotoxicity against HeLa cells, (b) anti‐proliferation against HREC, (c) anti‐proliferation against ARPE‐19**. Supporting Fig. S20:** Biological Data for Compound **5** (a) Cytotoxicity against HeLa cells, (b) anti‐proliferation against HREC, (c) anti‐proliferation against ARPE‐19**. Supporting Fig. S21:** Biological Data for Compound **6** (a) Cytotoxicity against HeLa cells, (b) anti‐proliferation against HREC, (c) anti‐proliferation against ARPE‐19**. Supporting Fig. S22:** Biological Data for Compound **7** (a) Cytotoxicity against HeLa cells, (b) anti‐proliferation against HREC, (c) anti‐proliferation against ARPE‐19**. Supporting Fig. S23:** Biological Data for Compound **8** (a) Cytotoxicity against HeLa cells, (b) anti‐proliferation against HREC, (c) anti‐proliferation against ARPE‐19**. Supporting Fig. S24:** Biological Data for Compound **9** (a) Cytotoxicity against HeLa cells, (b) anti‐proliferation against HREC, (c) anti‐proliferation against ARPE‐19**. Supporting Fig. S25:** Biological Data for Compound **10** (a) Cytotoxicity against HeLa cells, (b) anti‐proliferation against HREC, (c) anti‐proliferation against ARPE‐19**. Supporting Fig. S26:** Biological Data for Compound **11** (a) Cytotoxicity against HeLa cells, (b) anti‐proliferation against HREC, (c) anti‐proliferation against ARPE‐19**. Supporting Fig. S27:** Biological Data for Compound **12** (a) Cytotoxicity against HeLa cells, (b) anti‐proliferation against HREC, (c) anti‐proliferation against ARPE‐19**. Supporting Fig. S28:** Biological Data for Compound **13** (a) Cytotoxicity against HeLa cells, (b) anti‐proliferation against HREC, (c) anti‐proliferation against ARPE‐19**. Supporting Fig. S29:** Images of Matrigel experiments for compounds 4, 5, 6, 10, 11 and 13**. Supporting Fig. S30:** HRMS data**. Supporting Table S1a:** Crystal data and structure refinement for compound **5** (C_16_H_10_BrClOS) at 150 K**. Supporting Table S1b:** Fractional atomic coordinates and equivalent isotropic displacement parameters for compound **5** (C_16_H_10_BrClOS) at 150 K. *U*_eq_ is defined as ⅓ of the trace of the orthogonalised *U*_ij_ tensor**. Supporting Table S1c:** Anisotropic displacement parameters for compound **5** (C_16_H_10_BrClOS) at 150 K. The anisotropic displacement factor exponent has the form: −2*π* ^2^[*h* ^2^ a*^2^ *U*_11_+2hka*b*U_12_+…]**. Supporting Table S1d:** Selected bond lengths for compound **5** (C_16_H_10_BrClOS) at 150 K. **Supporting Table S1e:** Selected bond angles for compound **5** (C_16_H_10_BrClOS) at 150 K.

## Funding

This study was supported by the National Institute of Mental Health and Neurosciences (R01EY025641) and Retina Research Foundation, Canada Foundation for Innovation, and Natural Sciences and Engineering Research Council (RGPIN‐2025‐04563).

## Conflicts of Interest

The authors declare no conflicts of interest.

## Supporting information

Supplementary Material

## Data Availability

The data that support the findings of this study are available from the corresponding author upon reasonable request.
