# Supplementary material for: Sulphur Analogues of Homoisoflavonoids as Potential Treatments for Neovascular Eye Diseases
Source: ChemMedChem. 2026 Jan 31;21(2):e202500824. doi: 10.1002/cmdc.202500824 (PMC12860484; doi:10.1002/cmdc.202500824)
Supplement: Supplementary file 1 — Supplementary Material [file CMDC-21-e202500824-s001.pdf]

Supporting Information

to

Sulfur Analogues of Homoisoflavonoids as Potential Treatments for Neovascular Eye Diseases

Jacob D. Hiles[a], Ola Deri[a], Kamakshi Sishtla[b,c,d], Joseph C. Bear[a], Jeremy K. Cockcroft[e], Elizabeth I. Opara[a,f], Ali A. Al-Kinani[a], Raid G. Alany[a], Timothy W. Corson[b,c,d] and Sianne L. Schwikkard[a]*

a Dr J. D. Hiles, Ms O. Deri, Dr J. C. Bear, Professor E. I. Opara, Dr A. A. Al-Kinani, Professor R. G. Alany, Dr S. L. Schwikkard*

School of Life Sciences, Pharmacy and Chemistry, Kingston University, Kingston-upon-Thames, KT1 2EE, UK.

E-mail: s.schwikkard@kingston.ac.uk

b Dr K. Sishtla, Professor T. W. Corson

Eugene and Marilyn Glick Eye Institute, Department of Ophthalmology, Indiana University School of Medicine, 1160 W. Michigan St., Indianapolis, IN 46202, U.S.A.

c Dr K. Sishtla, Professor T. W. Corson

Department of Pharmacology and Toxicology, Indiana University School of Medicine, 1160 W. Michigan St., Indianapolis, IN 46202, U.S.A.

d Dr K. Sishtla, Professor T. W. Corson

Leslie Dan Faculty of Pharmacy, University of Toronto, 144 College Street, Toronto, ON M5S 3M2, Canada

e Dr J. K. Cockcroft

Department of Chemistry, Christopher Ingold Laboratories, University College London, 20 Gordon Street, London WC1H 0AJ, UK.

a,f Professor E. I. Opara

School of Human Sciences, London Metropolitan University, 166-220 Holloway Road, London, N7 8DB

Table of Contents Page No.

NMR Spectra

List of Figures

Figure S1. ^{1}H-NMR and ^{13}C-NMR spectra of compound **2a** S3

Figure S2. ^{1}H-NMR and ^{13}C-NMR spectra of compound **2b** S4

Figure S3. ^{1}H-NMR and ^{13}C-NMR spectra of compound **2c** S5

Figure S4. ^{1}H-NMR and ^{13}C-NMR spectra of compound **3a** S7

Figure S5. ^{1}H-NMR and ^{13}C-NMR spectra of compound **3b** S8

Figure S6. ^{1}H-NMR and ^{13}C-NMR spectra of compound **3c** S9

Figure S7. ^{1}H-NMR and ^{13}C-NMR spectra of compound **4** S10

Figure S8. ^{1}H-NMR and ^{13}C-NMR spectra of compound **5** S11

Figure S9. ^{1}H-NMR and ^{13}C-NMR spectra of compound **6** S13

Figure S10. ^{1}H-NMR and ^{13}C-NMR spectra of compound **7** S14

Figure S11. ^{1}H-NMR and ^{13}C-NMR spectra of compound **8** S16

Figure S12. ^{1}H-NMR and ^{13}C-NMR spectra of compound **9** S18

Figure S13. ^{1}H-NMR and ^{13}C-NMR spectra of compound **10** S19

Figure S14. ^{1}H-NMR and ^{13}C-NMR spectra of compound **11** S21

Figure S15. ^{1}H-NMR and ^{13}C-NMR spectra of compound **12** S22

Figure S16. ^{1}H-NMR and ^{13}C-NMR spectra of compound **13** S23

Single-Crystal X-ray Diffraction (SXD)

SXD Measurements and Analysis S25

Crystallographic Tables on compound **5 (**$C_{16}H_{10}BrClOS$) at 150 K

Table S1a. Crystal data and structure refinement S26
Table S1b. Fractional atomic coordinates and *U*(eq) for all atoms S27
Table S1c. Anisotropic displacement parameters S28
Table S1d. Selected bond lengths S28
Table S1e. Selected bond and torsion angles S28

List of Figures

Figure S17. Crystal structure of compound **5** ($C_{16}H_{10}BrClOS$) as viewed down **a** S29

Figure S18. Photographs of crystals of compound **5** ($C_{16}H_{10}BrClOS$) S30

Biological Data

List of Figures

Figure S19. Biological Data for Compound **4** (a) anti-proliferation against HREC, (b) anti-proliferation against ARPE-19. S31

Figure S20. Biological Data for Compound **5** (a) anti-proliferation against HREC, (b) anti-proliferation against ARPE-19. S32

Figure S21. Biological Data for Compound **6** (a) anti-proliferation against HREC, (b) anti-proliferation against ARPE-19. S33

Figure S22. Biological Data for Compound **7** (a) anti-proliferation against HREC, (b) anti-proliferation against ARPE-19. S34

Figure S23. Biological Data for Compound **8** (a) anti-proliferation against HREC, (b) anti-proliferation against ARPE-19. S35

Figure S24. Biological Data for Compound **9** (a) anti-proliferation against HREC, (b) anti-proliferation against ARPE-19. S36

Figure S25. Biological Data for Compound **10** (a) anti-proliferation against HREC, (b) anti-proliferation against ARPE-19. S37

Figure S26. Biological Data for Compound **11** (a) anti-proliferation against HREC, (b) anti-proliferation against ARPE-19. S38

Figure S27. Biological Data for Compound **12** (a) anti-proliferation against HREC, (b) anti-proliferation against ARPE-19. S39

Figure S28. Biological Data for Compound **13** (a) anti-proliferation against HREC, (b) anti-proliferation against ARPE-19. S40

Figure S29. Images of Matrigel experiments for compounds **4**, **5**, **6**, **10**, **11**, **13** S41

Figure S30. HRMS data S44

3-[(4-Chlorophenyl)sulfanyl]propanoic acid (2a)

White solid, 1.41 g, 65.1 %, R_f = 0.68 (1:1 EtOAc:hexane), 93.7 - 93.9 °C, IR ν_{max} (cm^{-1}): 2561 (O-H, carboxylic acid), 1696 (C=O), 1477 (Aromatic C=C), 1092 (C-O), 813 (C-Cl), 659 (C-S),^{1}H-NMR (400 MHz, $CDCl_3$): δ = 7.24 (2H, d, J = 8.9 Hz, 2 x H-5), 7.20 (2H, d, J = 8.9 Hz, 2 x H-6), 3.07 (2H, t, J = 7.2 Hz, H-2), 2.59 (2H, t, J = 7.2 Hz, H-3); ^{13}C-NMR (100 MHz, $CDCl_3$): δ =177.3 (C-4), 133.0 (C-8), 132.9 (C-7), 131.8 (C-5), 129.3 (C-6), 34.0 (C-3), 29.1(C-2). Purity 98.3% (by NMR)

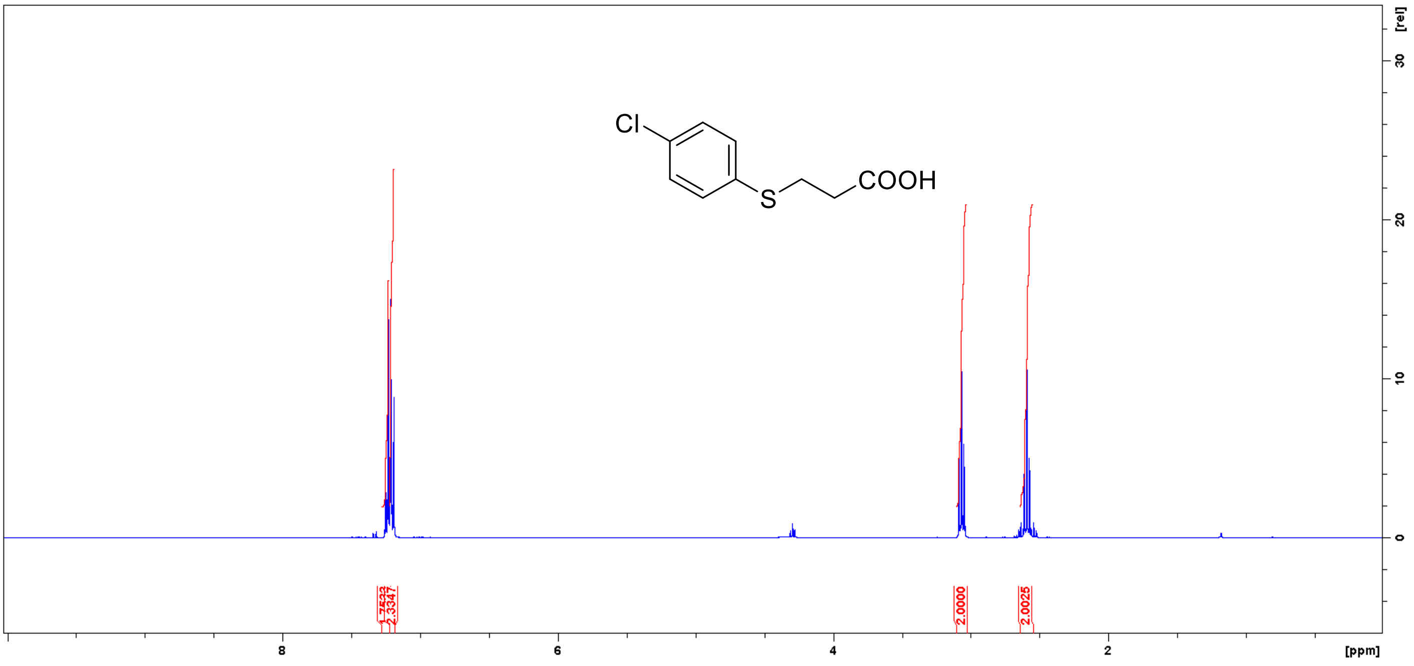

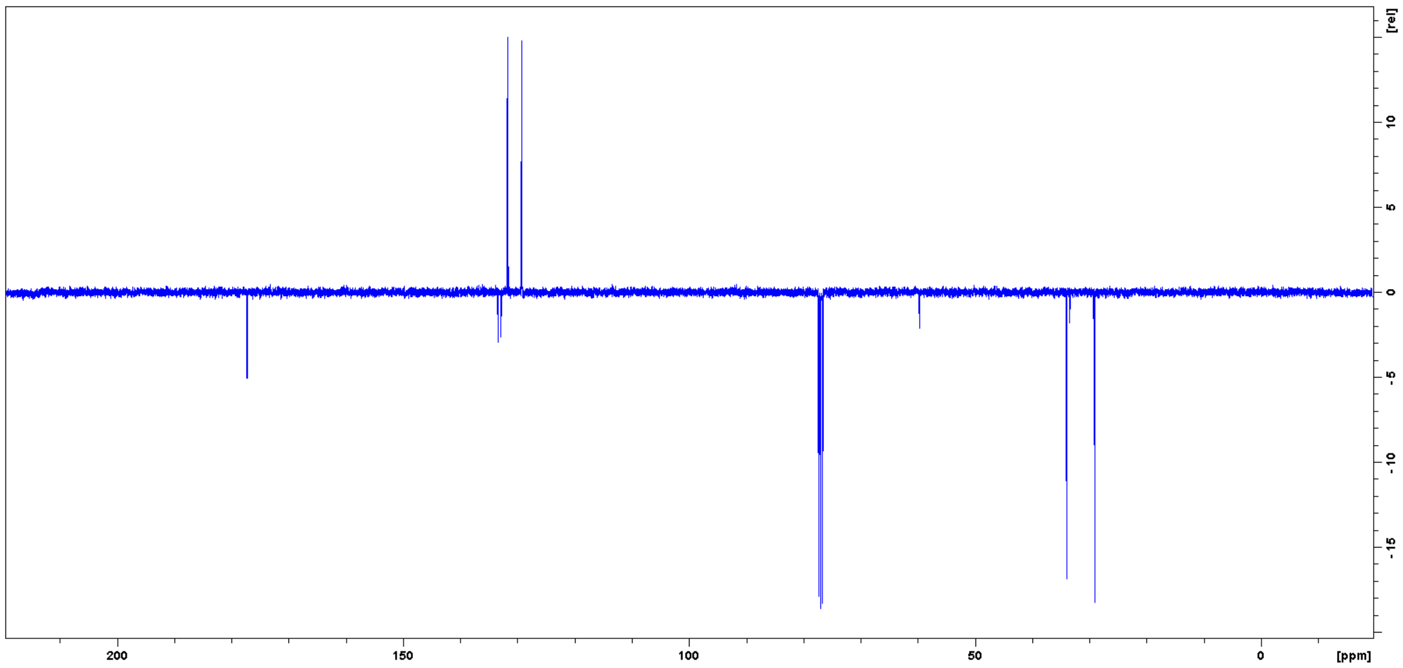

Figure S1. ^{1}H-NMR and ^{13}C-NMR spectra of compound **2a**

3-[(4-Bromophenyl)sulfanyl]propanoic acid (2b)

White solid, 2.41 g, 91.9 %, R_f = 0.70 (1:1 EtOAc:hexane), 120.3 - 121.0 °C, IR ν_{max} (cm^{-1}): 2561 (O-H), 1697 (C=O), 1474 (Aromatic C=C), 1006 (C-O), 809 (C-Br), 657 (C-S), ^{1}H-NMR (400 MHz, $CDCl_3$): δ = 7.40 (2H, d, J = 8.6 Hz, 2 x H-6), 7.20 (2H, d, J = 8.6 Hz, 2 x H-5), 3.70 (2H, t, J = 7.2 Hz, H-2), 2.60 (2H, t, J = 7.2 Hz, H-3); ^{13}C-NMR (100 MHz, $CDCl_3$): δ = 177.2 (C-4), 134.1 (C-8) 132.2 (C-5), 131.8 (C-6), 120.8 (C-7), 33.9 (C-3), 28.9 (C-2).

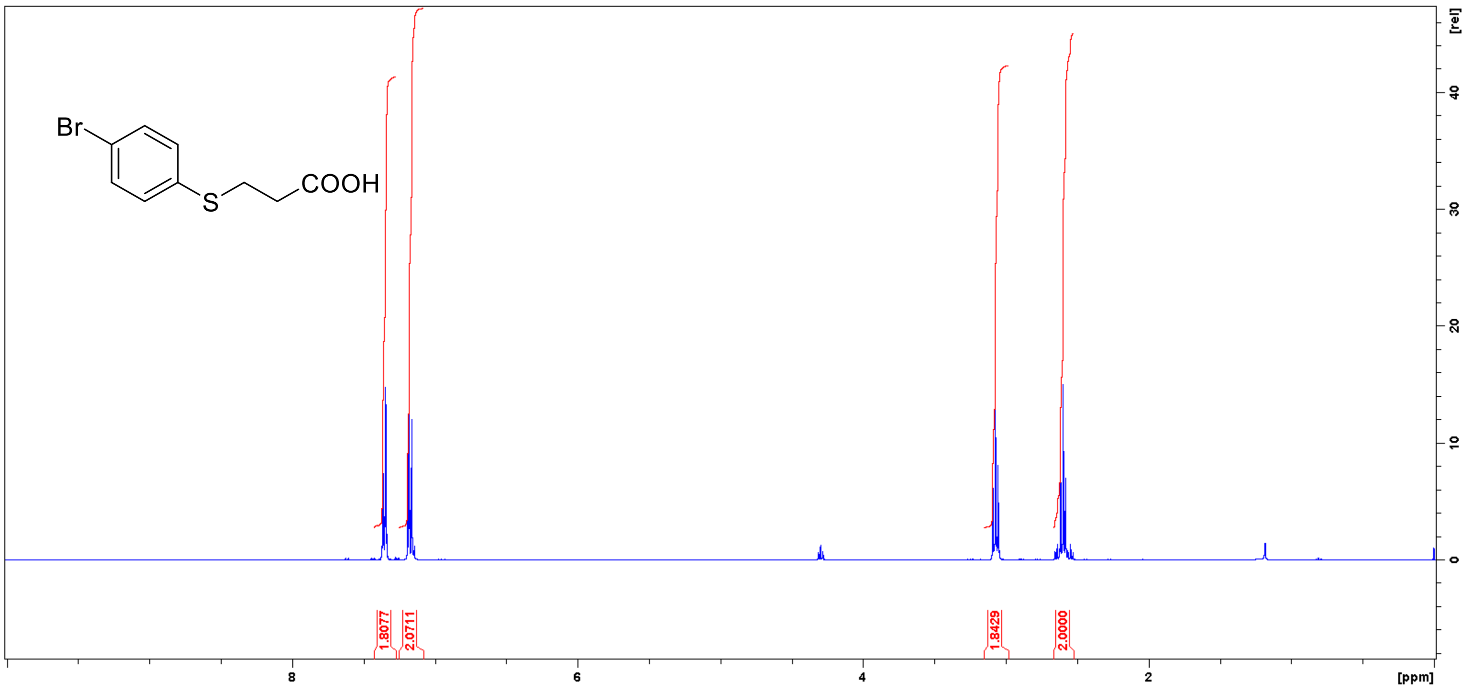

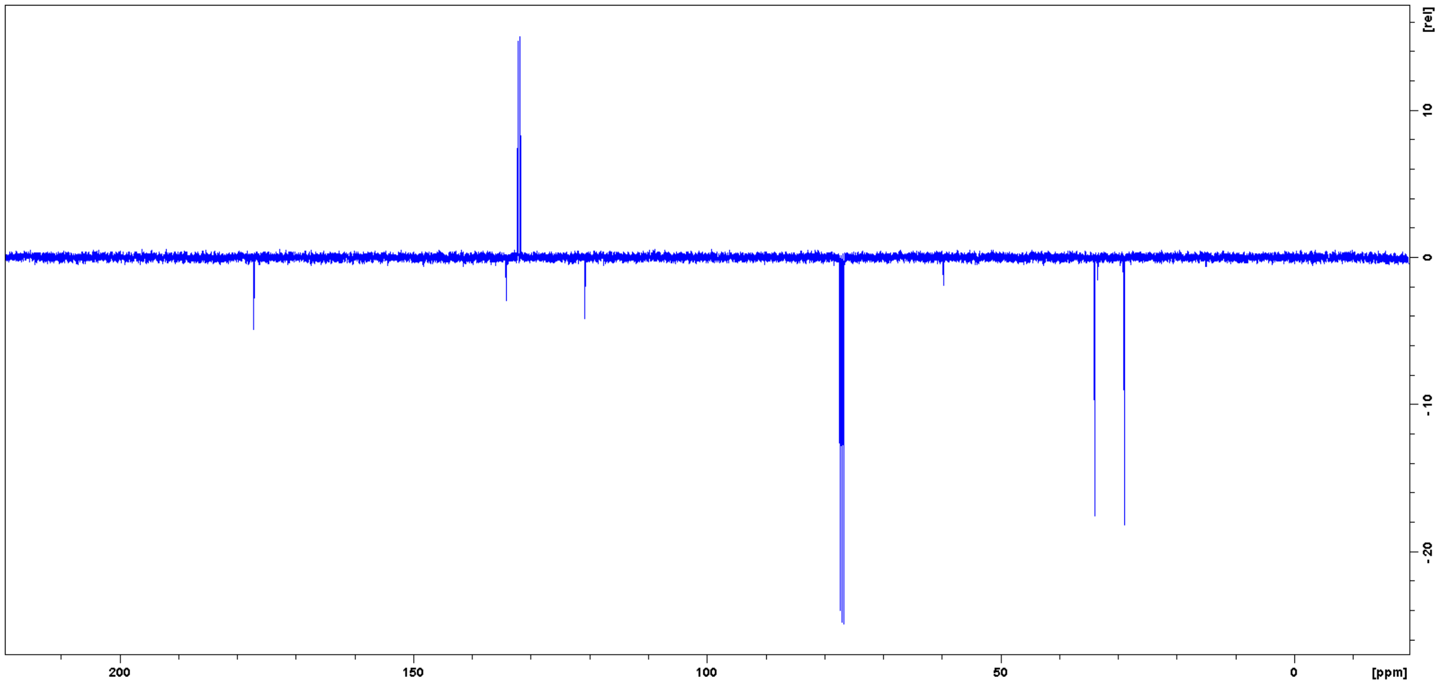

Figure S2. ^{1}H-NMR and ^{13}C-NMR spectra of compound **2b**

3-[(4-Methoxyphenyl)sulfanyl]propanoic acid (2c)

White solid, 2.08 g, 97.4 %, R_f = 0.73 (1:1 EtOAc:hexane), 85.2 - 85.7 °C, IR ν_{max} (cm^{-1}): 2958 (Ar-H), 2591 (O-H), 1694 (C=O), 1492 (CH_3 bend), 1469 (Aromatic C=C), 1027 (C-O), 664 (C-S), ^{1}H-NMR (400 MHz, $CDCl_3$): δ = 7.32 (2H, d, J = 8.7 Hz, 2 x H-6), 6.78 (2H, d, J = 8.7, 2 x H-5), 3.73 (3H, s, OCH_3), 2.96 (2H, t, J = 7.0 H, H-2), 2.53 (2H, t, J = 7.0, H-3); ^{13}C-NMR (100 MHz, $CDCl_3$): δ = 177.7 (C-4), 159.5 (C-7), 134.5 (C-5), 124.8 (C-8), 114.7 (C-6), 55.4 (OCH_3), 34.3 (C-3), 30.8 (C-2).

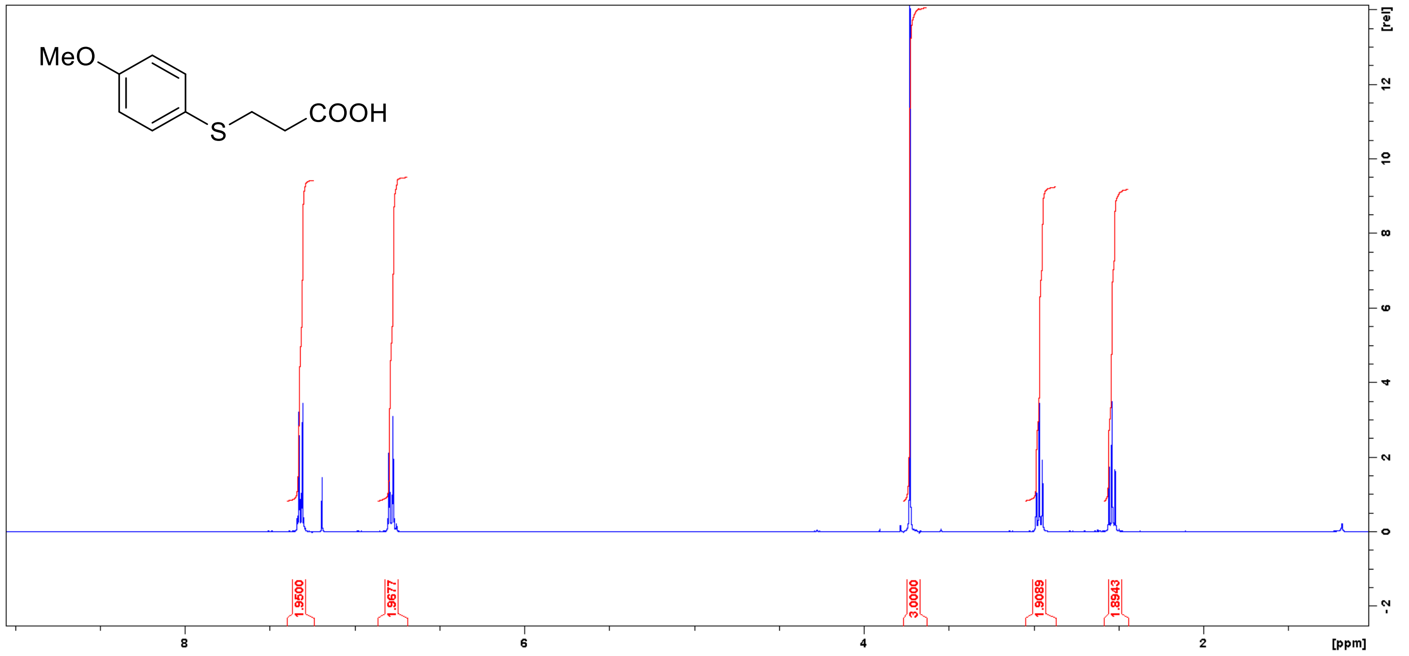

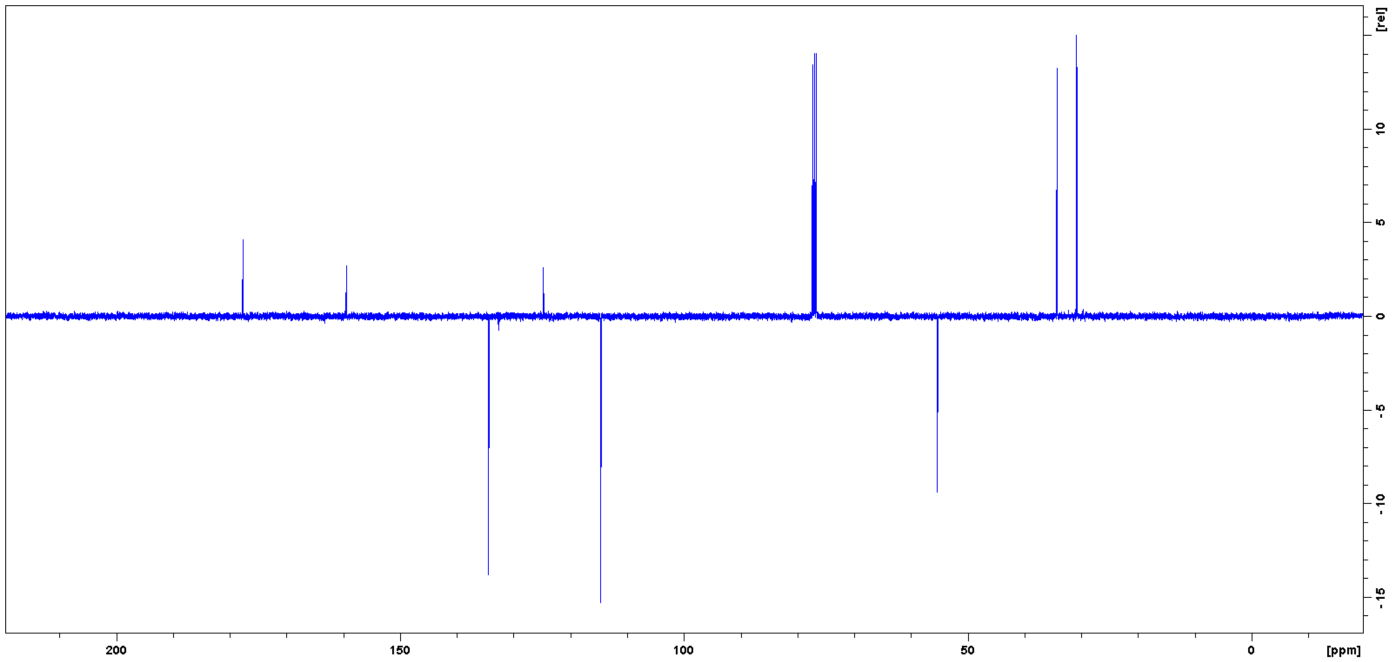

Figure S3. ^{1}H-NMR and ^{13}C-NMR spectra of compound **2c**

6-Chloro-2,3-dihydro-4H-1-benzothiopyran-4-one (3a)

Light orange solid, 0.46 g, 50.3 %, R_f = 0.49 (1:1 EtOAc:hexane), 78.8 - 79.3 °C, IR ν_{max} (cm^{-1}): 3047 (Ar-H), 2939 (Aliphatic C-H), 1666 (C=O), 1472 (Aromatic C=C), 815 (C-Cl), 655 (C-S),^{1}H-NMR (400 MHz, $CDCl_3$): δ = 8.00 (1H, d, J = 2.5 Hz, H-5), 7.26 (1H, dd, J = 2.5 Hz, 8.6 Hz, H-7), 7.15 (1H, d, J = 8.6 Hz, H-8), 3.17 (2H, m, H-2), 2.90 (2H, m, H-3); ^{13}C-NMR (100 MHz, $CDCl_3$): δ = 192.9 (C-4), 140.5 (C-8a), 133.4 (C-7), 131.9 (C-4a), 131.2 (C-6), 129.1 (C-8), 128.8 (C-5), 39.3 (C3), 26.9 (C-2).

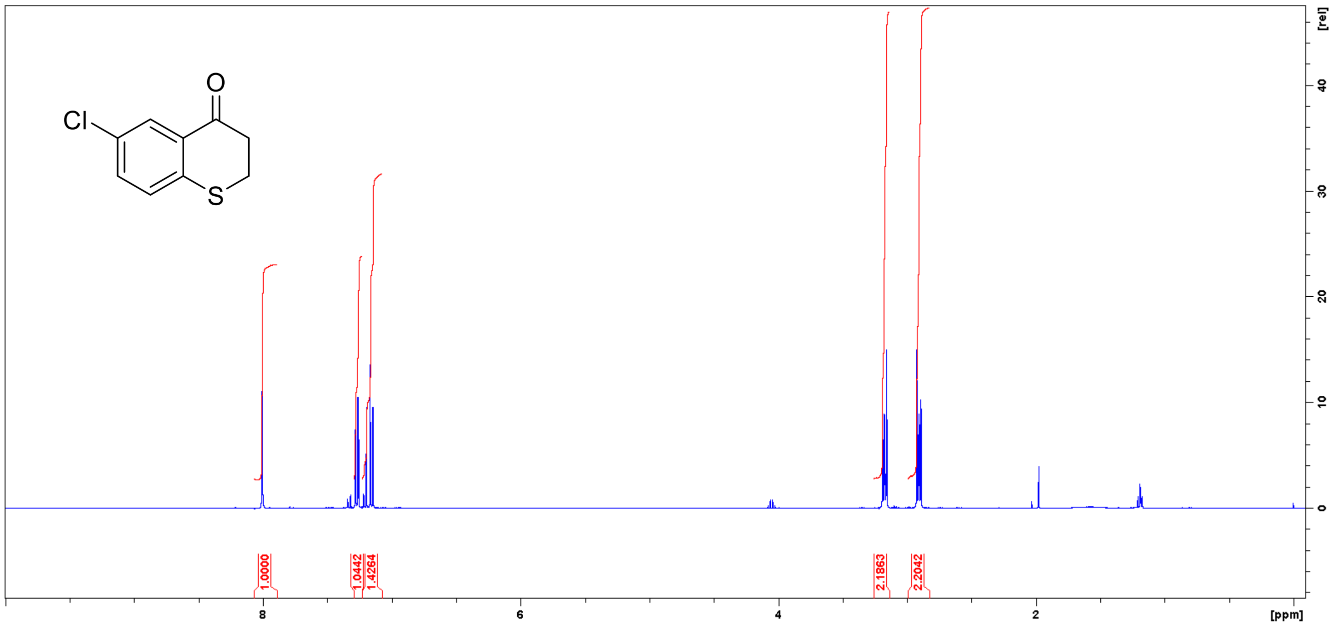

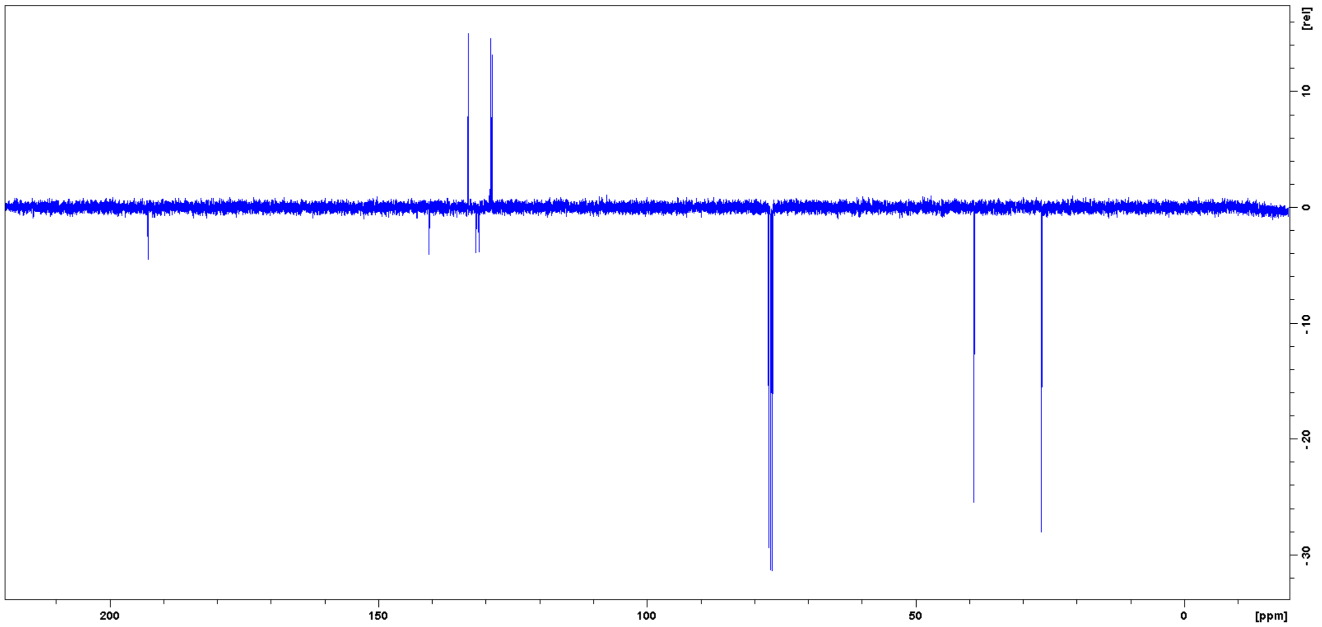

Figure S4. ^{1}H-NMR and ^{13}C-NMR spectra of compound **3a**

6-Chloro-2,3-dihydro-4H-1-benzothiopyran-4-one (3b)

Light orange solid, 0.47 g, 49.7 %, R_f = 0.46 (1:1 EtOAc:hexane), 120.3 - 121.0 °C, IR ν_{max} (cm^{-1}): 2930 (Ar-H), 1667 (C=O), 1447 (Aromatic C=C), 809 (C-Br), 657 (C-S), ^{1}H-NMR (400 MHz, $CDCl_3$): δ = 8.24 (1H, d, J = 2.1 Hz, H-5), 7.50 (1H, dd, J = 2.1 Hz, 7.7 Hz, H-7), 7.18(1H, d, J = 8.5 Hz, H-8), 3.26 (2H, m, H-2), 2.99 (2H, m, H-3); ^{13}C-NMR (100 MHz, $CDCl_3$): δ = 192.9 (C-4), 141.2 (C-4a), 136.1 (C-7), 132.0 (C-8a), 131.5 (C-5), 129.4 (C-8), 119.0 (C-6), 39.1 (C-3), 34.4 (C-2).

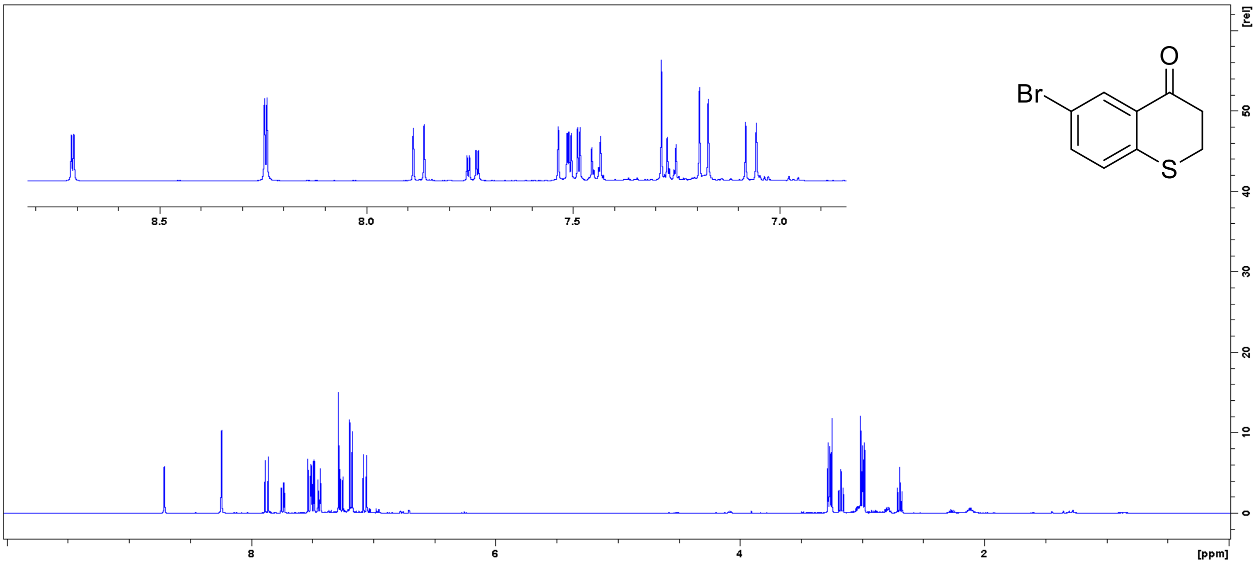

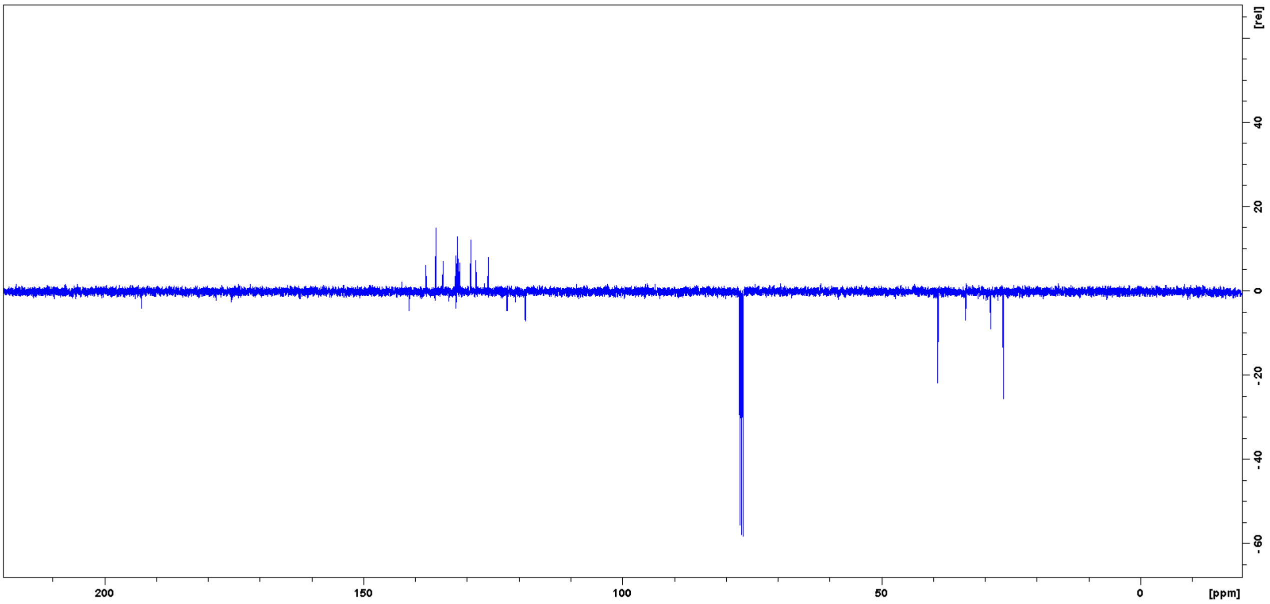

Figure S5. ^{1}H-NMR and ^{13}C-NMR spectra of compound **3b**

6-Methoxy-2,3-dihydro-4H-1-benzothiopyran-4-one (3c)

Light orange solid, 0.35 g, R_f = 0.43 (1:1 EtOAc:hexane), 38.6 %, 85.2 - 85.7 °C, IR ν_{max} (cm^{-1}): 3022 (Ar-H), 1598 (C=O), 1473 (Aromatic C=C), 1359 (CH_3 bend), 1046 (C-O), 637 (C-S), ^{1}H-NMR (400 MHz, $CDCl_3$): δ = 7.53 (1H, d, J = 3.0 Hz, H-5), 7.09 (1H, d, J = 8.8 Hz, H-8), 6.92 (1H, dd, J = 3.0 Hz, 8.8 Hz, H-7), 3.74 (3H, s, OCH_3), 3.12 (2H, m, H-2), 2.88 (2H, m, H-3); ^{13}C-NMR (100 MHz, $CDCl_3$): δ = 194.2 (C-4), 157.5 (C-6), 133.6 (C-8), 131.6 (C-4a), 129.6 (C-8), 122.1 (C-7), 111.6 (C-5), 55.5 (OCH_3), 40.7 (C-3), 27.6 (C-2).

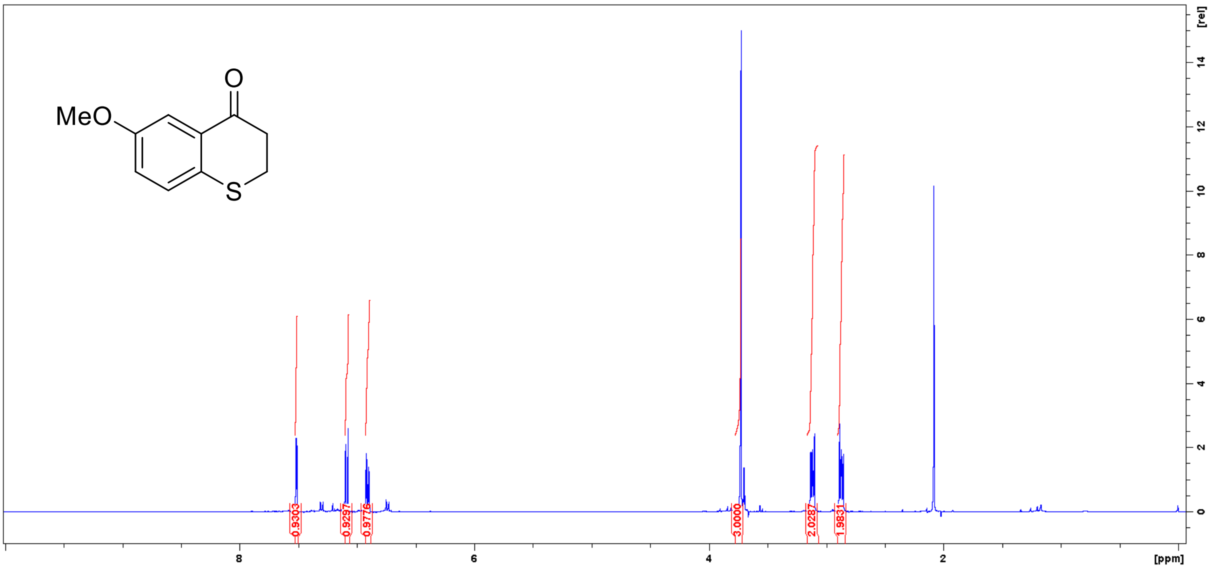

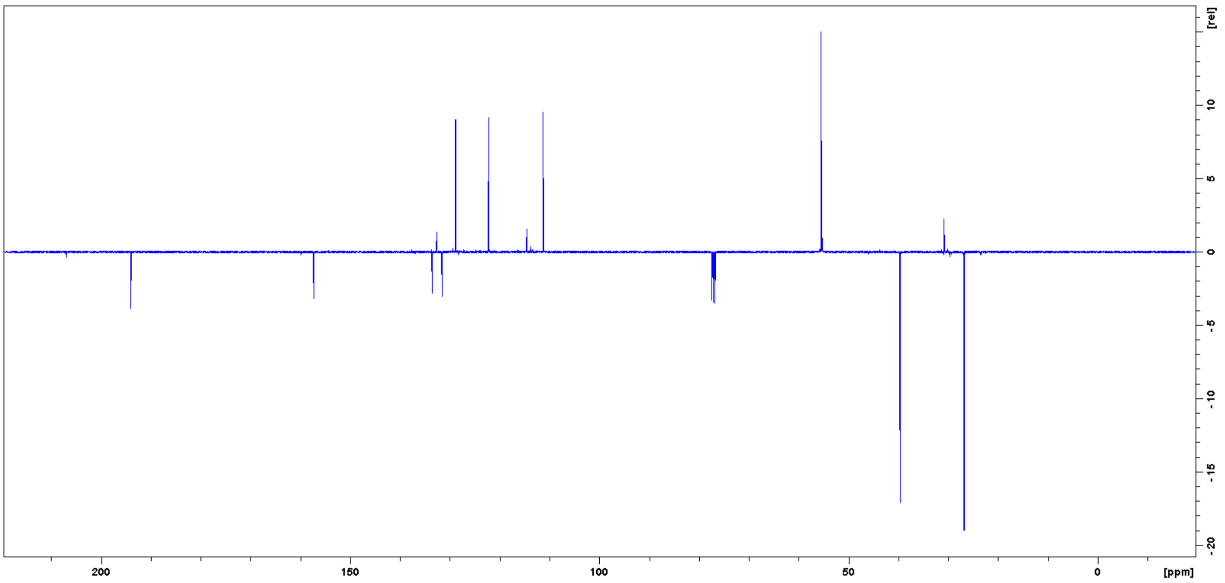

Figure S6. ^{1}H-NMR and ^{13}C-NMR spectra of compound **3c**

(3*Z*)-6-Bromo-3-[(4-bromophenyl)methylidene]-2,3-dihydro-4H-1-benzothiopyran-4-one (4)

Light yellow solid, 244 mg, 28.3 %, R_f = 0.75 (1:1 EtOAc:hexane), 186.2 - 186.7 °C, IR ν_{max} (cm^{-1}): 1647 (C=O), 1568 (Aliphatic C=C), 1485 (Aromatic C=C), 812 (C-Br), 664 (C-S), ^{1}H-NMR (400 MHz, $CDCl_3$): δ = 8.23 (1H, d, J = 2.2 Hz, H-5), 7.62 (1H, brs, $w_{1/2}$ = 2.65 Hz, H-9), 7.51 (2H, d, J = 8.4 Hz, H-3'), 7.44 (1H, dd, J = 2.2 Hz, 8.2 Hz, H-7), 7.19 (2H, d, 8.4 Hz, H-2'), 7.12 (1H, d, J = 8.2 Hz, H-8), 3.99 (2H, d, J - 1.0 Hz, H-2); ^{13}C-NMR (100 MHz, $CDCl_3$): δ = 184.5 (C-4), 133.9 (C-8a), 137.2 (C-9), 136.0 (C-7), 133.6 (C-1'), 133.5 (C-4a), 133.3 (C-5), 132.5 (C-3), 132.2 (C-3'), 131.0 (C-2'), 129.5 (C-8), 123.5 (C-4'), 119.7 (C-6), 29.1 (C-2), HRESMS (ASAP) *m/z* 408.8895 $[M+H]^+$ (calcd for $[C_{16}H_{11}OSBr_2]$, 408.8897). Purity 98.3% (by NMR)

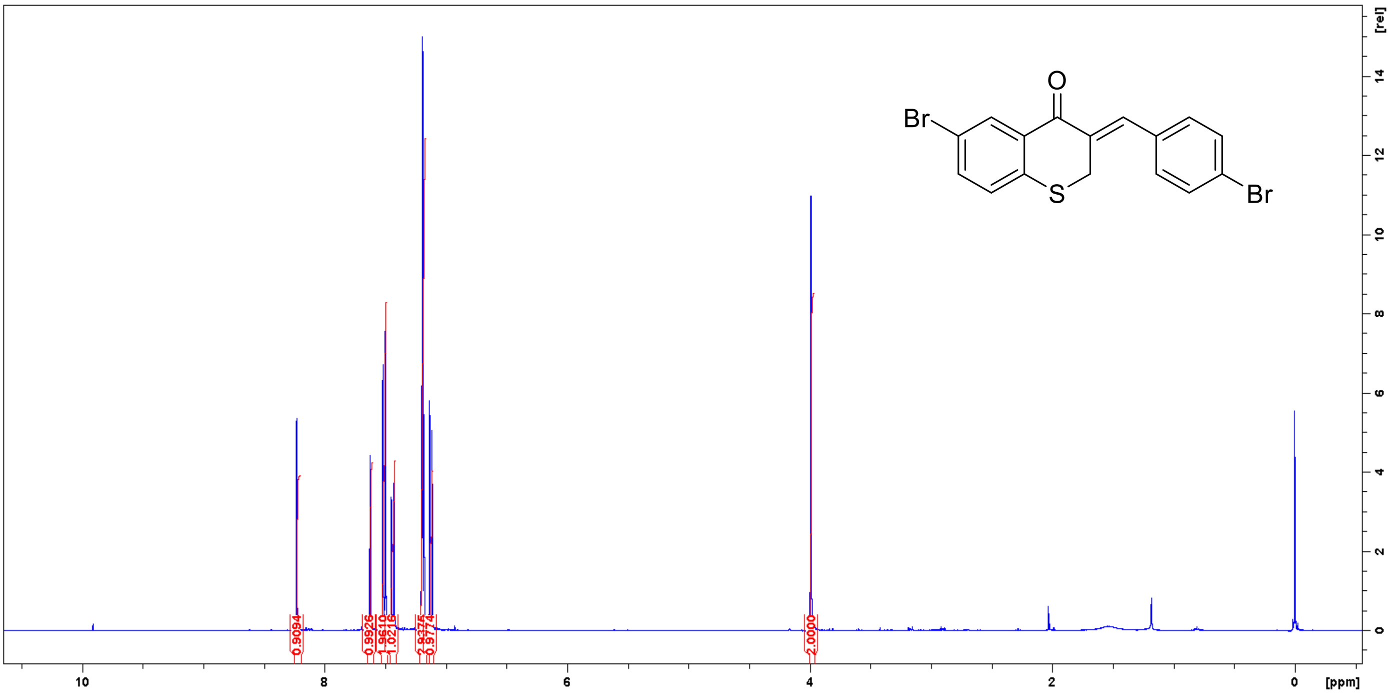

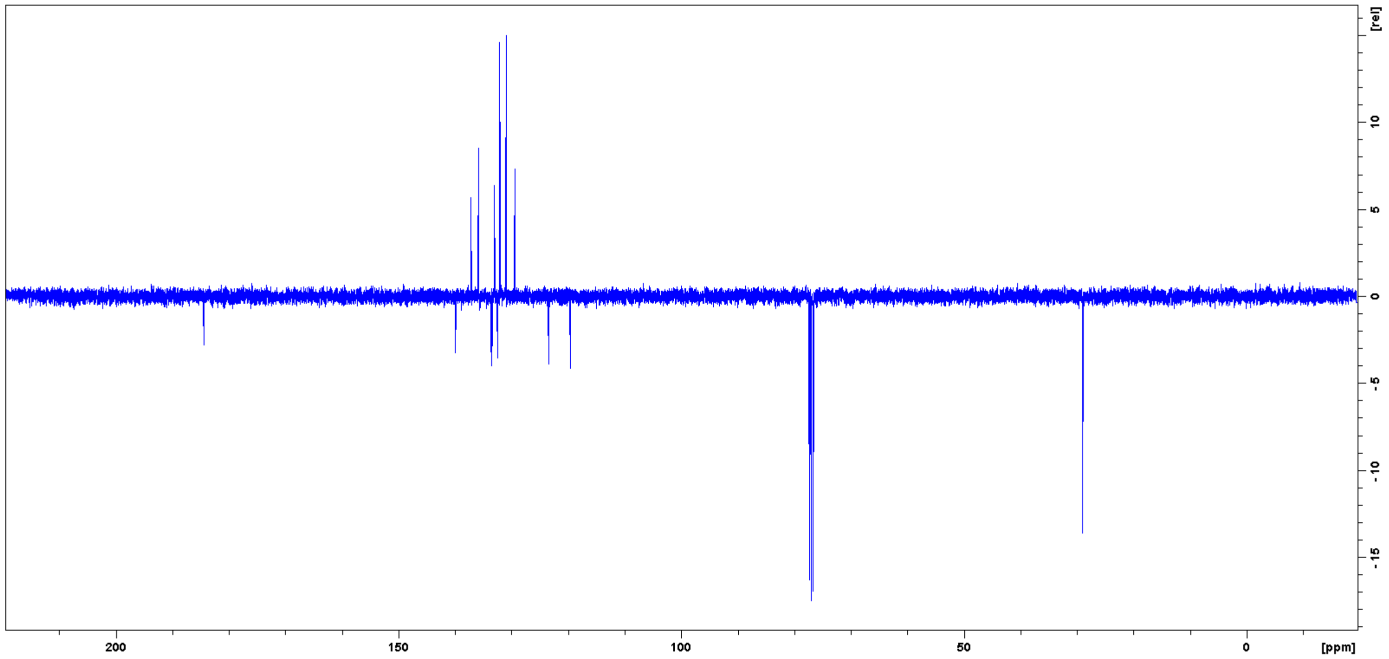

Figure S7. ^{1}H-NMR and ^{13}C-NMR spectra of compound **4**

(3*Z*)-3-[(4-Bromophenyl)methylidene]-6-chloro-2,3-dihydro-4H-1-benzothiopyran-4-one (5)

Light yellow solid, 742 mg, 92.3 %, R_f = 0.78 (1:1 EtOAc:hexane),171.2 - 171.8°C, IR ν_{max} (cm^{-1}): 3375 (Ar-H), 1659 (C=O), 1595 (Aliphatic C=C), 1487 (Aromatic C=C), 814 (C-Cl), 803 (C-Br), 665 (C-S). ^{1}H-NMR (400 MHz, $CDCl_3$): δ = 8.08 (1H, d, J = 2.3 Hz, H-5), 7.62 (1H, brs, $w_{1/2}$ = 3.2 Hz, H-9), 7.51 (1H, d, J = 7.9 Hz, H-3'), 7.29 (1H, dd, J = 2.3, 8.2 Hz, H-7), 7.19 (1H, d, J = 7.9 Hz, H-2'), 7.19 (1H, d, J = 8.2 Hz, H-8), 3.97 (2H, d, J = 1.3 Hz, H-2); ^{13}C-NMR (100 MHz, $CDCl_3$): δ = 184.6 (C-4), 139.3 (C-8a), 137.2 (C-9), 133.6 (C-6), 133.1 (C-7), 132.6 (C-3), 132.3 (C-4'), 132.2 (C-4a), 132.1 (C-3'), 131.0 (C-2'), 130.0 (C-5), 129.4 (C-8), 29.1 (C-2), HRESMS (ASAP) *m/z* 364.9399 $[M+H]^+$ (calcd [$C_{16}H_{11}OSClBr$], 364.9043). CCDC Deposition Number: 2421737. Purity 99.2% (by NMR)

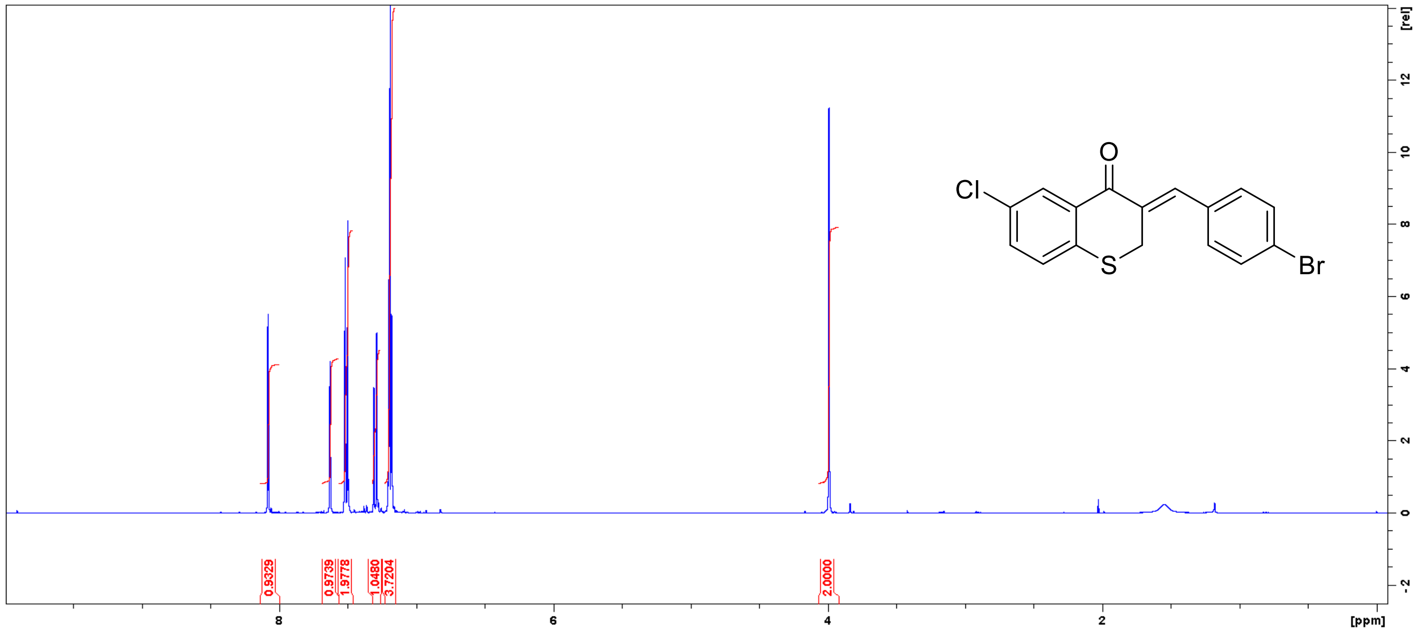

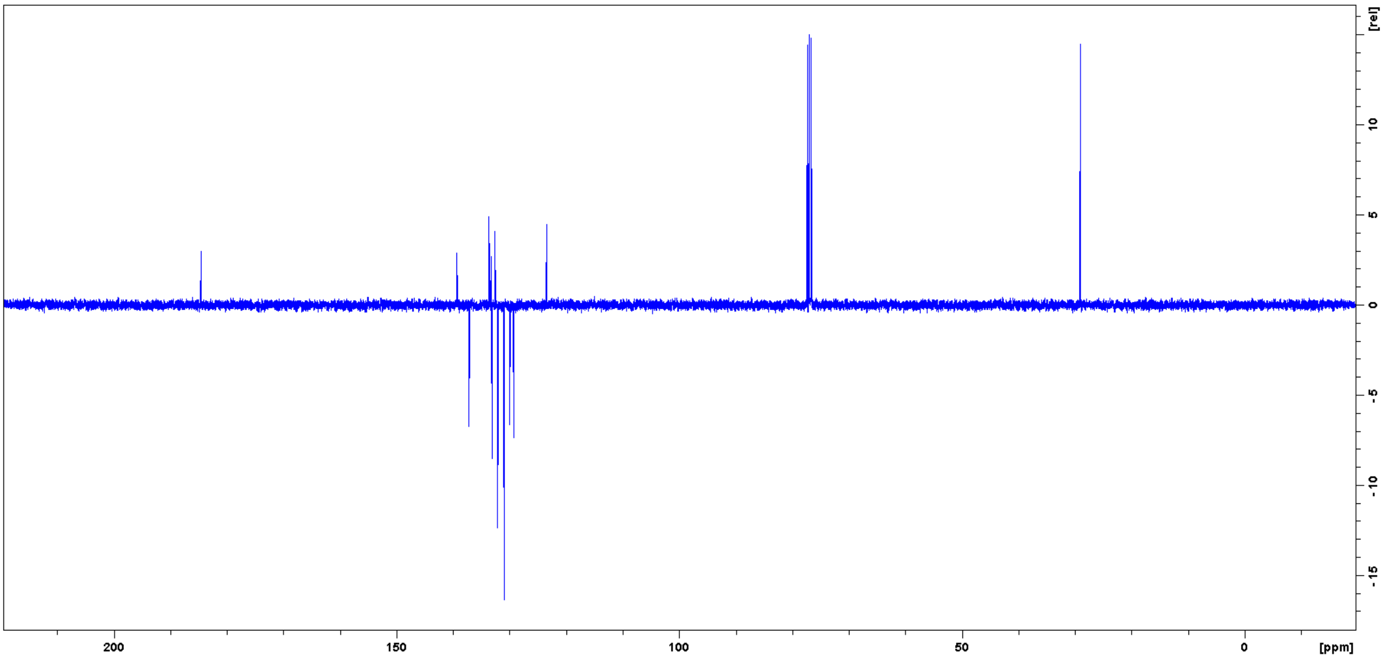

Figure S8. ^{1}H-NMR and ^{13}C-NMR spectra of compound **5**

(3*Z*)-3-[(4-Bromophenyl)methylidene]-6-methoxy-2,3-dihydro-4H-1-benzothiopyran-4-one (6)

Light yellow solid, 260 mg, 44.4 %, R_f = 0.75 (1:1 EtOAc:hexane), 146.5 - 147.1 °C, IR ν_{max} (cm^{-1}): 2934 (Ar-H), 1650 (C=O), 1583 (Aliphatic C=C), 1471 (Aromatic C=C), 1026 (C-O), 824 (C-Br), 654 (C-S), ^{1}H-NMR (400 MHz, $CDCl_3$): δ = 7.62 (1H, d, J = 3.0 Hz, H-5), 7.60 (1H, brs, $w_{1/2}$ = 2.70 Hz, H-9), 7.49 (2H, d, J = 8.2 Hz, H-3'), 7.19 (2H, d, J = 8.2 Hz, H-2'), 7.14 (1H, d, J = 8.8 Hz, H-8), 6.95 (1H, dd, J = 3.0 Hz, 8.8 Hz, H-7), 3.96 (2H, d, J = 1.0 Hz, H-2), 3.79 (3H, s, OCH_3); ^{13}C-NMR (100 MHz, $CDCl_3$): δ = 185.6 (C-4), 158.1 (C-6), 136.3 (C-9), 133.9 (C-4'), 133.7 (C-4a), 133.1 (C-8a), 132.4 (C-3), 132.0 (C-3'), 131.3 (C-8a), 129.1 (C-8), 123.2 (C-1'), 122.2 (C-7), 112.8 (C-5), 55.9 (OCH_3), 29.4 (C-2), HRESMS (ASAP) *m/z* 360.9896 $[M+H]^+$ (calcd [$C_{17}H_{14}O_2SBr$], 360.9898). Purity 95.5% (by NMR)

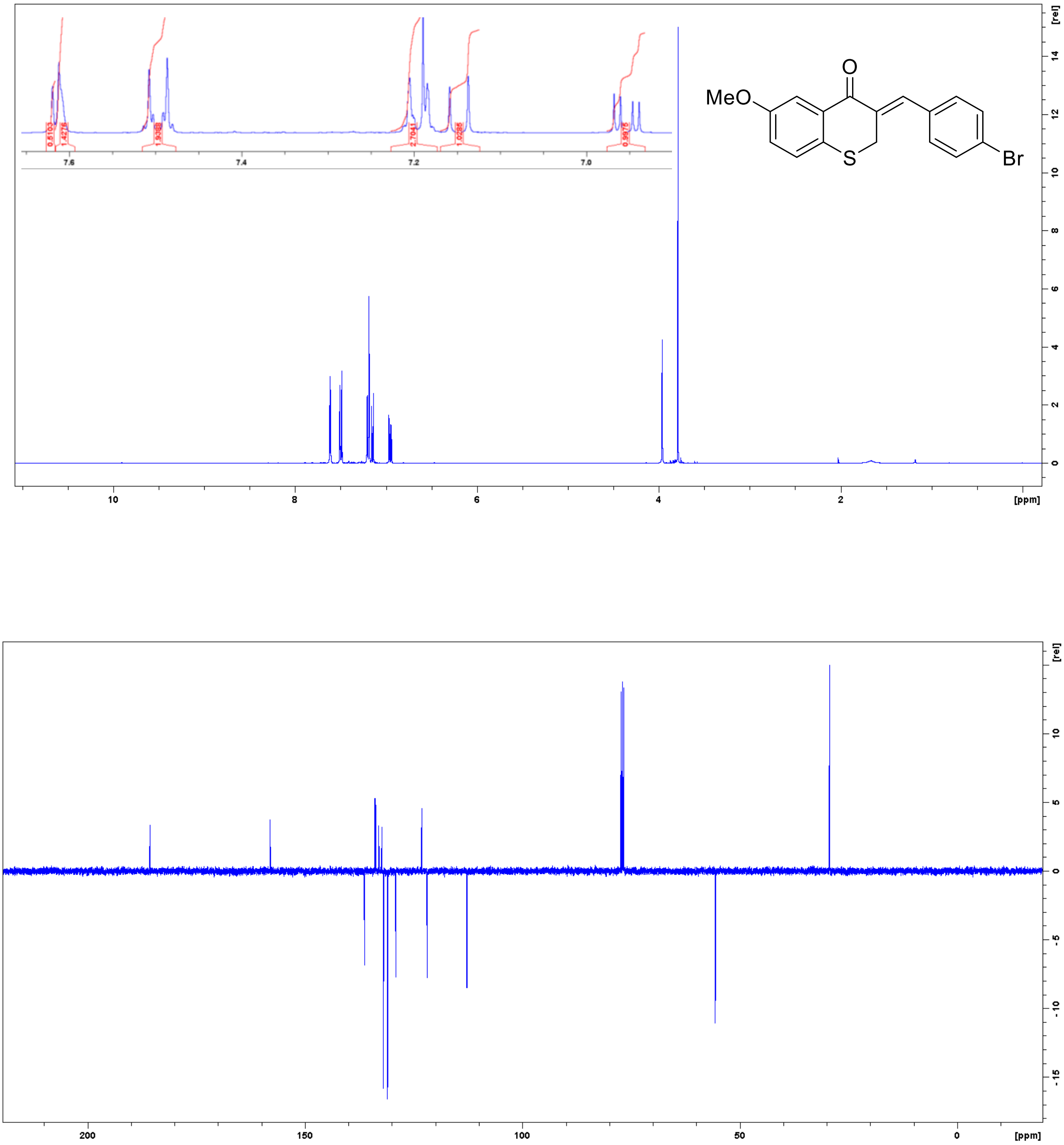

Figure S9. ^{1}H-NMR and ^{13}C-NMR spectra of compound **6**

(3*Z*)-6-Bromo-3-[(3-bromophenyl)methylidene]-2,3-dihydro-4H-1-benzothiopyran-4-one 7)

Light yellow solid, 61 mg, 10.3 %, R_f = 0.74 (1:1 EtOAc:hexane), 192.5 - 193.0 °C, IR ν_{max} (cm^{-1}): 2907 (Ar-H), 1662 (C=O), 1600 (Aliphatic C=C), 1571 (Aromatic C=C), 805 (C-Br), 646 (C-S). ^{1}H-NMR (400 MHz, $CDCl_3$): δ = 8.23 (1H, d, J =2.1 Hz, H-5), 7.62 (1H, brs, w1/2 = 2.7 Hz, H-9), 7.45 (1H, d, J = 1.8 Hz, H-2’), 7.45 (1H, m, H-6’), 7.44 (1H, dd, J = 2.1, 8.6 Hz, H-7), 7.25 (1H, m, *H-4’), 7.25 (1H, m, *H-5’), 7.13 (1H, d, J = 8.6 Hz, H-8), 3.99 (2H, d, J = 1.2 Hz, H-2); ^{13}C-NMR (100 MHz, CDCl3): δ = 184.6 (C-4), 139.8 (C-8a), 136.8 (C-9), 136.4 (C-3), 135.8 (C-5’), 133.6 (C-4a), 133.0 (C-5), 132.2 (C-2’), 132.0 (C-7), 130.1 (C-4’), 129.55 (C-8), 127.9 (C-6’), 122.9 (C-3’), 119.6 (C-6), 28.9 (C-2), HRESMS (ASAP) m/z 408.8896 $[M+H]^+$ (calcd [C16H11OSBr], 408.8897). * denotes interchangeable protons. Purity 96.0% (by NMR)

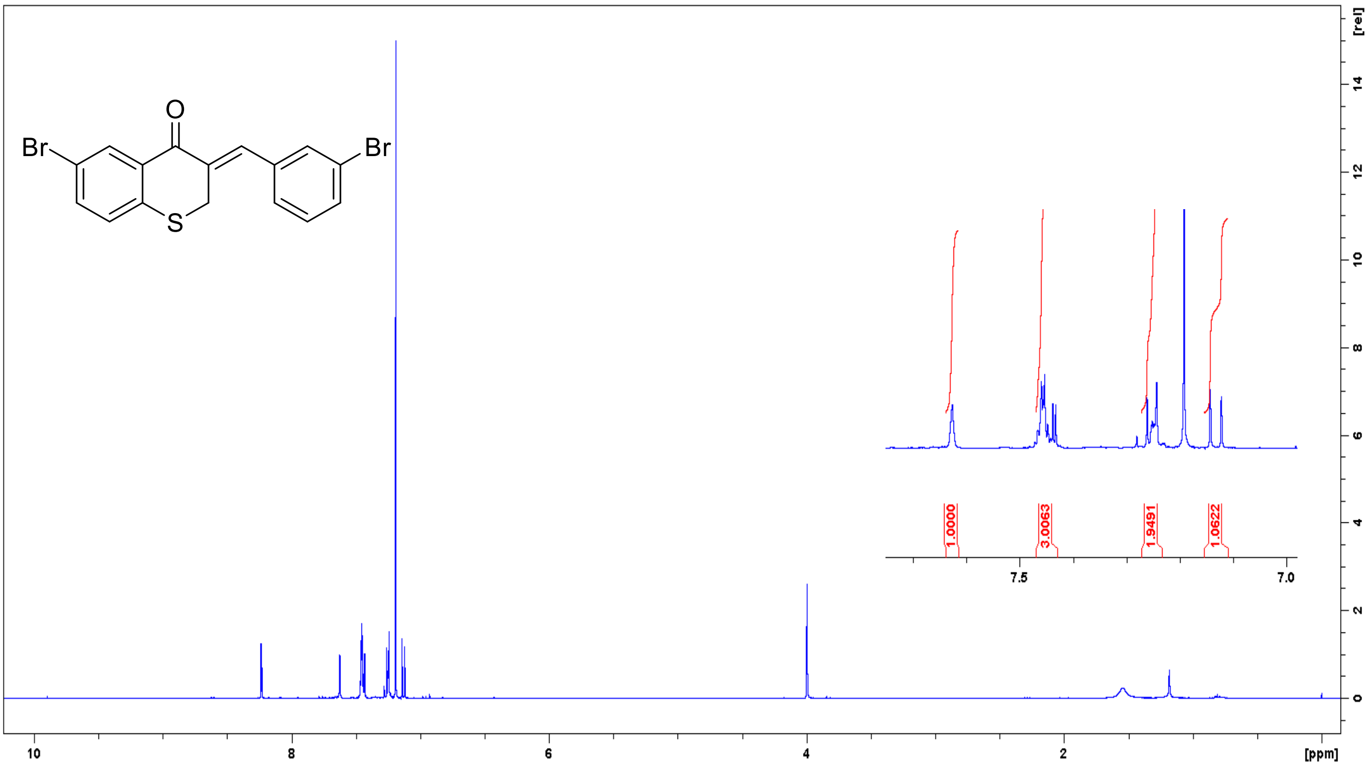

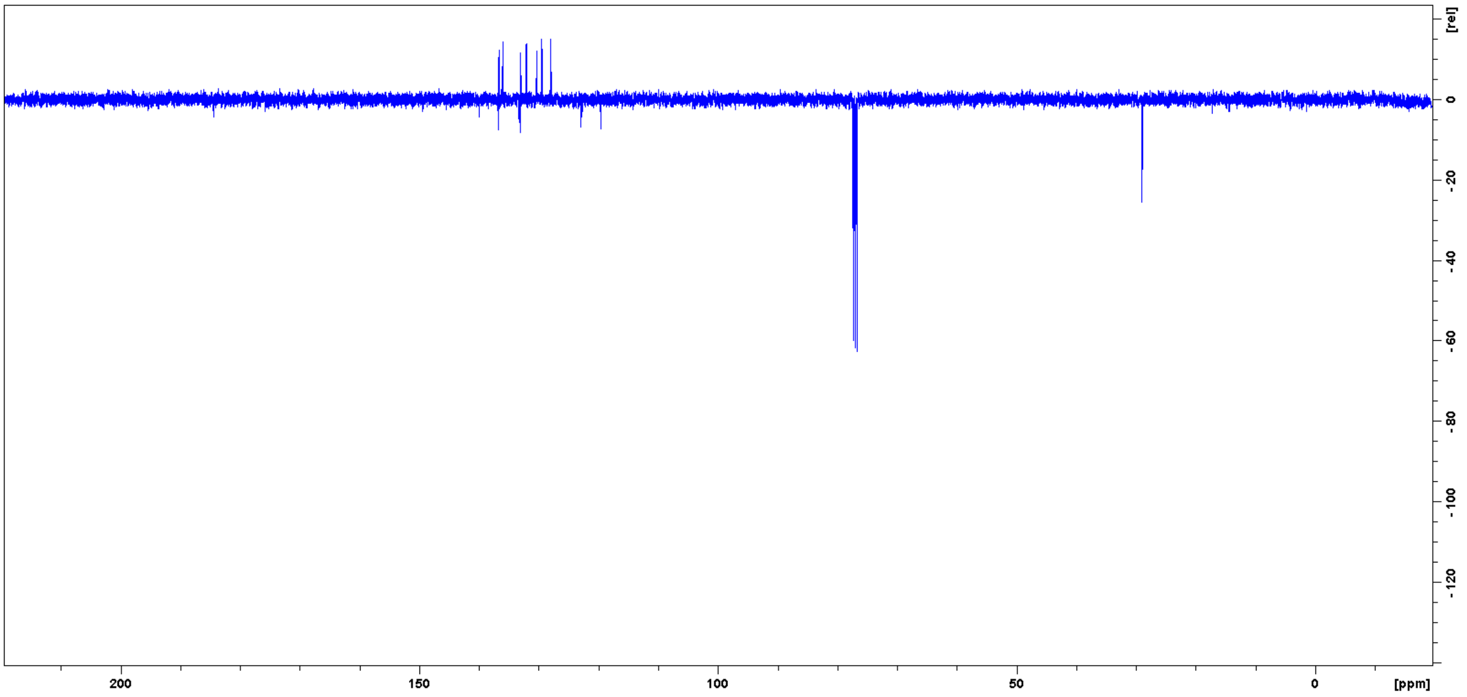

Figure S10. ^{1}H-NMR and ^{13}C-NMR spectra of compound **7**

(3*Z*)-3-[(3-Bromophenyl)methylidene]-6-chloro-2,3-dihydro-4H-1-benzothiopyran-4-one (8)

Light yellow solid, 217 mg, 36.6 %, R_f = 0.74 (1:1 EtOAc:hexane), 169.5 - 169.9 °C, IR ν_{max} (cm^{-1}): 2908 (Ar-H), 1662 (C=O), 1599 (Aliphatic C=C), 1577 (Aromatic C=C), 814 (C-Cl), 782 (C-Br), 646 (C-S). ^{1}H-NMR (400 MHz, $CDCl_3$): δ = 8.07 (1H, d, J = 2.4 Hz, H-5), 7.62 (1H, brs $w_{1/2}$ = 2.5 Hz, H-9), 7.45 (1H, m, H-2'), 7.45 (1H, m, H-6'), 7.29 (1H, dd, J = 2.4, 8.5 Hz, H-7), 7.24 (1H, m, H-4'), 7.24 (1H, m, H-5'), 7.19 (1H, d, J = 8.5 Hz, H-8), 3.99 (2H, d, J = 1.1 Hz, H-2); ^{13}C-NMR (100 MHz, $CDCl_3$): δ = 184.4 (C-4), 139.8 (C-8a), 136.8 (C-1'), 136.7 (C-9), 133.2 (C-4a), 133.2 (C-3), 133.1 (C-7), *132.4 (C-5'), 132.1 (C-6), *132.0 (C-4'), 130.1 (C-5), *130.0 (C-2'), 129.2 (C-8), *127.9 (C-6'), 122.9 (C-3'), 29.8 (C-2), HRESMS (ASAP) *m/z* 364.9400 $[M+H]^+$ (calcd $[C_{16}H_{11}OSClBr]$, 364.9403). * Denotes interchangeable carbons. Purity 90.1% (by NMR)

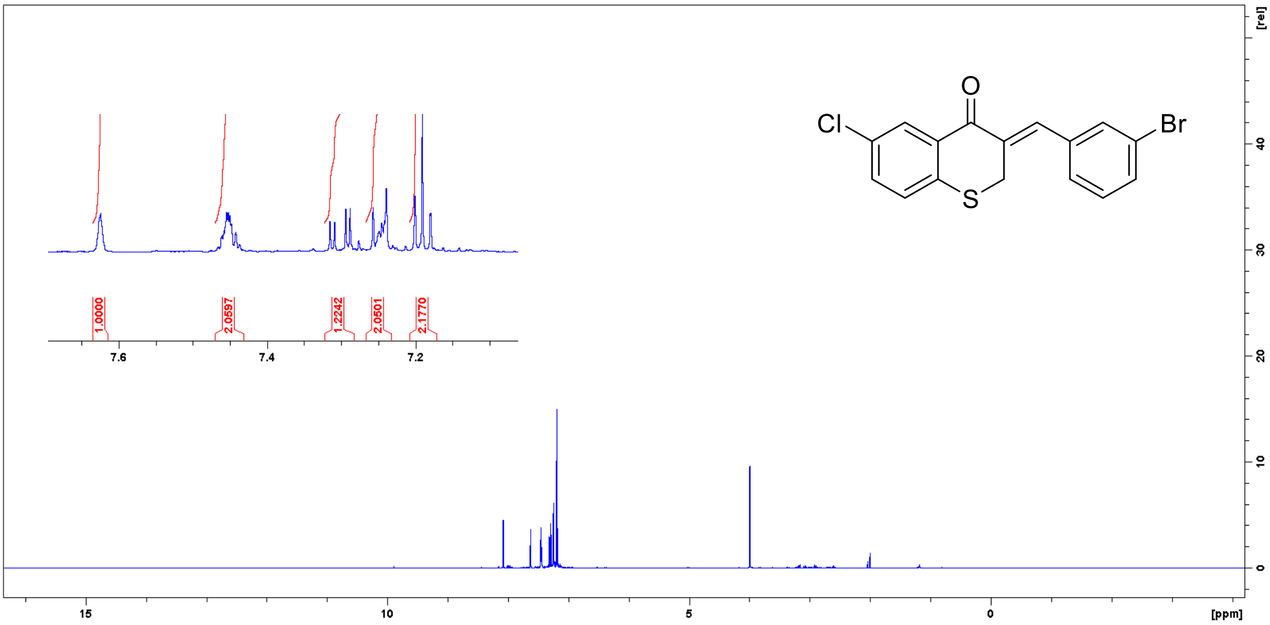

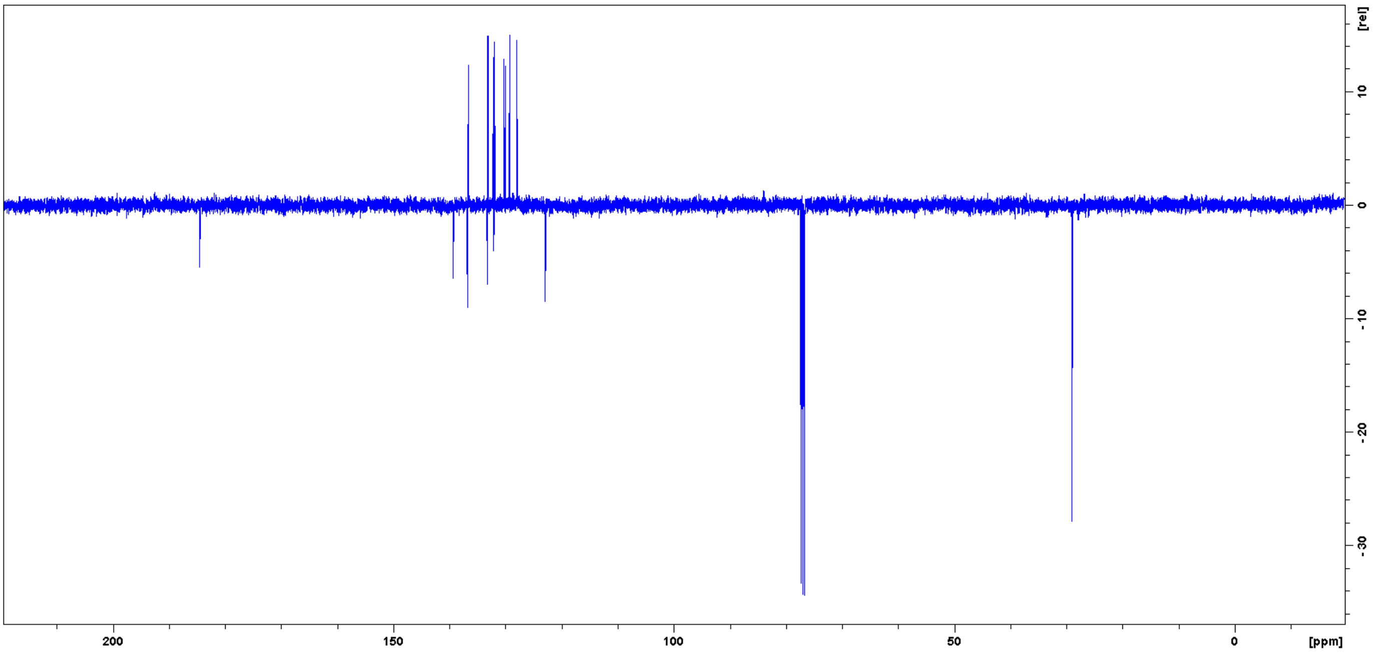

Figure S11. ^{1}H-NMR and ^{13}C-NMR spectra of compound **8**

(3*Z*)-6-Bromo-3-[(4-chlorophenyl)methylidene]-2,3-dihydro-4H-1-benzothiopyran-4-one (9)

Light yellow solid, 692 mg, 13.1 %, R_f = 0.80 (1:1 EtOAc:hexane), 157.8 - 158.0 °C, IR ν_{max} (cm^{-1}): 2909 (Ar-H), 1662 (C=O), 1648 (Aliphatic C=C), 1600 (Aromatic C=C), 815 (C-Cl), 772 (C-Br), 646 (C-S), ^{1}H-NMR (400 MHz, $CDCl_3$): δ = 8.23 (1H, d, J = 2.2 Hz, H-5), 7.65 (1H, brs, $W_{1/2}$ = 2.5 Hz, H-9), 7.44 (1H, dd, J = 2.2 Hz, 8.5 Hz, H-7), 7.35 (2H, s, J = 8.4 Hz, H-2'), 7.25 (2H, d, J = 8.4 Hz, H-3'), 7.13 (1H, d, J = 8.5 Hz, H-8), 3.99 (2H,d, J = 1.1 Hz, H-2); ^{13}C-NMR (100 MHz, $CDCl_3$): δ = 184.3 (C-4), 139.9 (C-8a), 137.2 (C-9), 136.0 (C-7), 135.2 (C-4'), 133.5 (C-4a), 133.1 (C-3), 133.0 (C-5), 132.5 (C-1'), 130.9 (C-3'), 129.5 (C-8), 129.2 (C-2'), 119.6 (C-6), 29.1 (C-2), HRESMS (ASAP) *m/z* 364.9398 $[M+H]^+$ (calcd $[C_{16}H_{11}OSClBr]$, 364.9403). Purity 98.0% (by NMR).

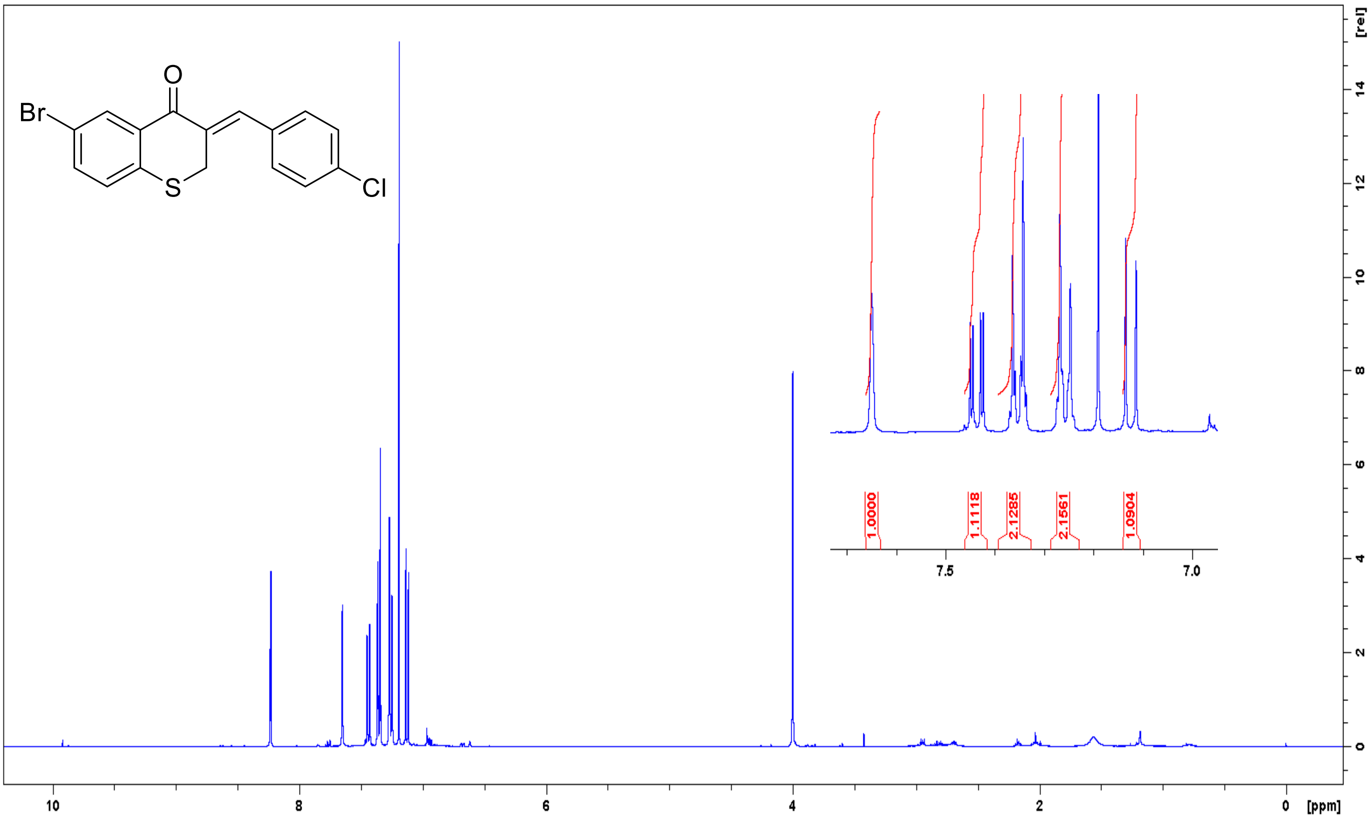

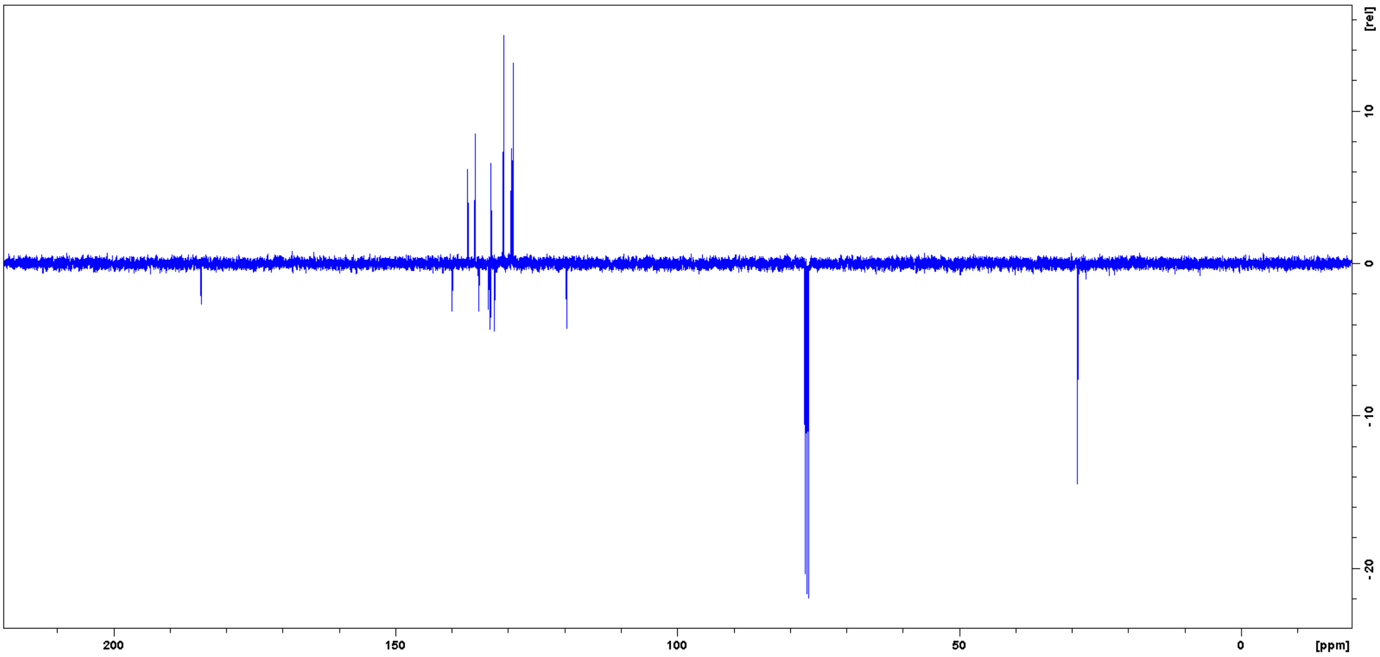

Figure S12. ^{1}H-NMR and ^{13}C-NMR spectra of compound **9**

(3*Z*)-3-[(3-bromophenyl)methylidene]-6-methoxy-2,3-dihydro-4H-1-benzothiopyran-4-one (10)

Yellow solid, 104 mg, 17.8 %, R_f = 0.75 (1:1 EtOAc:hexane), 106.5 - 107.0 °C, IR ν_{max} (cm^{-1}): 2910 (Ar-H), 1660 (C=O), 1592 (Aliphatic C=C), 1558 (Aromatic C=C), 1031 (C-O), 775 (C-Br), 662 (C-S). ^{1}H-NMR (400 MHz, $CDCl_3$): δ = 7.62 (1H, d, J = 2.90 Hz, H-5), 7.61 (1H, s, H-9), 7.45 (1H, m, H-2'), 7.45 (1H, m, H-6'), 7.24 (1H, m, H-4'), 7.24 (1H, m, H-5'), 7.15 (1H, d, J = 8.70 Hz, H-8), 6.96 (1H, dd, J = 2.9, 8.7 Hz, H-7), 3.97 (2H, d, J = 1.1 Hz, H-2), 3.79 (s, OCH_3). ^{13}C-NMR (100 MHz, $CDCl_3$): δ = 185.7 (C-4), 158.4 (C-6), 137.2 (C-1'), 135.2 (C-9), 134.4 (C-3'), 133.0 (C-8a), 132.4 (C-3), 132.1 (C-6'), 131.8 (C-2'), 130.2 (C-4'), 129.5 (C-8), 127.9 (C-5'), 55.6 (OCH_3), 29.3 (C-2). HRESMS (ASAP) *m/z* 360.9897 $[M+H]^+$ (calcd [$C_{17}H_{14}O_2SBr$], 360.9898). Purity 89.3% (by NMR)

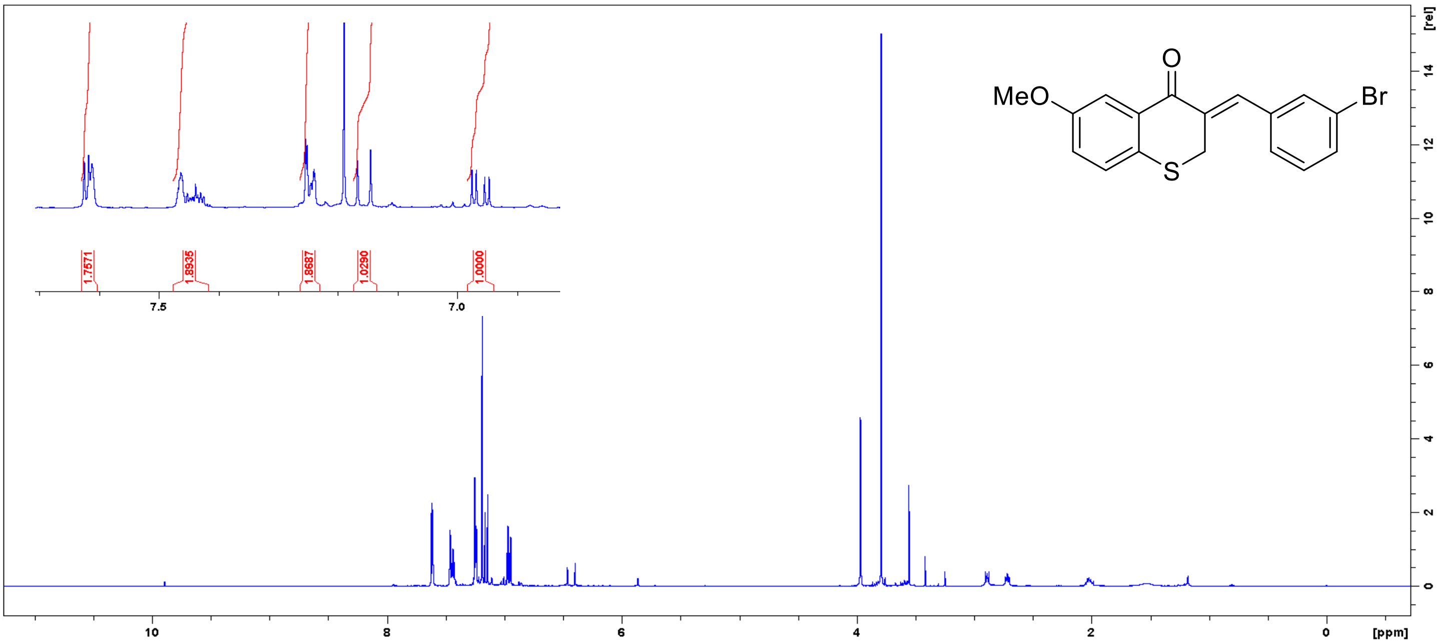

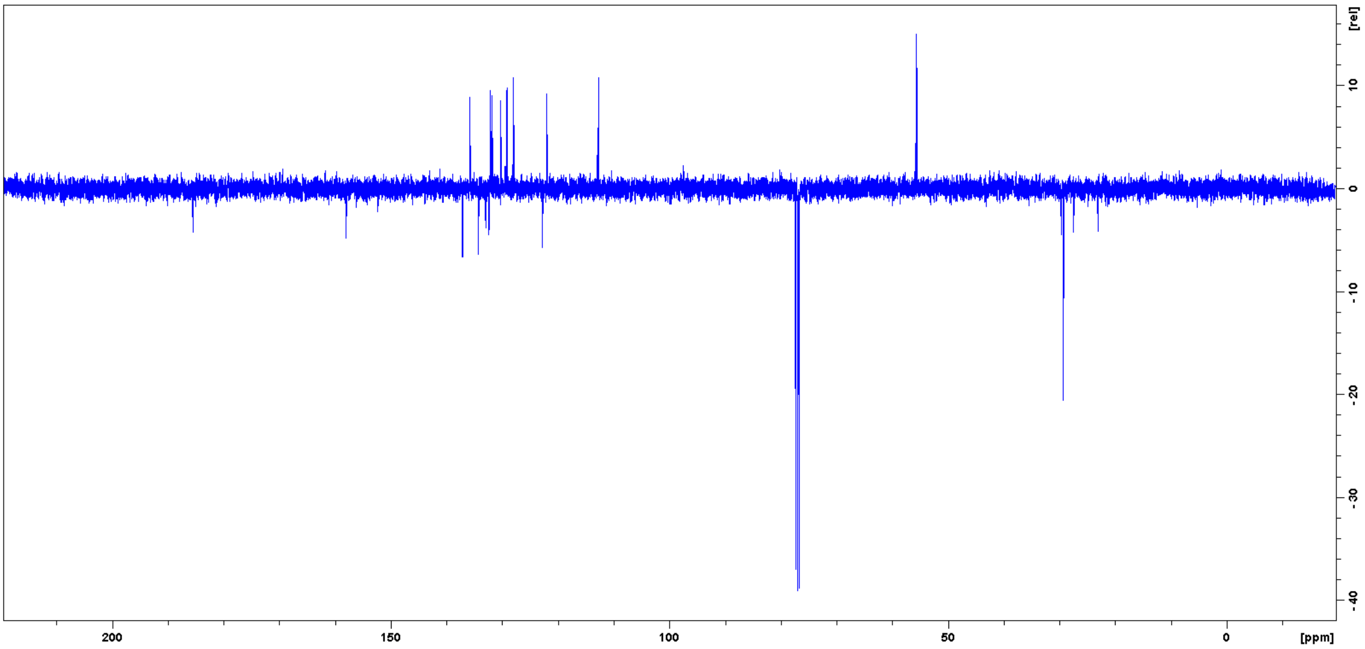

Figure S13. ^{1}H-NMR and ^{13}C-NMR spectra of compound **10**

(3*Z*)-6-bromo-3-[(4-fluorophenyl)methylidene]-2,3-dihydro-4H-1-benzothiopyran-4-one (11)

Light grey solid, 162 mg, 28.2 %, R_f = 0.80 (1:1 EtOAc:hexane), 136.8 - 137.4 °C, IR ν_{max} (cm^{-1}): 3055 (Ar-H), 1662 (C=O), 1598 (Aliphatic C=C), 1583 (Aromatic C=C), 904 (C-F), 784 (C-Br), 684 (C-S), ^{1}H-NMR (400 MHz, $CDCl_3$): δ = 8.32 (1H, d, J = 2.3 Hz, H-5), 7.77 (1H, brs w1/2 = , H-9), 7.43 (1H, dd, J = 2.3 Hz, 8.5 Hz, H-7), 7.41 (2H, dd, J =5.3 Hz, 9.0 Hz, H-2'), 7.22 (1H, d, 8.5 Hz, H-8), 7.14 (2H, t, J = 8.5 Hz, H-3'), 4.10 (2H, d, J = 1.1 Hz, H-2); ^{13}C-NMR (100 MHz, $CDCl_3$): δ = 184.6 (C-4), 164.2 (d, J = 244.9 Hz, C-4'), 119.6 (C-6), 137.4 (C-9), 135.8 (C-7), 131.8 (C-1'), 133.2 (C-5), 133.5 (C-4a), 131.4 (C-2'), 130.9 (C-3), 129.4 (C-8), 139.9(C-8a), 116.2 (d, J = 21.8 Hz, C-3'), 29.1 (C-2), HRESMS (ASAP) m/z 348.9695 $[M+H]^+$ (calcd $[C_{16}H_{11}OSBrF]$, 348.9698). Purity 93.1% (by NMR)

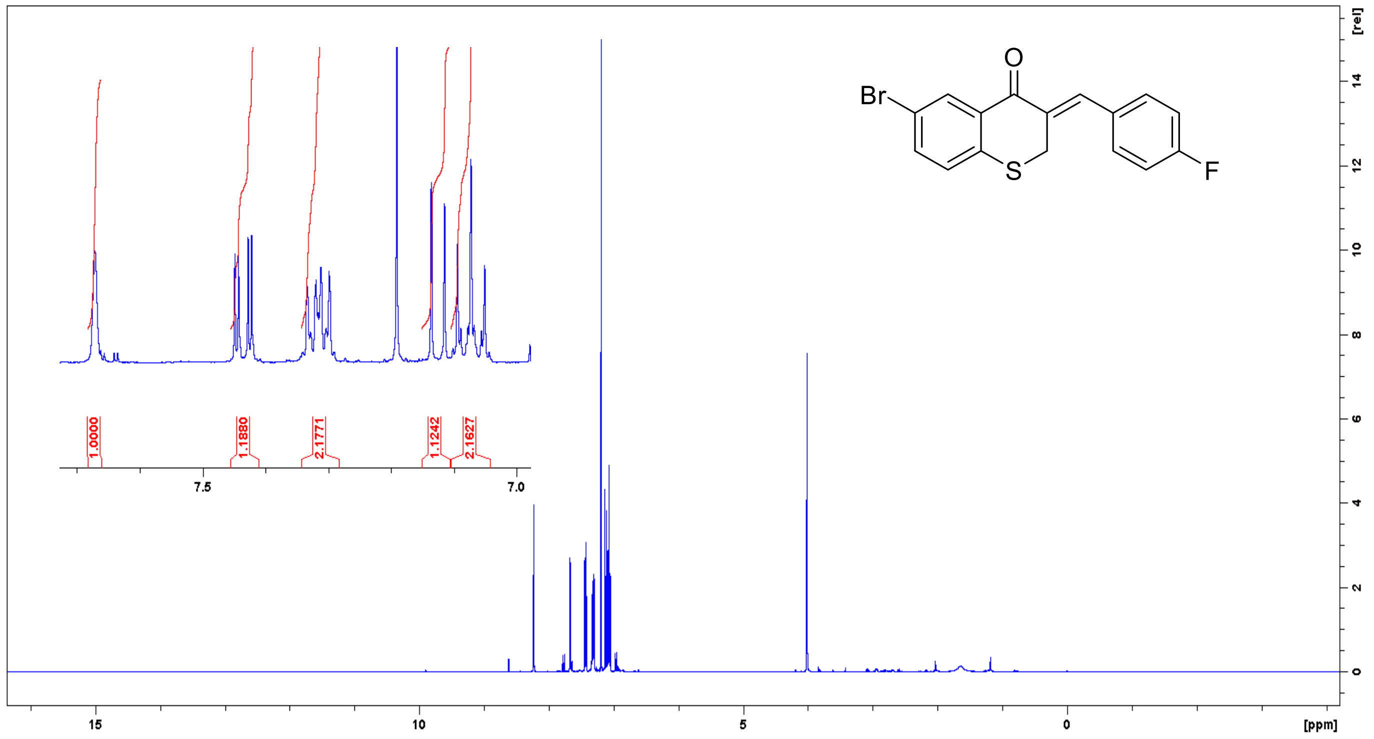

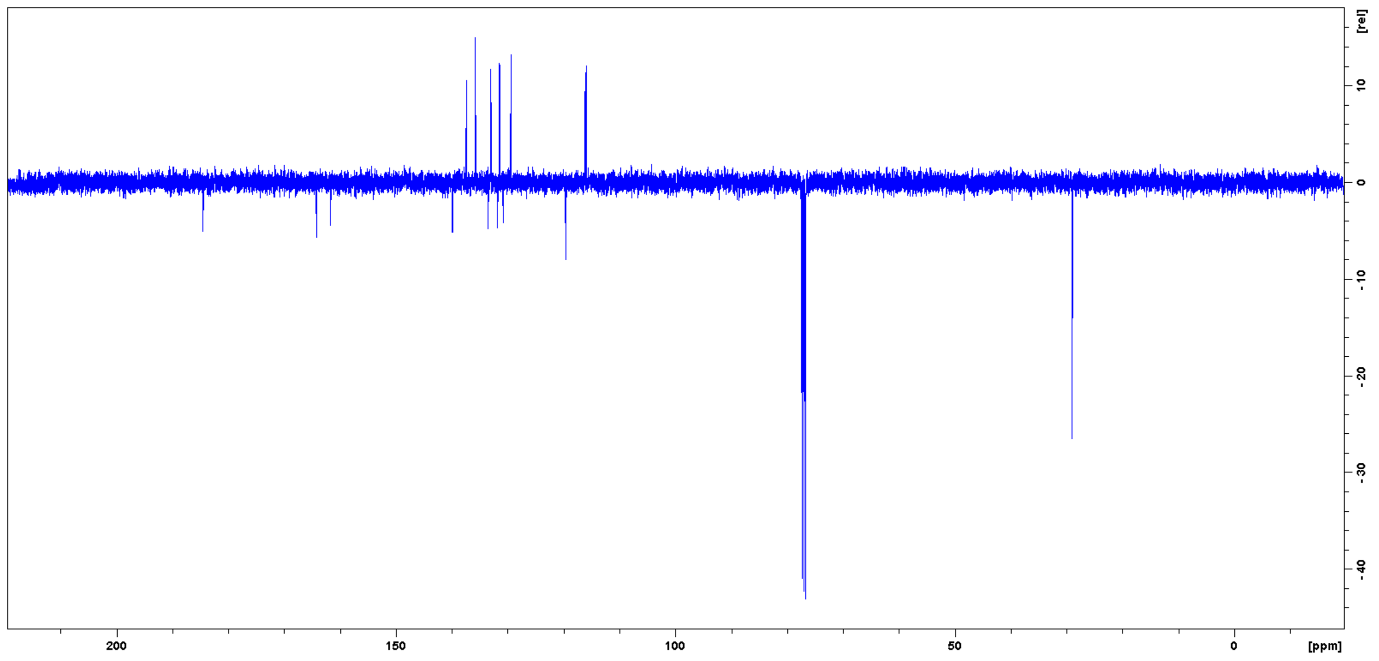

Figure S14. ^{1}H-NMR and ^{13}C-NMR spectra of compound **11**

(3*Z*)-6-Bromo-3-[(4-nitrophenyl)methylidene]-2,3-dihydro-4H-1-benzothiopyran-4-one (12)

Yellow solid, 221 mg, 35.5 %, R_f = 0.78 (1:1 EtOAc:hexane), 229.3 - 229.9 °C, IR ν_{max} (cm^{-1}): 3080 (Ar-H), 1663 (C=O), 1614 (Aliphatic C=C), 1594 (Aromatic C=C), 1511, 1340 (NO_2), 1289 (C-N), 777 (C-Br), 635 (C-S) ^{1}H-NMR (400 MHz, DMSO-d6): δ = 8.32 (2H, d, J = 8.7 Hz, H-2’), 8.13 (1H, d, J = 2.4 Hz, H-5), 7.81 (1H, d, J = 8.7 Hz, H-3’), 7.73 (1H, dd, J = 2.4, 8.4 Hz, H-7), 7.72 (1H, brs, $w_{1/2}$ = 1.8 Hz, H-9), 7.44 (1H, d, J = 8.4 Hz, H-8), 4.27 (2H, s, H-2), ^{13}C-NMR (100 MHz, DMSO-d6): δ = 184.5 (C-4), 147.7 (C-4’), 141.4 (C-3’), 140.8 (C-1’), 136.5 (C-7), 135.5 (C-8a), 135.3 (C-9), 133.5 (C-4a), 132.1 (C-5), 131.1 (C-3’), 130.5 (C-8) , 123.9 (C-2’), 118.7 (C-6), 28.5 (C-2) HRESMS (ASAP) *m/z* 375.9640 $[M+H]^+$ (calcd [$C_{16}H_{11}NO_3SBr$], 375.9643). Purity 70.0% (by NMR)

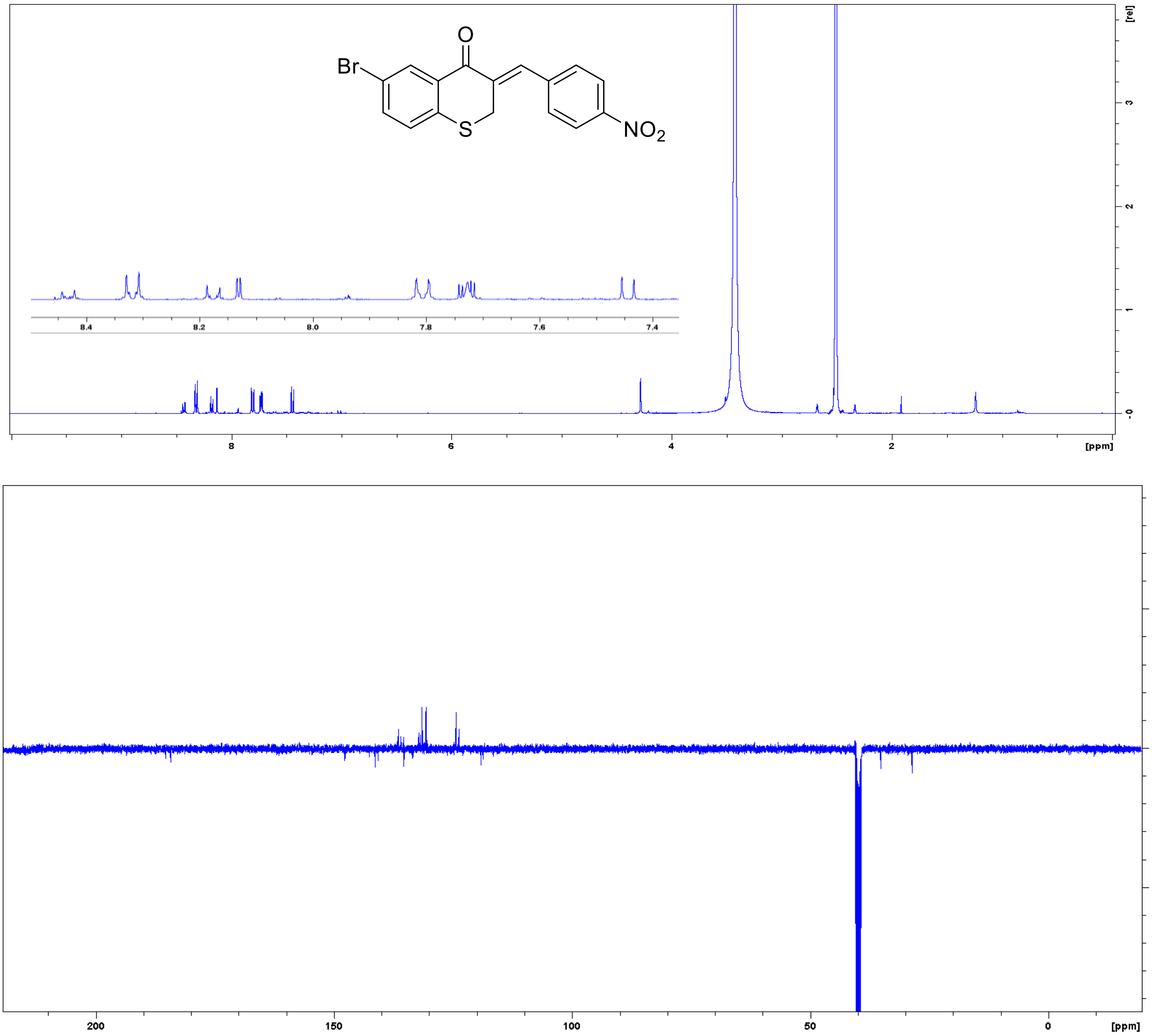

Figure S15. ^{1}H-NMR and ^{13}C-NMR spectra of compound **12**

(3*Z*)-6-Methoxy-3-[(4-nitrophenyl)methylidene]-2,3-dihydro-4H-1-benzothiopyran-4-one (13)

Yellow solid, 682 mg, 94.7 %, R_f = 0.73 (1:1 EtOAc:hexane), 176.3 - 176.7 °C, IR ν_{max} (cm^{-1}): 3012 (Ar-H), 1663 (C=O), 1589 (Aliphatic C=C), 1512 (Aromatic C=C), 1475, 1341 (NO_2), 1105 (C-O), 654 (C-S),^{1}H-NMR (400 MHz, $CDCl_3$): δ = 8.23 (2H, d, J = 8.8 Hz, H-2'), 7.67 (1H, brs, $w_{1/2}$ = 3.2 Hz, H-9), 7.62 (1H, d, J = 3.0 Hz, H-5), 7.48 (2H, s, J = 8.6 Hz, H-3'), 7.16 (1H, d, 8.8 Hz, H-8), 6.98 (1H, dd, J = 3.0 Hz, 8.8 Hz, H-7), 3.94 (2H, d, J = 1.1 Hz, H-2), 3.79 (3H, s, OCH_3); ^{13}C-NMR (100 MHz, $CDCl_3$): δ = 185.3 (C-4), 132.8 (C-4a), 147.7 (C-4'), 141.7 (C-1'), 136.3 (C-3), 134.6 (C-9), 136.1 (C-8a), 158.2 (C-6), 130.3 (C-3'), 129.4 (C-8), 124.0 (C-2'), 122.3 (C-7), 112.8 (C-5), 55.7 (OCH_3), 29.3 (C-2), Rf = 0.73 (1:1 EtOAc:hexane). HRESMS (ASAP) *m/z* 328.0644 $[M+H]^+$ (calcd [$C_{17}H_{14}NO_4S$], 328.0644). Purity 94.2% (by NMR)

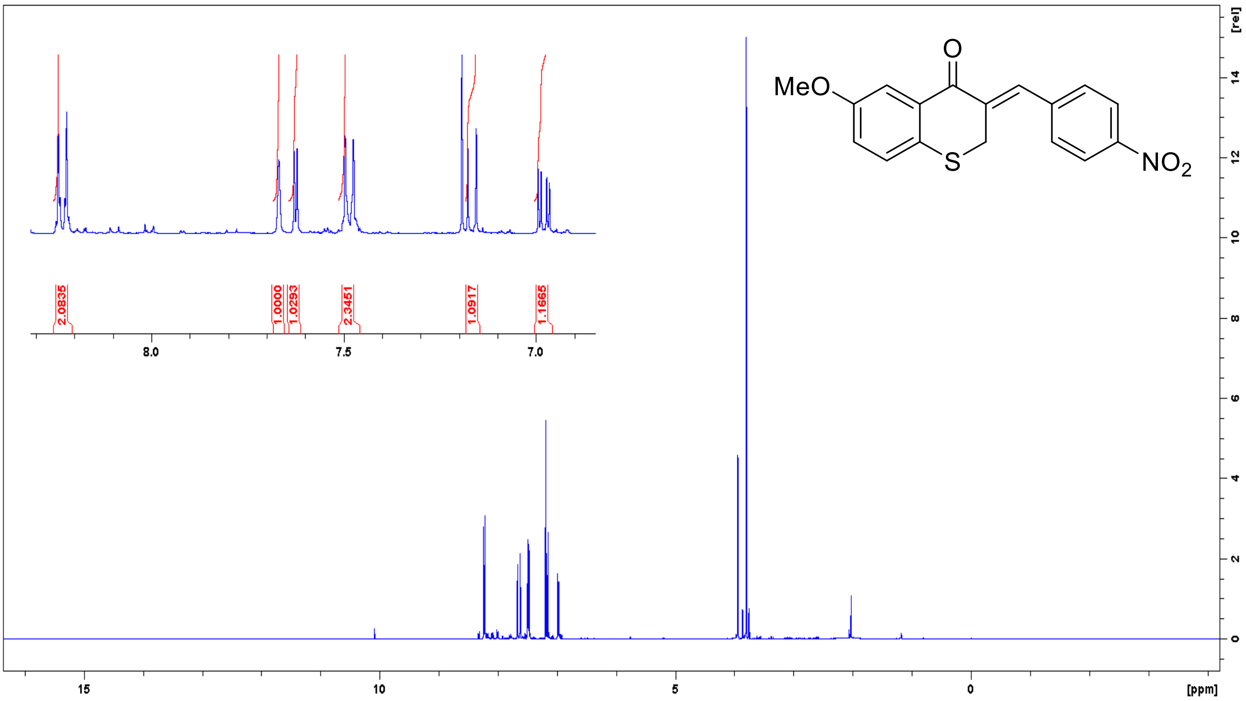

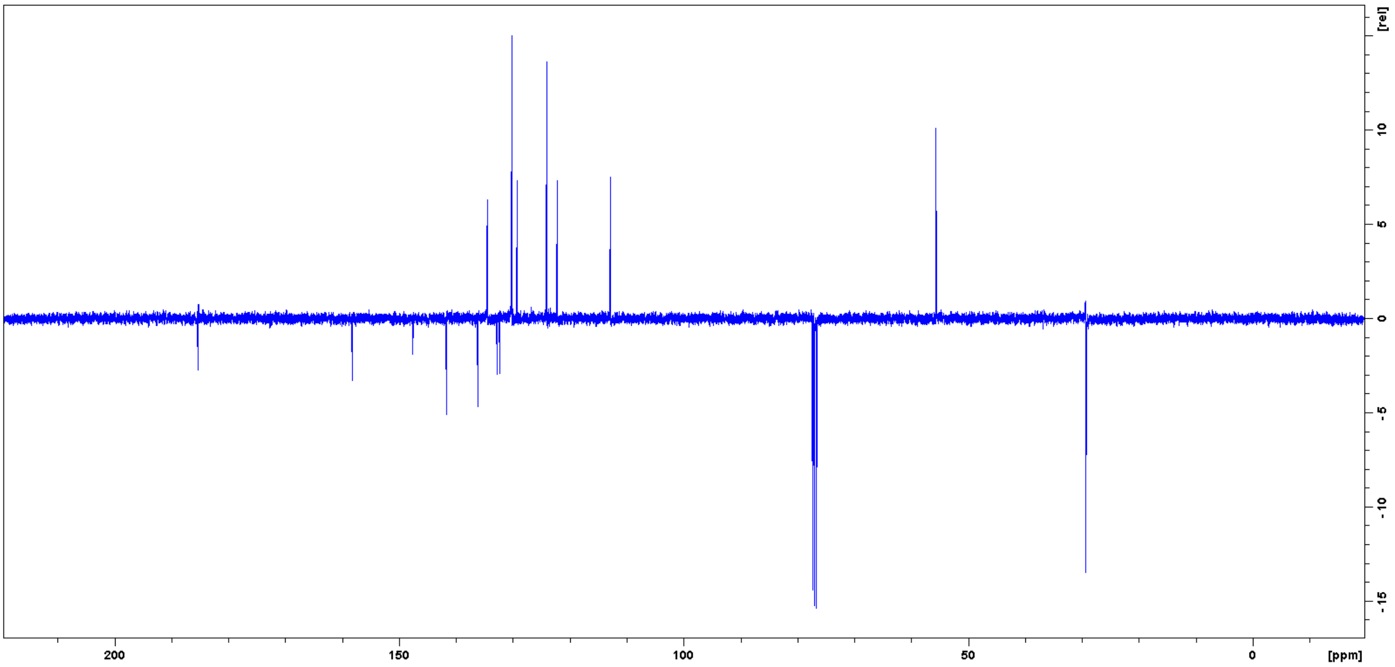

Figure S16. ^{1}H-NMR and ^{13}C-NMR spectra of compound **13**

Additional Experimental Details

SXD Measurements and Analysis

SXD measurements were made using an Agilent Oxford Diffraction SuperNova equipped with a microfocus Cu Kα X-ray source and an Atlas CCD detector. A single crystal was selected under a polarising microscope and mounted onto a nylon loop with the aid of Fomblin® oil before mounting on the diffractometer (Figure S18). Samples were cooled with a Cryojet5® developed by Oxford Instruments; the device used for these measurements is the original prototype and the Pt-resistance sensor is located in the copper-block heat exchanger and not in the nozzle of the instrument close to the sample. Thus the temperatures quoted in these SXD experiments should be treated as nominal (despite stability to much better than 0.1 °C). Total collection time was (typically 2 hours to 12 hours) for full spheres of data to a resolution of 0.84 Å. Data were collected using 1° scan frames in ω and reduced using the CrysAlis^{Pro} software package (version 1.171.39.46 from Rigaku Oxford Diffraction[1]). The structures were solved using intrinsic phasing by ShelXT[2] and refined by least-squares using ShelXL 2014[3] within the Olex2 program suite.[4] The position and anisotropic displacement factors were refined freely for the non-hydrogen atoms. Crystal structures are illustrated with the program Mercury from CCDC with thermal ellipsoids (including H atoms) shown at 50% probability.[5]

[1] https://www.rigaku.com/en/products/smc/crysalis
[2] G. M. Sheldrick, *Acta Crystallogr., Sect. A: Found. Adv.*, 2015, **71**, 3–8.
[3] G. M. Sheldrick, *Acta Crystallogr., Sect. C: Struct. Chem.*, 2015, **71**, 3–8.
[4] O. V. Dolomanov, L. J. Bourhis, R. J. Gildea, J. A. K. Howard and H. Puschmann, *J. Appl. Crystallogr.*, 2009, **42**, 339-341.

Table S1a. Crystal data and structure refinement for compound **5** ($C_{16}H_{10}BrClOS$) at 150 K.

Identification code	exp_1634
Empirical formula	$C_{16}H_{10}BrClOS$
Formula weight	365.66
Temperature / K	150
Crystal system	monoclinic
Space group	$P2_1/n$
a / Å	3.98329(13)
b / Å	28.4441(6)
c / Å	12.1203(2)
α / °	90
β / °	90.338(2)
γ / °	90
Volume / Å^3	1373.22(6)
Z	4
ρ_{calc} / g cm^{-3}	1.769
μ / mm^{-1}	7.212
F(000)	728.0
Crystal size / mm^3	0.402 × 0.089 × 0.039
Radiation	Cu Kα (λ = 1.54184 Å)
2θ range for data collection / °	7.93 to 146.438
Index ranges	$-4 \le h \le 4$, $-34 \le k \le 35$, $-14 \le l \le 14$
Reflections collected	21083
Independent reflections	2681 [R_{int} = 0.0371, R_{sigma} = 0.0180]
Data/restraints/parameters	2681/0/221
Goodness-of-fit on F^2	1.070
Final R indexes [$I \ge 2\sigma(I)$]	R_1 = 0.0265, wR_2 = 0.0584
Final R indexes [all data]	R_1 = 0.0282, wR_2 = 0.0592
Largest diff. peak/hole / e Å^{-3}	0.40/−0.28

Table S1b. Fractional atomic coordinates and equivalent isotropic displacement parameters for compound **5** ($C_{16}H_{10}BrClOS$) at 150 K. U_{eq} is defined as ⅓ of the trace of the orthogonalised U_{ij} tensor.

**Atom**	***x***	***y***	***z***	***U*(eq) / Å²**
Br(1)	0.89298(6)	0.49214(2)	0.67600(2)	0.02693(8)
Cl(1)	−0.22653(15)	0.04946(2)	0.43971(4)	0.02671(13)
S(1)	0.45168(14)	0.19598(2)	0.75727(4)	0.01937(12)
O(1)	0.2530(5)	0.21899(5)	0.40490(12)	0.0310(4)
C(1)	0.2722(5)	0.15596(7)	0.66388(16)	0.0171(4)
C(2)	0.2268(6)	0.10928(7)	0.69814(17)	0.0207(4)
C(3)	0.0785(6)	0.07643(8)	0.62993(18)	0.0219(5)
C(4)	−0.0296(6)	0.09018(7)	0.52485(17)	0.0201(4)
C(5)	0.0201(6)	0.13534(7)	0.48825(17)	0.0196(4)
C(6)	0.1747(5)	0.16864(7)	0.55596(16)	0.0173(4)
C(7)	0.2582(6)	0.21509(7)	0.50575(16)	0.0193(4)
C(8)	0.3514(5)	0.25520(7)	0.57903(16)	0.0177(4)
C(9)	0.2718(6)	0.24929(7)	0.69913(16)	0.0177(4)
C(10)	0.4676(6)	0.29399(7)	0.52882(16)	0.0189(4)
C(11)	0.5674(6)	0.34018(7)	0.57132(16)	0.0185(4)
C(12)	0.5198(6)	0.37861(8)	0.50093(18)	0.0228(5)
C(13)	0.6088(6)	0.42394(8)	0.53126(18)	0.0237(5)
C(14)	0.7550(5)	0.43057(7)	0.63392(17)	0.0184(4)
C(15)	0.8110(6)	0.39369(8)	0.70564(18)	0.0209(4)
C(16)	0.7156(6)	0.34854(7)	0.67438(17)	0.0191(4)
H(2)	0.299(6)	0.1001(9)	0.772(2)	0.026(7)
H(3)	0.056(6)	0.0463(9)	0.650(2)	0.025(6)
H(5)	−0.052(7)	0.1448(9)	0.418(2)	0.030(7)
H(9A)	0.350(6)	0.2743(9)	0.742(2)	0.020(6)
H(9B)	0.044(7)	0.2464(8)	0.7066(19)	0.019(6)
H(10)	0.480(6)	0.2916(9)	0.450(2)	0.024(6)
H(12)	0.417(7)	0.3756(9)	0.435(2)	0.026(7)
H(13)	0.571(7)	0.4498(9)	0.484(2)	0.027(7)
H(15)	0.906(6)	0.3989(8)	0.772(2)	0.018(6)
H(16)	0.759(6)	0.3244(9)	0.720(2)	0.024(6)

Table S1c. Anisotropic displacement parameters for compound **5** ($C_{16}H_{10}BrClOS$) at 150 K. The anisotropic displacement factor exponent has the form: $-2\pi^2[h^2a^{*2}U_{11}+2hka^*b^*U_{12}+\ldots]$.

Atom	U_{11} / Å^2	U_{22} / Å^2	U_{33} / Å^2	U_{23} / Å^2	U_{13} / Å^2	U_{12} / Å^2
Br(1)	0.03119(15)	0.01716(12)	0.03241(14)	−0.00185(8)	−0.00307(10)	−0.00349(9)
Cl(1)	0.0320(3)	0.0176(2)	0.0305(3)	−0.00374(19)	−0.0073(2)	−0.0019(2)
S(1)	0.0230(3)	0.0222(2)	0.0129(2)	0.00041(18)	−0.00212(19)	−0.0014(2)
O(1)	0.0548(12)	0.0247(8)	0.0134(7)	0.0009(6)	−0.0037(7)	−0.0114(8)
C(1)	0.0152(11)	0.0216(10)	0.0147(9)	−0.0016(7)	0.0011(8)	0.0022(8)
C(2)	0.0225(12)	0.0234(11)	0.0163(10)	0.0034(8)	0.0010(8)	0.0045(9)
C(3)	0.0256(13)	0.0169(10)	0.0233(11)	0.0019(8)	0.0039(9)	0.0018(8)
C(4)	0.0206(12)	0.0180(10)	0.0218(10)	−0.0038(8)	0.0021(8)	0.0000(8)
C(5)	0.0211(12)	0.0221(10)	0.0155(10)	−0.0003(8)	0.0004(8)	0.0019(8)
C(6)	0.0168(11)	0.0189(10)	0.0162(9)	−0.0005(7)	0.0033(8)	0.0009(8)
C(7)	0.0231(12)	0.0196(10)	0.0151(10)	0.0000(8)	−0.0010(8)	−0.0001(8)
C(8)	0.0189(11)	0.0189(10)	0.0153(9)	−0.0018(7)	0.0003(8)	−0.0001(8)
C(9)	0.0204(12)	0.0187(10)	0.0138(9)	−0.0012(8)	0.0018(8)	−0.0018(8)
C(10)	0.0214(12)	0.0222(10)	0.0131(9)	0.0002(8)	0.0010(8)	0.0006(8)
C(11)	0.0201(12)	0.0194(10)	0.0159(9)	0.0000(8)	0.0035(8)	−0.0013(8)
C(12)	0.0267(13)	0.0257(11)	0.0159(10)	0.0012(8)	−0.0029(9)	−0.0042(9)
C(13)	0.0294(13)	0.0200(10)	0.0217(10)	0.0053(8)	−0.0013(9)	−0.0014(9)
C(14)	0.0166(11)	0.0146(9)	0.0239(10)	−0.0032(8)	0.0025(8)	−0.0034(8)
C(15)	0.0214(12)	0.0234(11)	0.0179(10)	−0.0013(8)	−0.0026(9)	−0.0002(9)
C(16)	0.0220(12)	0.0178(10)	0.0175(10)	0.0020(8)	−0.0015(8)	0.0012(8)

Table S1d. Selected bond lengths for compound **5** ($C_{16}H_{10}BrClOS$) at 150 K.

Atom — Atom	Length / Å	Atom — Atom	Length / Å
Br(1) — C(14)	1.904(2)	C(6) — C(7)	1.493(3)
Cl(1) — C(4)	1.735(2)	C(7) — C(8)	1.491(3)
S(1) — C(1)	1.755(2)	C(8) — C(9)	1.501(3)
S(1) — C(9)	1.818(2)	C(8) — C(10)	1.343(3)
O(1) — C(7)	1.227(3)	C(10) — C(11)	1.465(3)
C(1) — C(2)	1.403(3)	C(11) — C(12)	1.399(3)
C(1) — C(6)	1.409(3)	C(11) — C(16)	1.399(3)
C(2) — C(3)	1.378(3)	C(12) — C(13)	1.386(3)
C(3) — C(4)	1.398(3)	C(13) — C(14)	1.384(3)
C(4) — C(5)	1.374(3)	C(14) — C(15)	1.380(3)
C(5) — C(6)	1.394(3)	C(15) — C(16)	1.391(3)

Table S1e. Selected bond angles for compound **5** ($C_{16}H_{10}BrClOS$) at 150 K.

Atom — Atom — Atom	Angle / °	Atom — Atom — Atom	Angle / °
C(9) — S(1) — C(1)	97.64(10)	C(7) — C(8) — C(9)	116.01(17)
S(1) — C(1) — C(6)	122.79(16)	C(8) — C(9) — S(1)	112.60(15)
C(1) — C(6) — C(7)	122.97(19)	C(8) — C(10) — C(11)	132.11(19)
C(6) — C(7) — C(8)	119.31(17)		

Torsion angles of interest: C(7) — C(8) — C(9) — S(1) = 54.7(2)°
S(1) — C(9) — C(8) — C(10) = 132.0(2)°

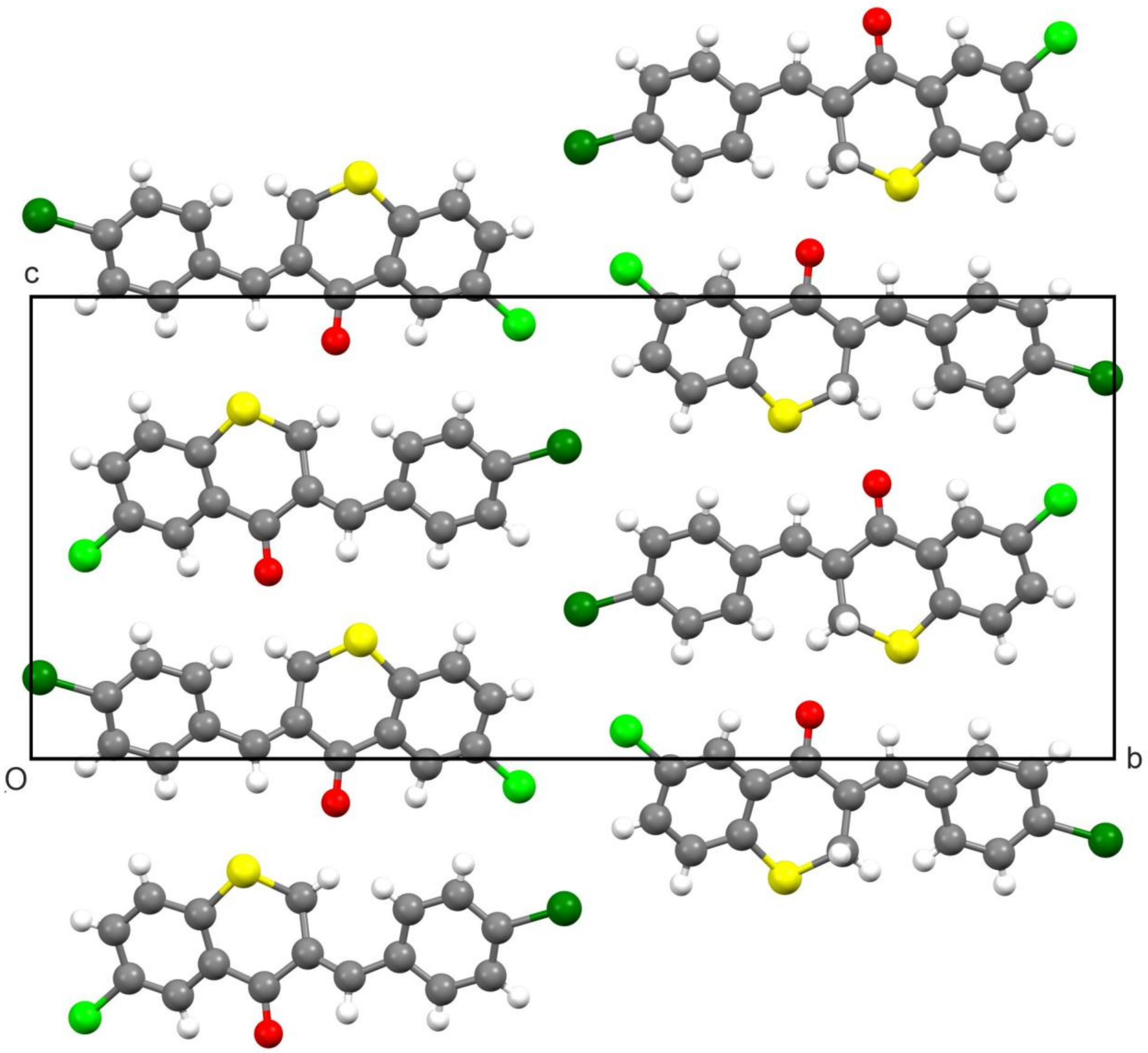

Figure S17. Unit cell showing the structure of compound **5** ($C_{16}H_{10}BrClOS$) (C = grey, Cl = green, H = white, S = yellow and Br = dark green) at 150 K, as viewed down **a**.

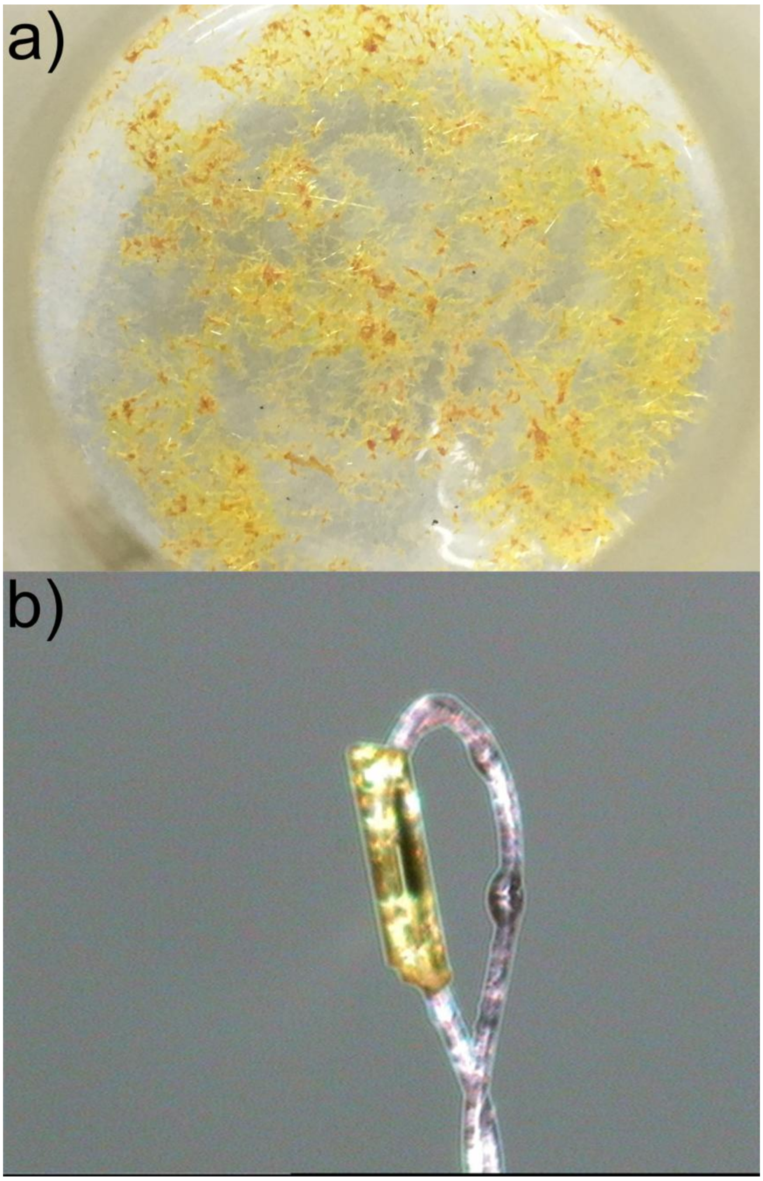

Figure S18. Photographs of crystals of compound **5** ($C_{16}H_{10}BrClOS$). a) is a photograph of the as-synthesised crystals and b) a single crystal mounted on the nylon loop *in situ* on the single crystal diffractometer.

Biological Data

Compound **4**

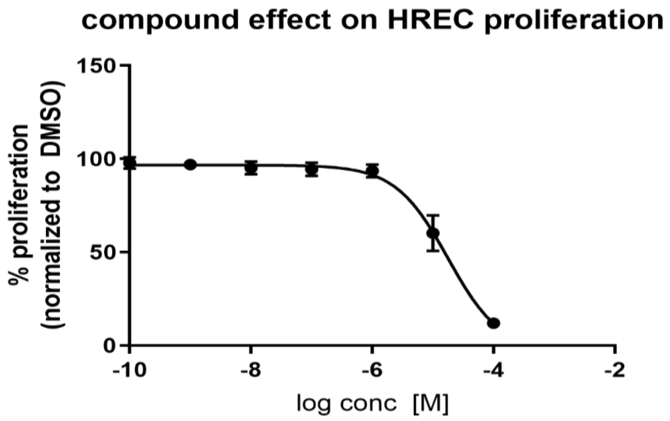

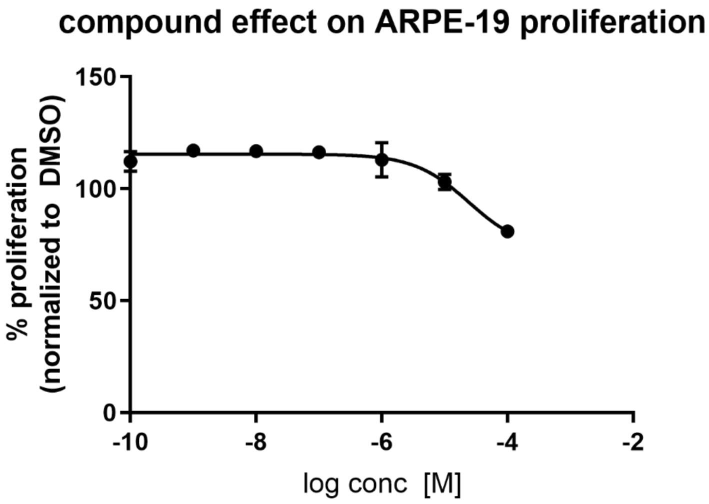

Figure S19. Biological Data for Compound **4** (a) Cytotoxicity against HeLa cells, (b) anti-proliferation against HREC, (c) anti-proliferation against ARPE-19.

Compound 5

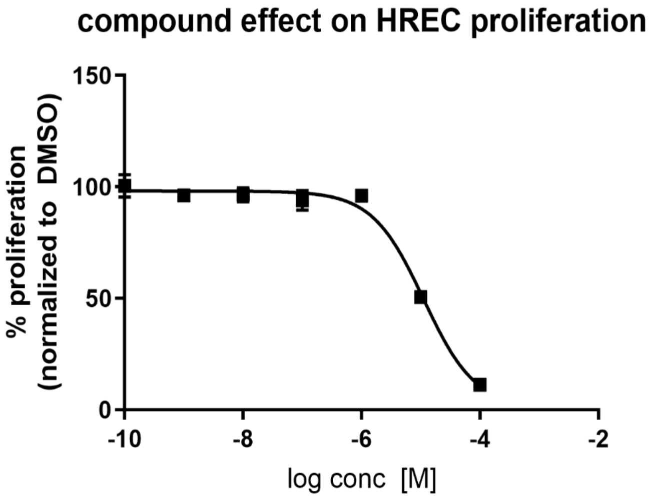

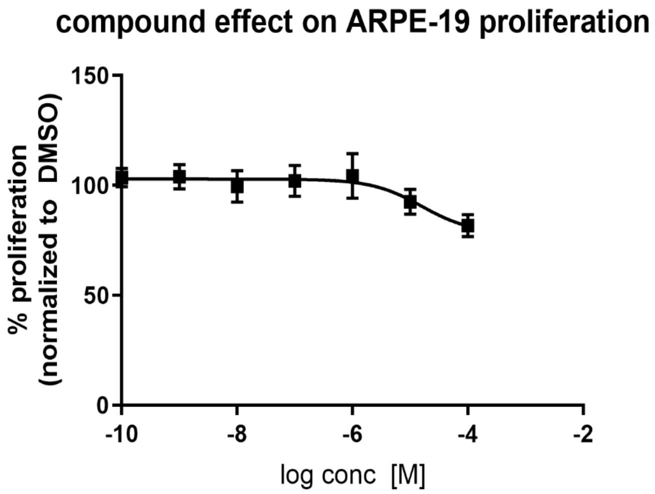

Figure S20. Biological Data for Compound **5** (a) Cytotoxicity against HeLa cells, (b) anti-proliferation against HREC, (c) anti-proliferation against ARPE-19.

Compound 6

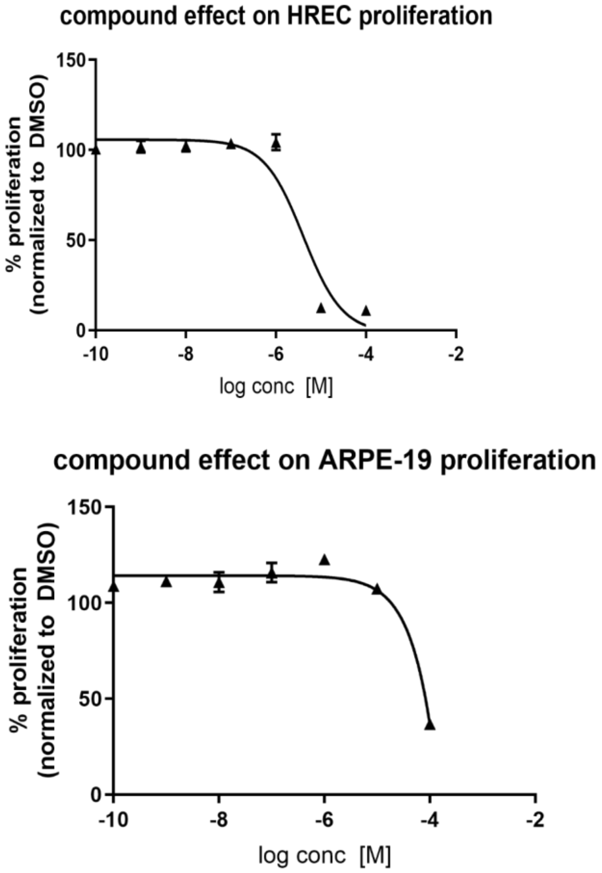

Figure S21. Biological Data for Compound **6** (a) Cytotoxicity against HeLa cells, (b) anti-proliferation against HREC, (c) anti-proliferation against ARPE-19.

Compound 7

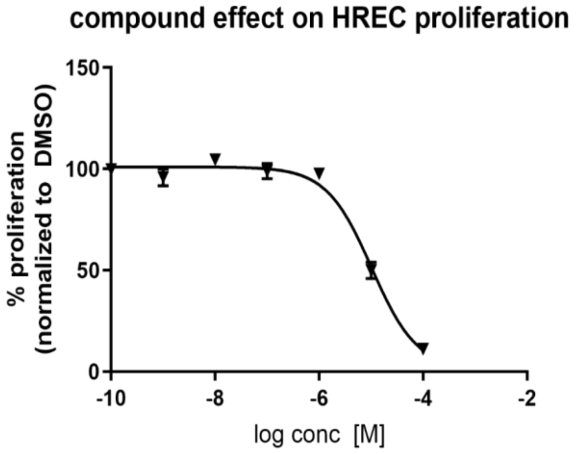

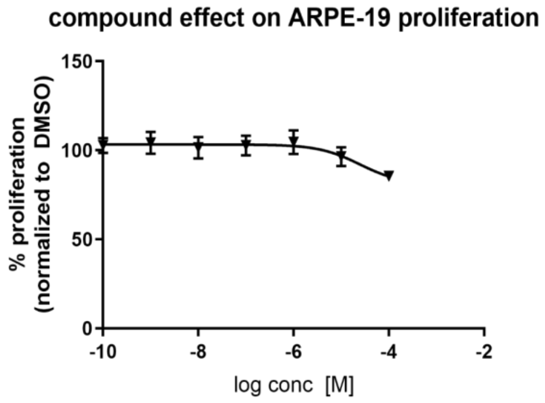

Figure S22. Biological Data for Compound **7** (a) Cytotoxicity against HeLa cells, (b) anti-proliferation against HREC, (c) anti-proliferation against ARPE-19.

Compound 8

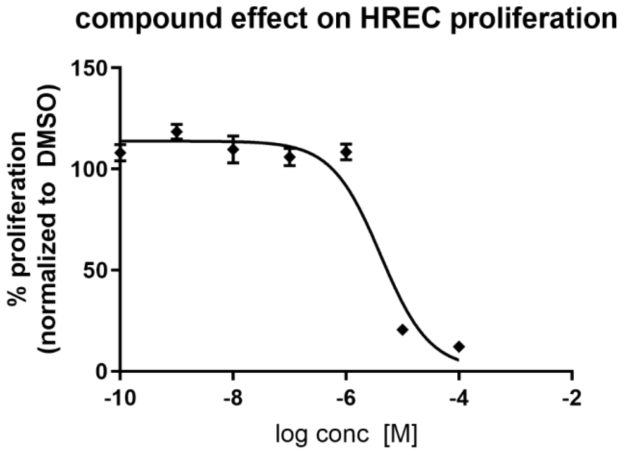

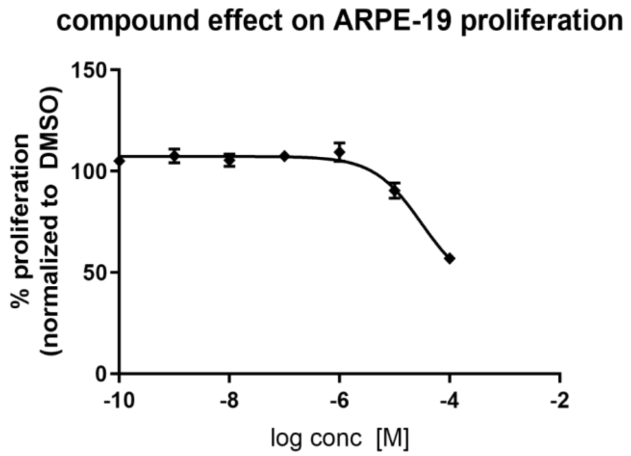

Figure S23. Biological Data for Compound **8** (a) Cytotoxicity against HeLa cells, (b) anti-proliferation against HREC, (c) anti-proliferation against ARPE-19.

Compound 9

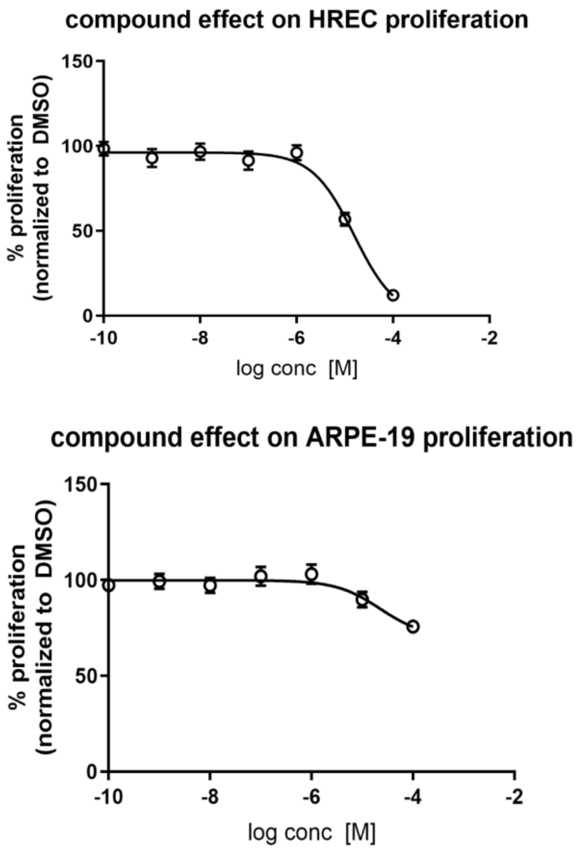

Figure S24. Biological Data for Compound **9** (a) Cytotoxicity against HeLa cells, (b) anti-proliferation against HREC, (c) anti-proliferation against ARPE-19

Compound **10**

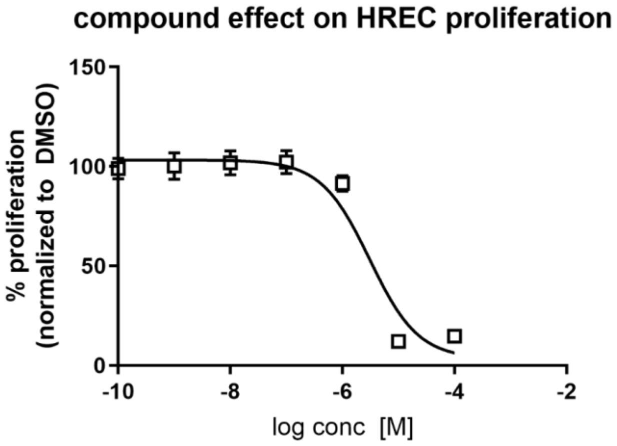

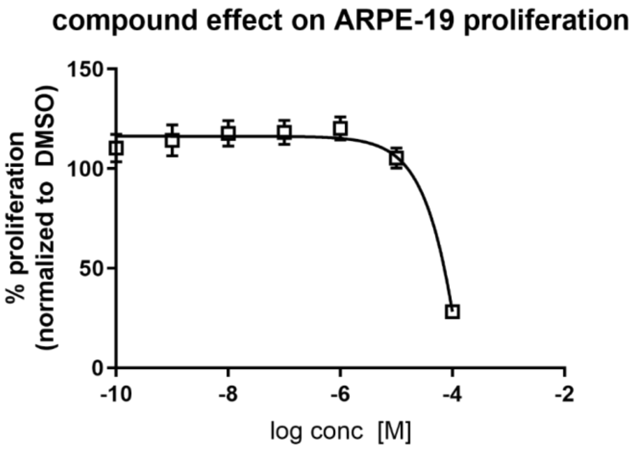

Figure S25. Biological Data for Compound **10** (a) Cytotoxicity against HeLa cells, (b) anti-proliferation against HREC, (c) anti-proliferation against ARPE-19.

Compound **11**

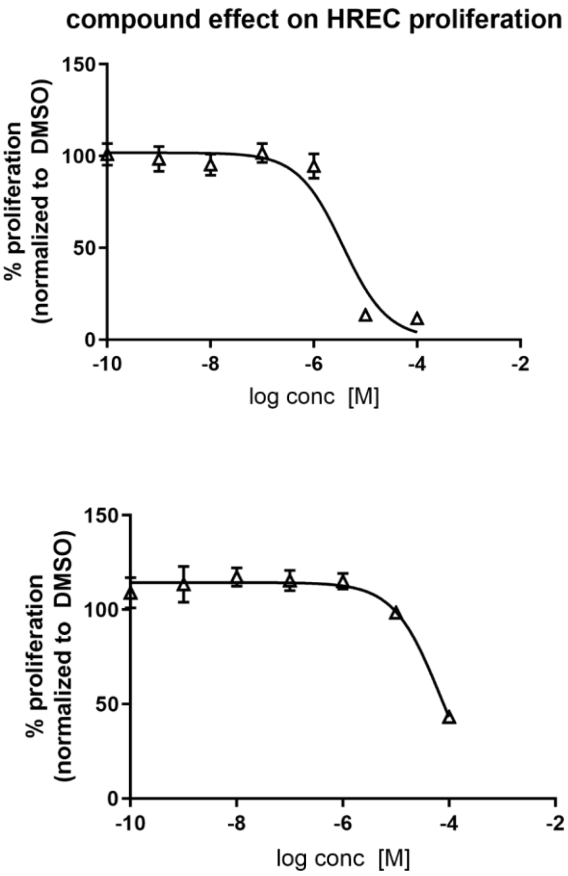

Figure S26. Biological Data for Compound **11** (a) Cytotoxicity against HeLa cells, (b) anti-proliferation against HREC, (c) anti-proliferation against ARPE-19.

Compound 12

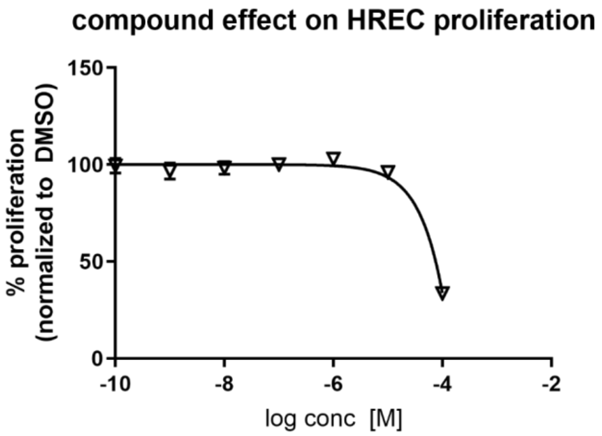

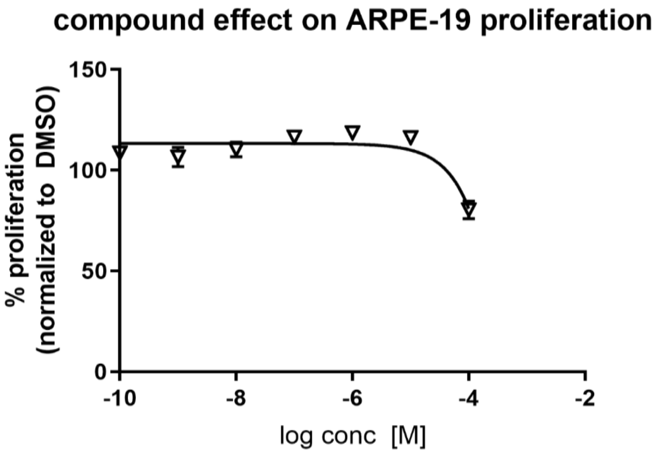

Figure S27. Biological Data for Compound **12** (a) Cytotoxicity against HeLa cells, (b) anti-proliferation against HREC, (c) anti-proliferation against ARPE-19.

Compound 13

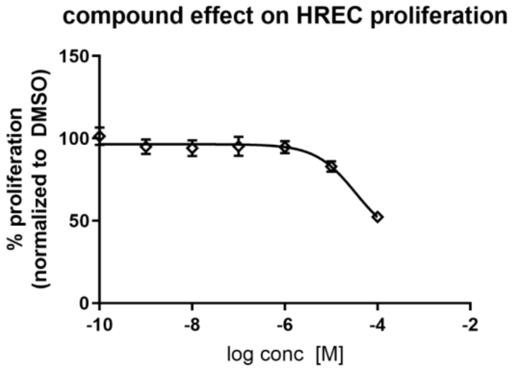

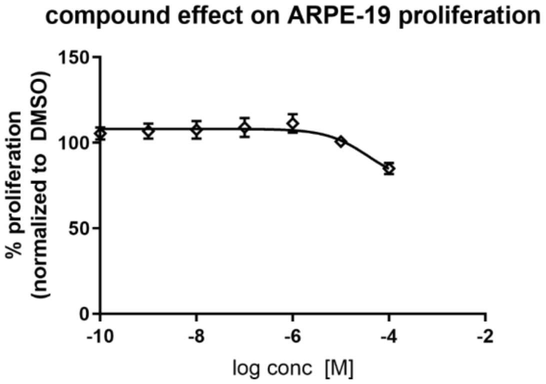

Figure S28. Biological Data for Compound **13** (a) Cytotoxicity against HeLa cells, (b) anti-proliferation against HREC, (c) anti-proliferation against ARPE-19.

Figure S29. Images of Matrigel experiments for compounds 4, 5, 6, 10, 11 and 13

Compound 4

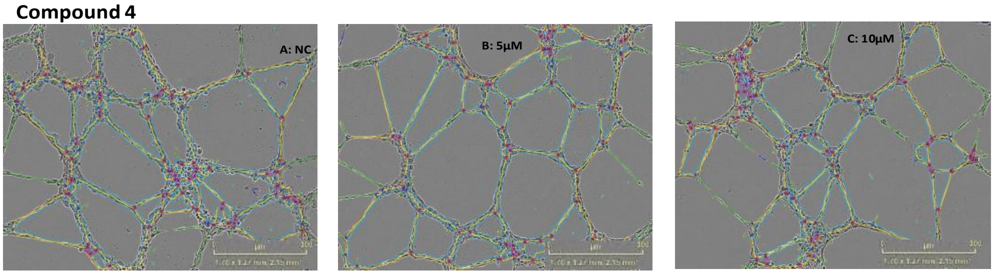

*Comparative network development in tube formation assay where; A: represents the negative control, B: compound 5 at 5 µM, C: compound 5 at 10 µM. Images were analysed using Incucyte software, data was statistically analysed with Graph Pad Prism. Results presented as mean ± SD of three independent experiments (one-way ANOVA against negative control). * P ≤ 0.05.*

Compound 5

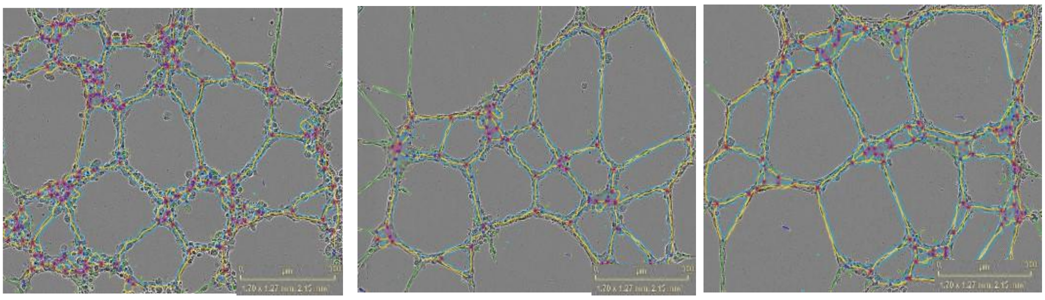

A: NC B: 5µM C: 10µM

- *Comparative network development in tube formation assay where; A: represents the negative control, B: compound 5 at 5 µM, C: compound 5 at 10 µM. Images were analysed using Incucyte software, data was statistically analysed with Graph Pad Prism. Results presented as mean ± SD of three independent experiments (one-way ANOVA against negative control). * P ≤ 0.05.*

Compound 6

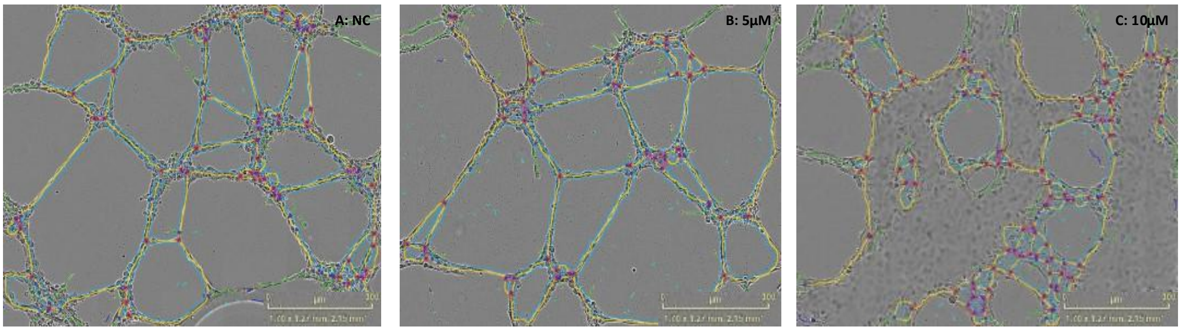

- *Comparative network development in tube formation assay where; A: represents the negative control, B: compound 6 at 5 µM, C: compound 6 at 10 µM. Images were analysed using Incucyte software, data was statistically analysed with Graph Pad Prism. Results presented as mean ± SD of three independent experiments (one-way ANOVA against negative control). * P ≤ 0.05.*

Compound 10

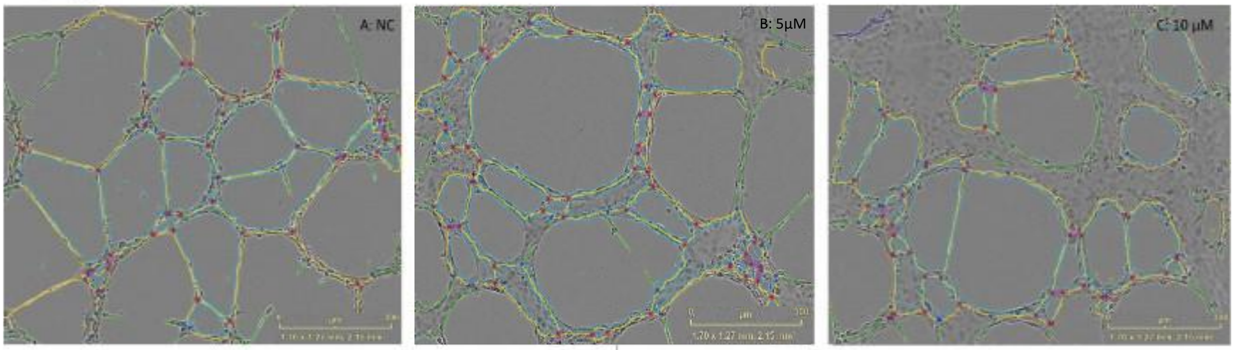

- *Comparative network development in tube formation assay where; A: represents the negative control, B: compound 10 at 5 µM, C: compound 10 at 10 µM. Images were analysed using Incucyte software, data was statistically analysed with Graph Pad Prism. Results presented as mean ± SD of three independent experiments (one-way ANOVA against negative control). * P ≤ 0.05.*

Compound 11

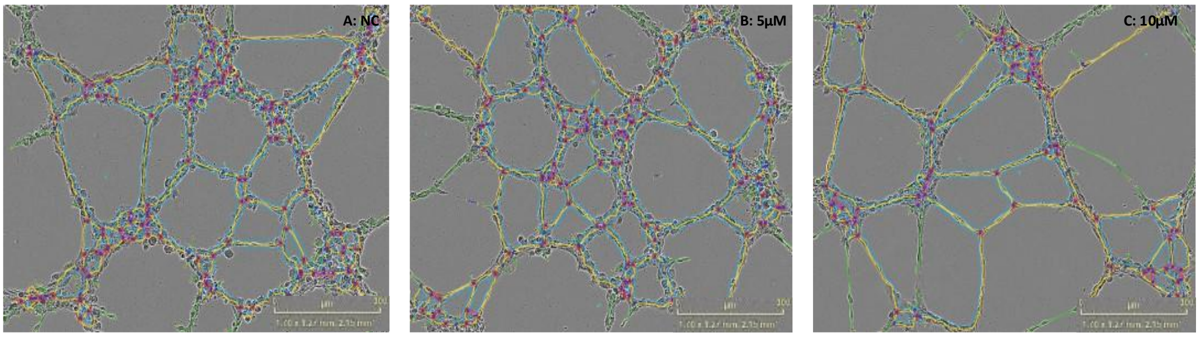

- *Comparative network development in tube formation assay where; A: represents the negative control, B: compound 11 at 5 µM, C: compound 11 at 10 µM. Images were analysed using Incucyte software, data was statistically analysed with Graph Pad Prism. Results presented as mean ± SD of three independent experiments (one-way ANOVA against negative control). * P ≤ 0.05.*

Compound 13

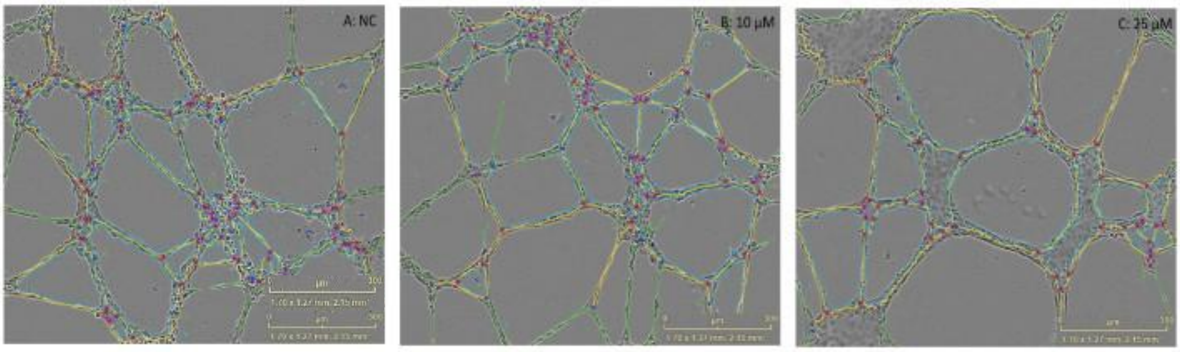

- *Comparative network development in tube formation assay where; A: represents the negative control, B: compound 13 at 10 µM, C: compound 13 at 25 µM. Images were analysed using Incucyte software, data was statistically analysed with Graph Pad Prism. Results presented as mean ± SD of three independent experiments (one-way ANOVA against negative control). * P ≤ 0.05.*

Figure S30. HRMS data

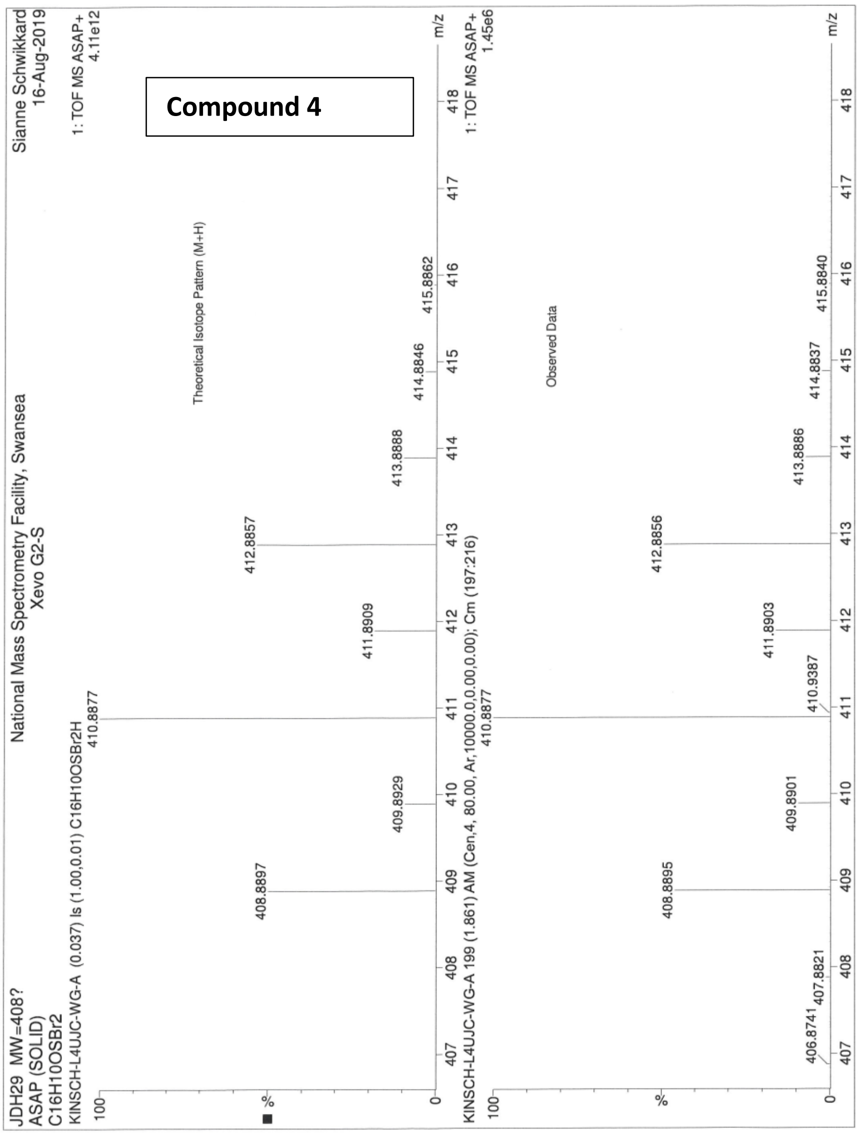

Compound 5

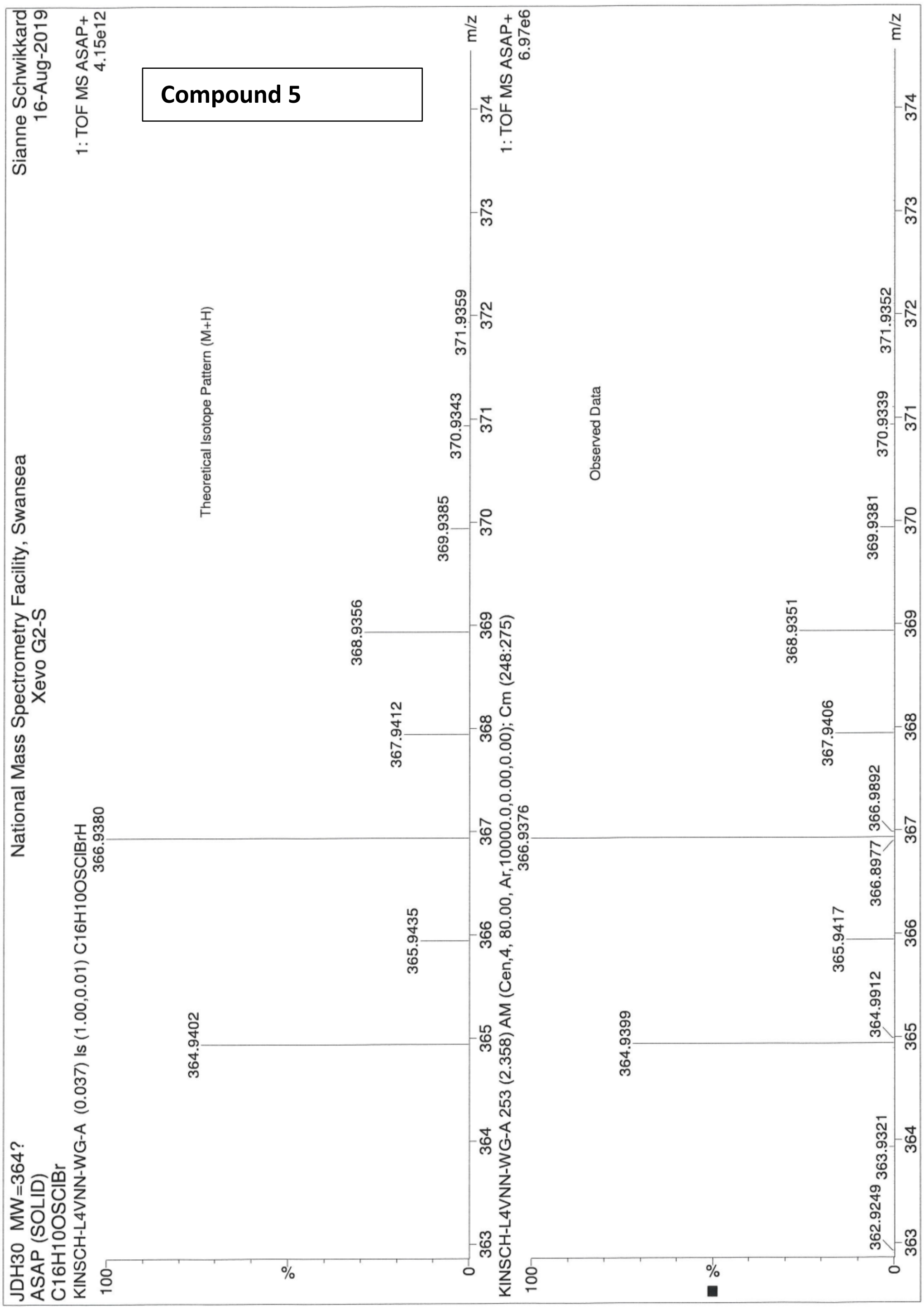

JDH30 MW=364?
ASAP (SOLID)
C16H10OSClBr
National Mass Spectrometry Facility, Swansea
Xevo G2-S
Sianne Schwikkard
16-Aug-2019
KINSCH-L4VNN-WG-A (0.037) Is (1.00,0.01) C16H10OSClBrH
1: TOF MS ASAP+
4.15e12
Theoretical Isotope Pattern (M+H)
364.9402
365.9435
366.9380
367.9412
368.9356
369.9385
370.9343
371.9359
KINSCH-L4VNN-WG-A 253 (2.358) AM (Cen,4, 80.00, Ar,10000.0,0.00,0.00); Cm (248:275)
1: TOF MS ASAP+
6.97e6
Observed Data
362.9249
363.9321
364.9399
364.9912
365.9417
366.8977
366.9376
366.9892
367.9406
368.9351
369.9381
370.9339
371.9352
%
m/z

Compound 6

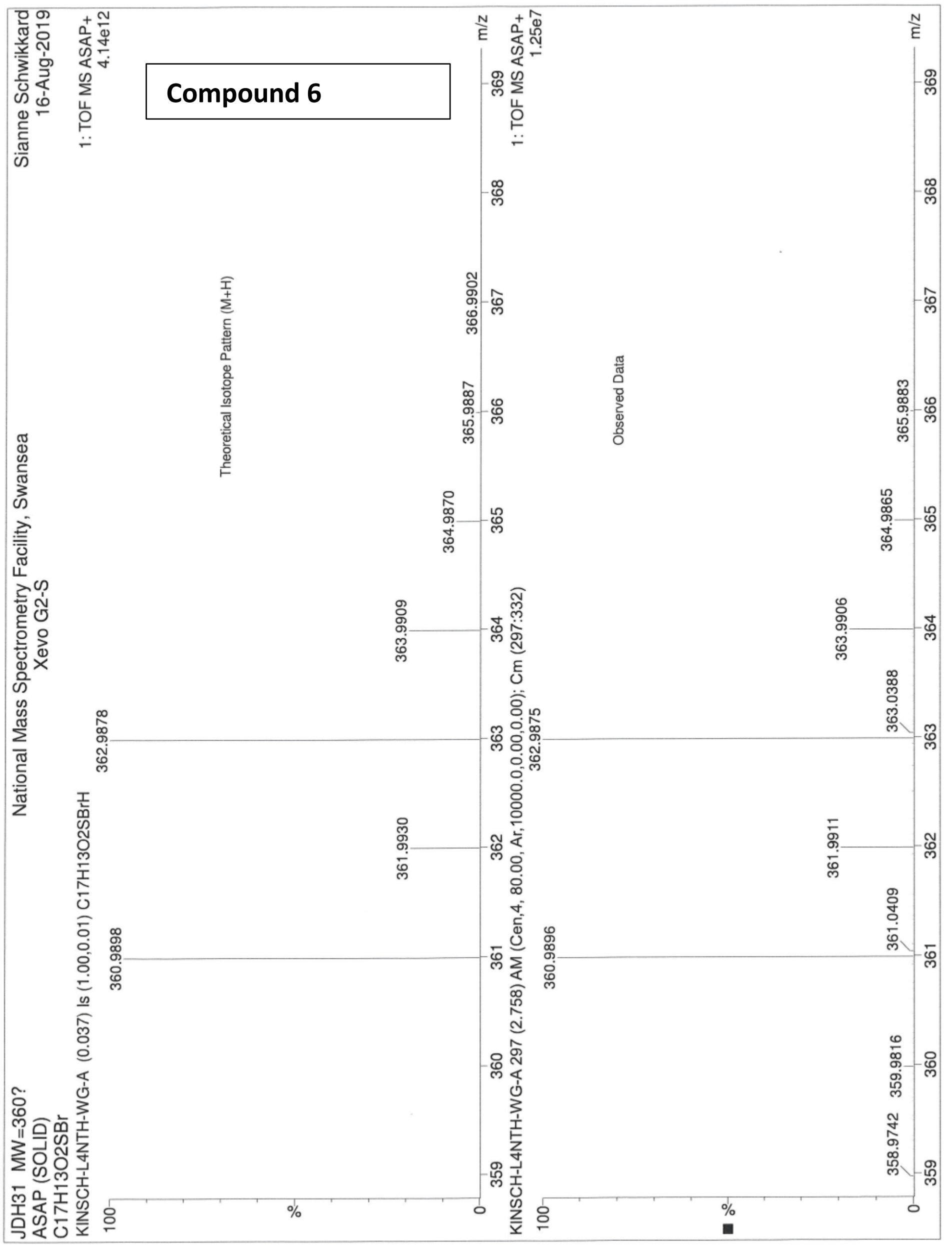
JDH31 MW=360?
ASAP (SOLID)
C17H13O2SBr
National Mass Spectrometry Facility, Swansea
Xevo G2-S
Sianne Schwikkard
16-Aug-2019
KINSCH-L4NTH-WG-A (0.037) Is (1.00,0.01) C17H13O2SBrH
1: TOF MS ASAP+
4.14e12
Theoretical Isotope Pattern (M+H)
360.9898
361.9930
362.9878
363.9909
364.9870
365.9887
366.9902
KINSCH-L4NTH-WG-A 297 (2.758) AM (Cen,4, 80.00, Ar,10000.0,0.00,0.00); Cm (297:332)
1: TOF MS ASAP+
1.25e7
Observed Data
358.9742
359.9816
360.9896
361.0409
361.9911
362.9875
363.0388
363.9906
364.9865
365.9883
%
m/z

Compound 7

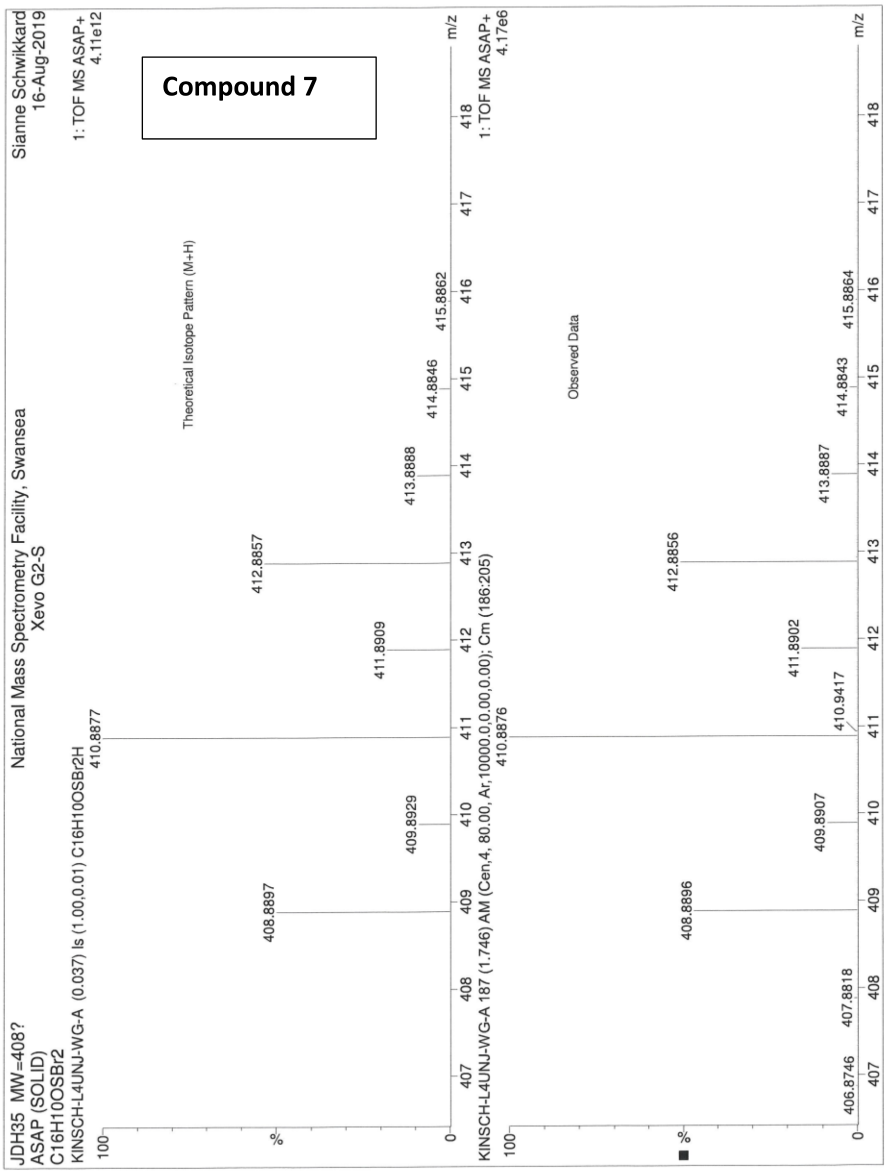
JDH35 MW=408?
ASAP (SOLID)
C16H10OSBr2
KINSCH-L4UNJ-WG-A (0.037) Is (1.00,0.01) C16H10OSBr2H
National Mass Spectrometry Facility, Swansea
Xevo G2-S
Sianne Schwikkard
16-Aug-2019
1: TOF MS ASAP+
4.11e12
Theoretical Isotope Pattern (M+H)
408.8897
409.8929
410.8877
411.8909
412.8857
413.8888
414.8846
415.8862
%
m/z
KINSCH-L4UNJ-WG-A 187 (1.746) AM (Cen,4, 80.00, Ar,10000.0,0.00,0.00); Cm (186:205)
1: TOF MS ASAP+
4.17e6
Observed Data
406.8746
407.8818
408.8896
409.8907
410.8876
410.9417
411.8902
412.8856
413.8887
414.8843
415.8864

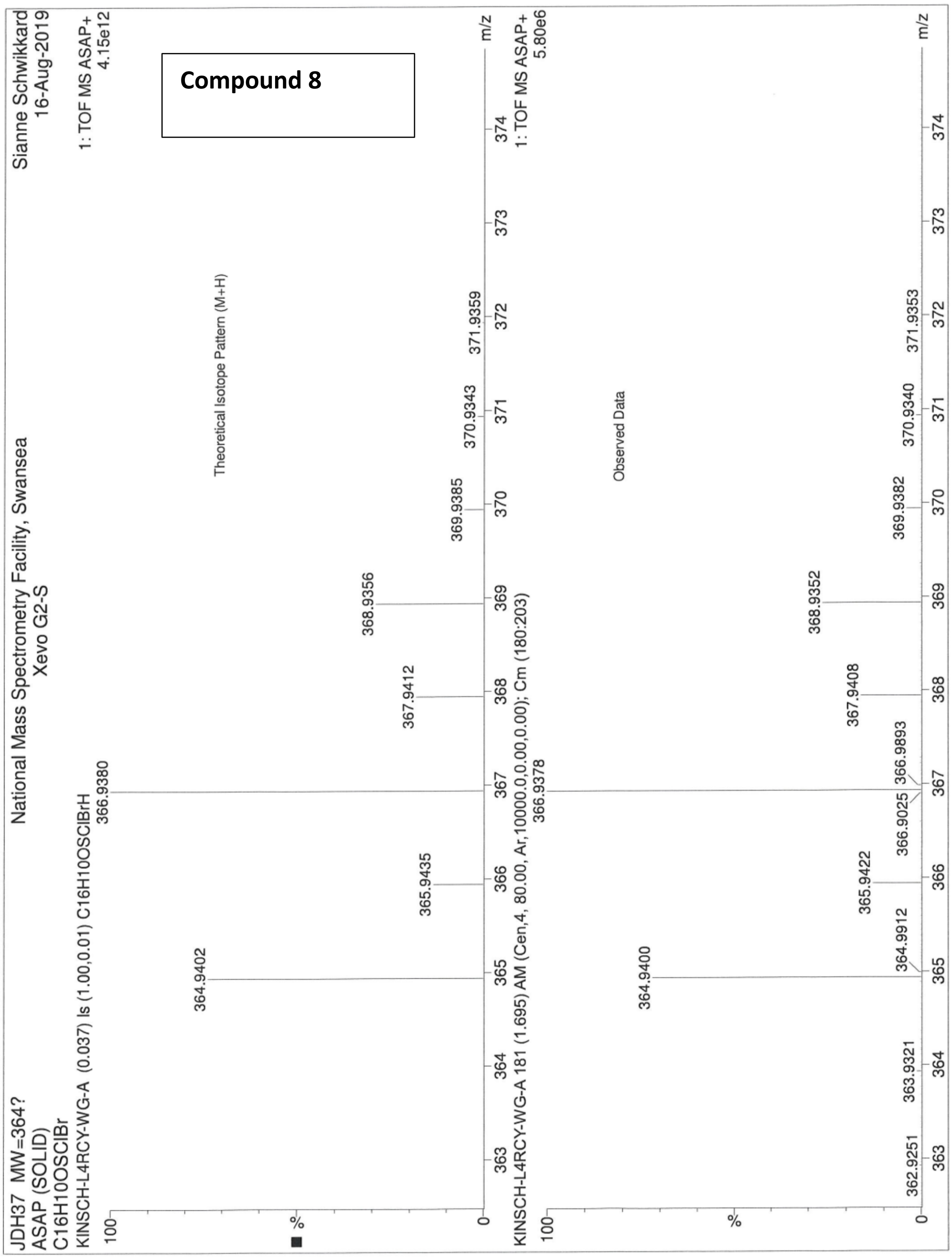

Compound 8
JDH37 MW=364?
ASAP (SOLID)
C16H10OSClBr
National Mass Spectrometry Facility, Swansea
Xevo G2-S
Sianne Schwikkard
16-Aug-2019
KINSCH-L4RCY-WG-A (0.037) Is (1.00,0.01) C16H10OSClBrH
1: TOF MS ASAP+
4.15e12
Theoretical Isotope Pattern (M+H)
364.9402
365.9435
366.9380
367.9412
368.9356
369.9385
370.9343
371.9359
KINSCH-L4RCY-WG-A 181 (1.695) AM (Cen,4, 80.00, Ar,10000.0,0.00,0.00); Cm (180:203)
1: TOF MS ASAP+
5.80e6
Observed Data
362.9251
363.9321
364.9400
364.9912
365.9422
366.9025
366.9378
366.9893
367.9408
368.9352
369.9382
370.9340
371.9353
%
m/z

Compound 9

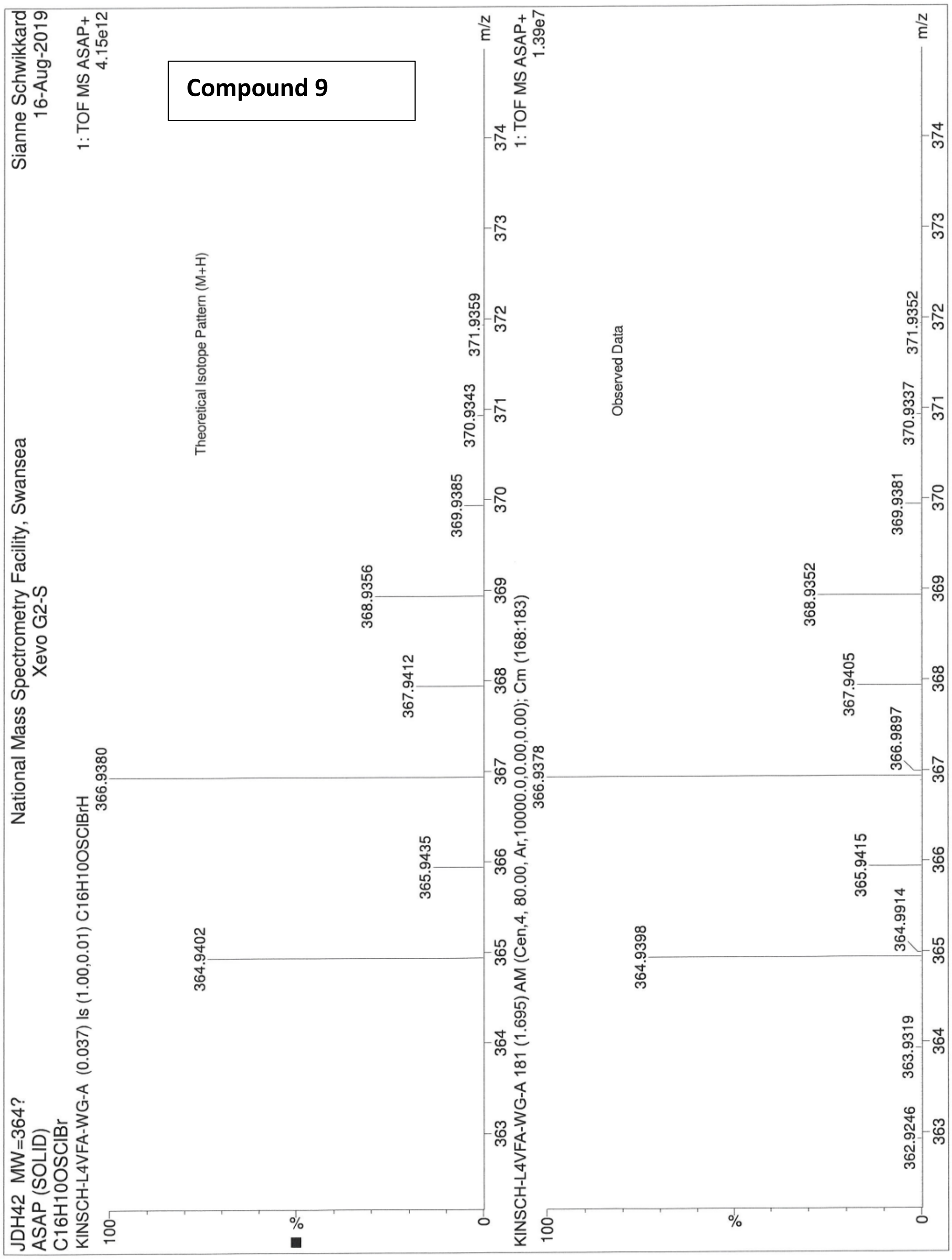

JDH42 MW=364?
ASAP (SOLID)
C16H10OSClBr
National Mass Spectrometry Facility, Swansea
Xevo G2-S
Sianne Schwikkard
16-Aug-2019
KINSCH-L4VFA-WG-A (0.037) Is (1.00,0.01) C16H10OSClBrH
1: TOF MS ASAP+
4.15e12
Theoretical Isotope Pattern (M+H)
364.9402
365.9435
366.9380
367.9412
368.9356
369.9385
370.9343
371.9359
KINSCH-L4VFA-WG-A 181 (1.695) AM (Cen,4, 80.00, Ar,10000.0,0.00,0.00); Cm (168:183)
1: TOF MS ASAP+
1.39e7
Observed Data
362.9246
363.9319
364.9398
364.9914
365.9415
366.9378
366.9897
367.9405
368.9352
369.9381
370.9337
371.9352
%
m/z

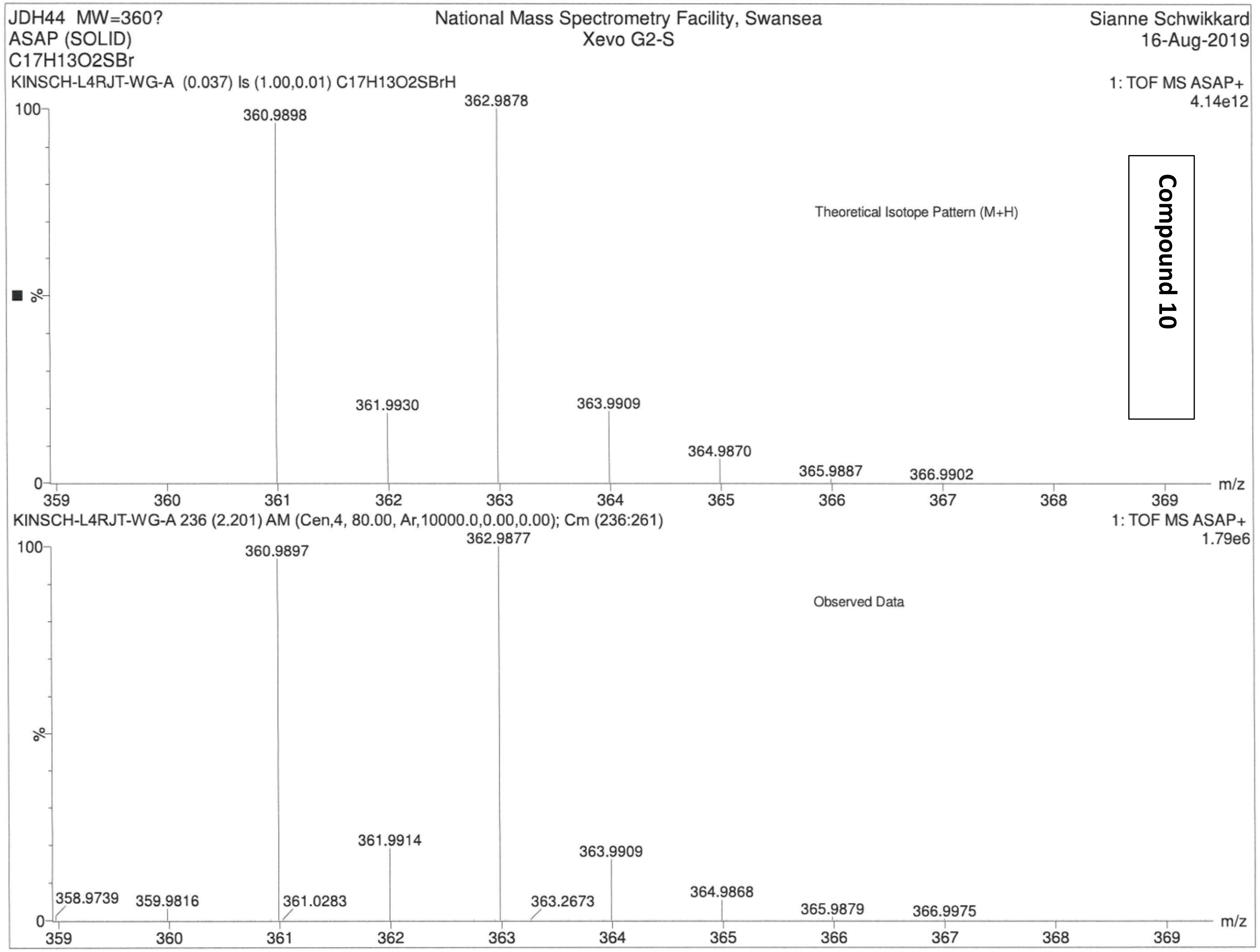

JDH44 MW=360?
ASAP (SOLID)
C17H13O2SBr
National Mass Spectrometry Facility, Swansea
Xevo G2-S
Sianne Schwikkard
16-Aug-2019
KINSCH-L4RJT-WG-A (0.037) Is (1.00,0.01) C17H13O2SBrH
1: TOF MS ASAP+
4.14e12
Compound 10
Theoretical Isotope Pattern (M+H)
360.9898
362.9878
361.9930
363.9909
364.9870
365.9887
366.9902
KINSCH-L4RJT-WG-A 236 (2.201) AM (Cen,4, 80.00, Ar,10000.0,0.00,0.00); Cm (236:261)
1: TOF MS ASAP+
1.79e6
Observed Data
360.9897
362.9877
361.9914
363.9909
358.9739
359.9816
361.0283
363.2673
364.9868
365.9879
366.9975
100
%
0
m/z
359
360
361
362
363
364
365
366
367
368
369

Compound 11

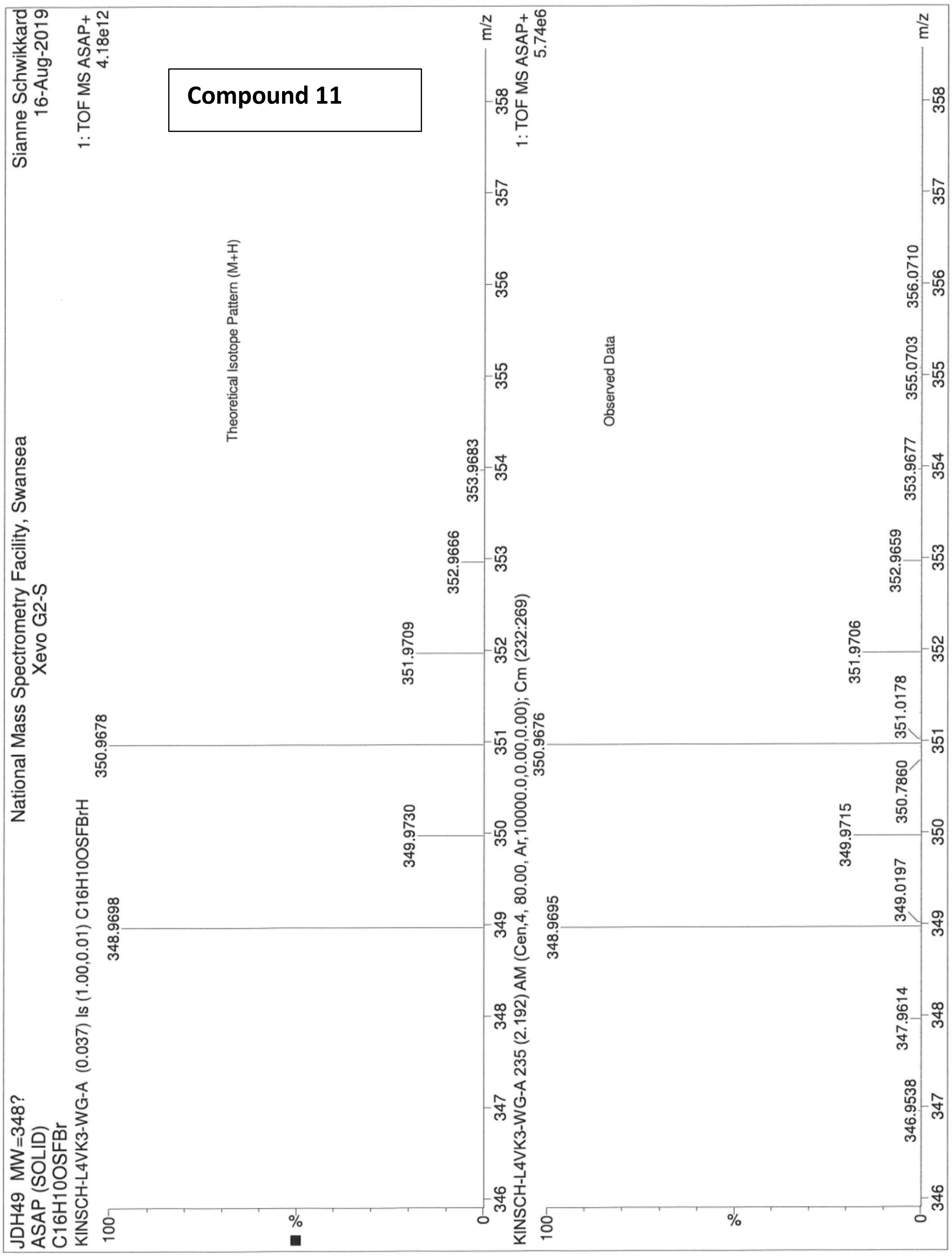
JDH49 MW=348?
ASAP (SOLID)
C16H10OSFBr
KINSCH-L4VK3-WG-A (0.037) Is (1.00,0.01) C16H10OSFBrH
National Mass Spectrometry Facility, Swansea
Xevo G2-S
Sianne Schwikkard
16-Aug-2019
1: TOF MS ASAP+
4.18e12
Theoretical Isotope Pattern (M+H)
348.9698
349.9730
350.9678
351.9709
352.9666
353.9683
KINSCH-L4VK3-WG-A 235 (2.192) AM (Cen,4, 80.00, Ar,10000.0,0.00,0.00); Cm (232:269)
1: TOF MS ASAP+
5.74e6
Observed Data
346.9538
347.9614
348.9695
349.0197
349.9715
350.7860
350.9676
351.0178
351.9706
352.9659
353.9677
355.0703
356.0710
m/z
%

Compound 12

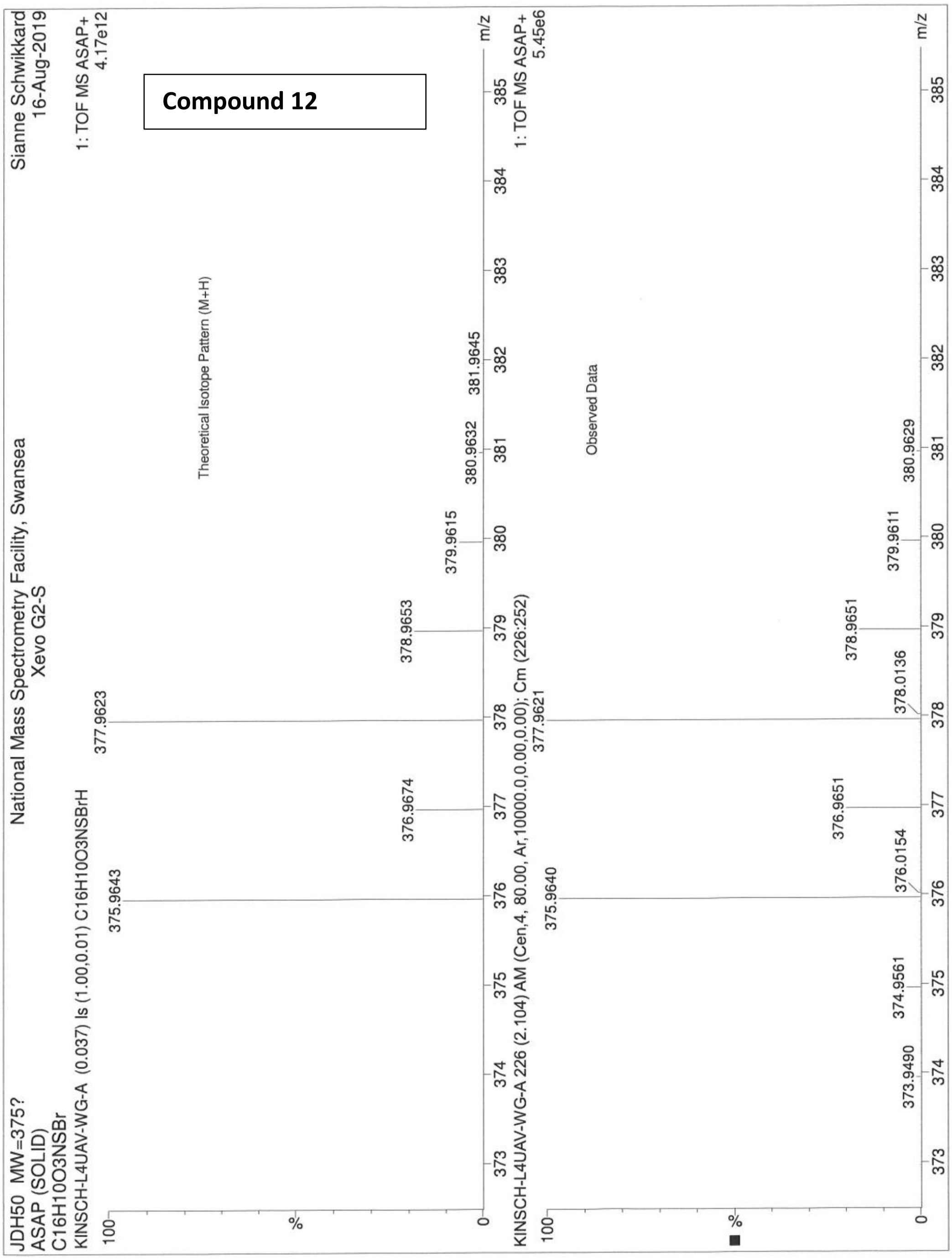
JDH50 MW=375?
ASAP (SOLID)
C16H10O3NSBr
National Mass Spectrometry Facility, Swansea
Xevo G2-S
Sianne Schwikkard
16-Aug-2019
KINSCH-L4UAV-WG-A (0.037) Is (1.00,0.01) C16H10O3NSBrH
1: TOF MS ASAP+
4.17e12
Theoretical Isotope Pattern (M+H)
375.9643
376.9674
377.9623
378.9653
379.9615
380.9632
381.9645
KINSCH-L4UAV-WG-A 226 (2.104) AM (Cen,4, 80.00, Ar,10000.0,0.00,0.00); Cm (226:252)
1: TOF MS ASAP+
5.45e6
Observed Data
373.9490
374.9561
375.9640
376.0154
376.9651
377.9621
378.0136
378.9651
379.9611
380.9629
%
100
0
m/z
373
374
375
376
377
378
379
380
381
382
383
384
385

Compound 13

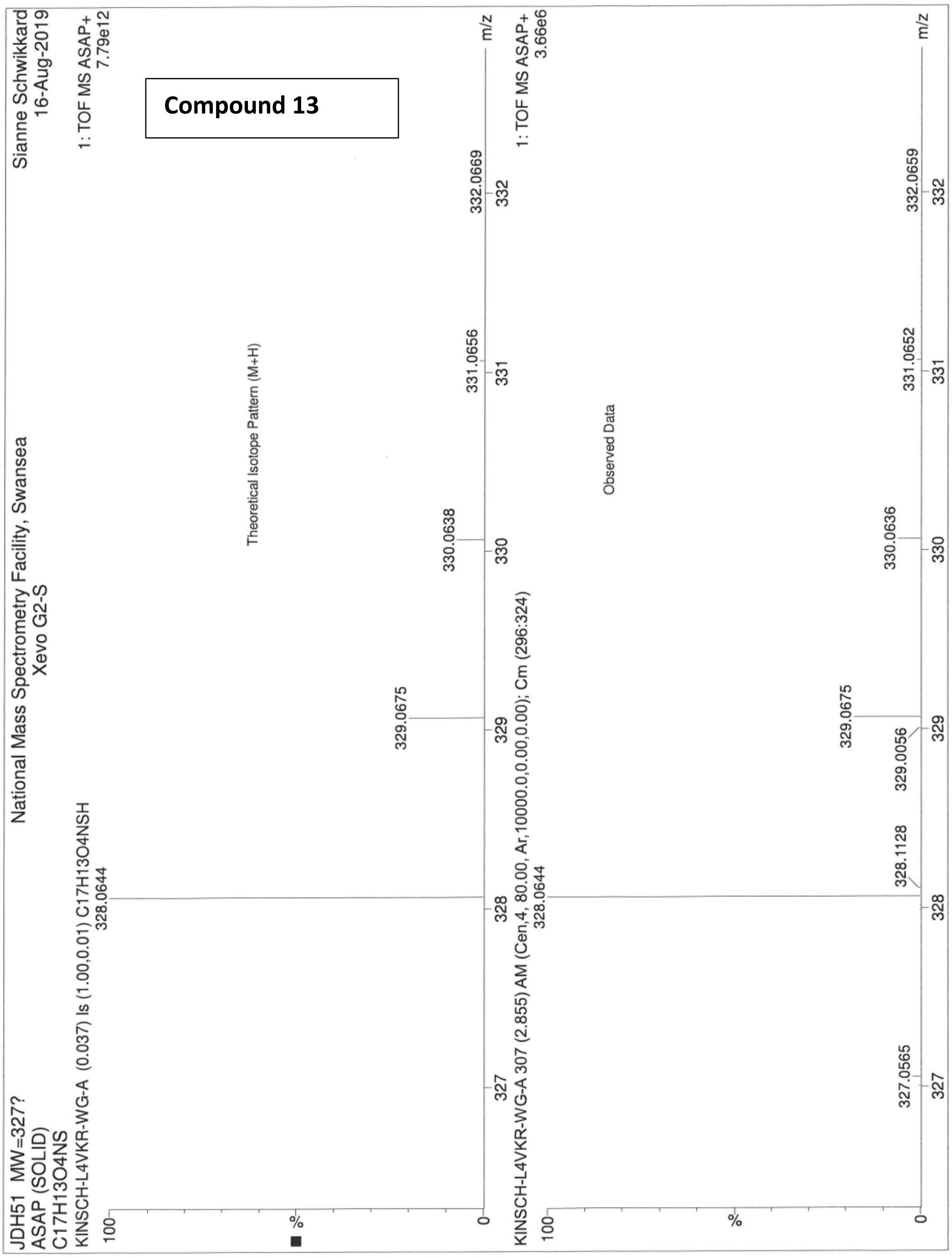
JDH51 MW=327?
ASAP (SOLID)
C17H13O4NS
National Mass Spectrometry Facility, Swansea
Xevo G2-S
Sianne Schwikkard
16-Aug-2019
KINSCH-L4VKR-WG-A (0.037) Is (1.00,0.01) C17H13O4NSH
1: TOF MS ASAP+
7.79e12
Theoretical Isotope Pattern (M+H)
328.0644
329.0675
330.0638
331.0656
332.0669
KINSCH-L4VKR-WG-A 307 (2.855) AM (Cen,4, 80.00, Ar,10000.0,0.00,0.00); Cm (296:324)
1: TOF MS ASAP+
3.66e6
Observed Data
327.0565
328.0644
328.1128
329.0056
329.0675
330.0636
331.0652
332.0659
m/z
%
